# Informed Consent Disclosures and Minimum Requirements in AI Clinical Trials: Cross-Sectional Analysis

**DOI:** 10.2196/94504

**Published:** 2026-07-02

**Authors:** Hankun Su, Fen Xiao, Hoksan Chau, Yuqian Tong, Siyi Han, Xinyu Cheng, Zhilin Che, Liye Sun, Yuemeng Yang, Jing Zhao, Yanping Li, Hui Li

**Affiliations:** 1 Department of Reproductive Medicine Xiangya Hospital Central South University Changsha, Hunan China; 2 Clinical Research Center for Women's Reproductive Health in Hunan Province Changsha, Hunan China

**Keywords:** artificial intelligence, AI, informed consent, patient education, health literacy

## Abstract

**Background:**

The integration of artificial intelligence (AI) into clinical research challenges traditional informed consent (IC) frameworks due to the algorithmic complexity, opacity, and adaptive nature of AI systems. Although public demand for transparency regarding AI use in health care is high, current ethical guidelines lack specificity, and there has been no assessment of AI representation in IC documentation within clinical trial registries.

**Objective:**

This study aimed to evaluate the prevalence, clarity, and completeness of AI-related consent disclosures in clinical trials registered on ClinicalTrials.gov and to propose a framework for enhanced patient digital literacy and ethical robustness.

**Methods:**

We conducted a cross-sectional content analysis of 114 AI-involving clinical trials with publicly available IC documents from ClinicalTrials.gov (search conducted on June 21, 2025). We assessed AI-specific disclosures, readability (using the Simple Measure of Gobbledygook index), document length, visual aid use, and data governance protocols against World Health Organization and National Institutes of Health standards. We also refined an AI risk framework encompassing model autonomy, deviation from standards of care, patient-facing interaction, and clinical risk, scoring each trial on a 3-tier scale.

**Results:**

More than half (66/114, 58%) of ICs failed to disclose the AI type or its intended use, and 18.4% (n=21) omitted risks entirely. Discrepancy was observed between trial registry entries and IC reporting of AI methods. Only 14% (n=16) of ICs met the dual criteria of brevity (<15,000 characters) and readability (Simple Measure of Gobbledygook <13). Higher-risk trials did not demonstrate improved readability (Spearman correlation *P*>.05). Only 11.4% (n=13) of ICs included visual aids, and their inclusion was not correlated with lower reading difficulty. Data handling protocols after participant withdrawal were inconsistent: 51 (44.7%) ICs provided no information, 30 (26.3%) specified data destruction, 29 (25.4%) allowed continued use, and only 4 (3.5%) offered participants a choice. Cited data protection laws varied widely, with no dominant standard.

**Conclusions:**

Current IC practices in AI clinical trials registered on ClinicalTrials.gov show a notable disconnect from ethical principles, with deficits in transparency, readability, and participant control over data. Our findings indicate a need for more standardized, participant-centered consent practices. We propose the “Minimum Requirements for Informed Consent in AI‑related Clinical Trials” as a possible framework to improve consent quality. However, it should be noted that these findings are limited to publicly available consent documents in the registry and may differ from final onsite versions.

## Introduction

The integration of artificial intelligence (AI) into clinical research and health care is accelerating, enabling improved diagnostics, personalized therapies, and operational efficiency [1]. As AI systems take on increasingly autonomous roles in medical decision-making, ensuring patient autonomy through informed consent (IC) has become a pressing ethical priority [2]. Traditional IC frameworks require clear disclosure of study procedures, risks, benefits, and data use; however, the complexity, opacity, and adaptive nature of AI challenge these principles, making meaningful comprehension difficult [3]. Current ethical guidelines lack specificity regarding what AI-related information should be disclosed, resulting in inconsistent practices across institutions and jurisdictions [3]. Although some advocate for risk-adjusted disclosures based on AI autonomy and clinical impact, others rely on general institutional notices, leaving participants potentially uninformed about how AI affects their care or data [4].

Public expectations further highlight this gap. A recent US national survey found that more than 80% of respondents believe they should be notified whenever AI is used in their health care, regardless of risk level [5]. This widespread demand for transparency underscores a growing need for accountability and trust in AI-augmented medicine. Yet, traditional consent forms—often static, text-heavy, and technically dense—fail to communicate complex AI processes effectively [6]. Research in human-computer interaction shows that dynamic, interactive models such as layered disclosures, just-in-time information, and AI-powered chatbots improve understanding and engagement in digital contexts [7]. These approaches offer promising strategies for enhancing consent in AI-driven trials, but their real-world implementation remains unexamined.

To date, there has been no global assessment of how AI is represented in IC documentation within clinical trial registries. This study fills that gap by conducting a cross-sectional analysis of registered clinical trials to evaluate the prevalence, clarity, and completeness of AI-related consent disclosures. Our primary objectives are (1) to determine whether IC documents in AI-involved trials disclose essential information regarding the role and function of AI; (2) to evaluate whether these documents adhere to established standards of brevity and readability, thereby supporting genuine participant comprehension in light of evidence that lengthy, complex forms undermine informed decision-making; and (3) to analyze how consent materials inform participants about the handling, security, and governance of their data. We also examine variations across trial designs, therapeutic areas, and degrees of AI involvement. Our findings aim to inform policy, guide institutional review boards, and support the development of standardized, ethically robust consent practices aligned with both participant expectations and the evolving realities of AI in clinical research.

## Methods

### Overview

This cross-sectional analysis was conducted according to the Joanna Briggs Institute Manual for Evidence Synthesis and adheres to the STROBE (Strengthening the Reporting of Observational Studies in Epidemiology) checklist [8]. This analysis was preregistered on the Open Science Framework platform [9].

### Study Design

We conducted a cross-sectional content analysis of clinical trial registry entries to assess the extent, quality, and clarity of IC disclosures related to AI in human subject research. The unit of analysis was the individual trial record and its related IC.

### Participants

There were no human participants in this study. ClinicalTrials.gov was searched as the primary database because it is the only clinical trial registry that provides publicly available IC documents; other registries do not offer such materials (the search strategy is provided in Multimedia Appendix 1). In addition, we conducted online searches to identify any potentially available IC documents to ensure comprehensive data collection. The search strategy was developed based on existing systematic reviews and scoping reviews [10,11] (Multimedia Appendix 1).

For the purpose of this study, an AI-involved clinical trial was defined as any trial that explicitly reported the use of AI, machine learning, deep learning, natural language processing, computer vision, large language models, or any other algorithmic tool intended to perform tasks typically requiring human intelligence. This definition was applied during both screening and full-text eligibility assessment.

The search was conducted on June 21, 2025. Two authors (HC and SH) independently screened potentially relevant clinical trials for the availability of IC documents. Because registries use varied and sometimes imprecise AI terminology, we adopted a multistep approach. First, 2 reviewers independently screened all records. Second, any trial mentioning at least 1 AI-related keyword (eg, “artificial intelligence,” “machine learning,” or “large language model”) was flagged for full review. For ambiguous cases, we consulted the full study protocol or linked publications when available. Disagreements were resolved by a senior researcher (HS). The eligibility criteria are defined by the following inclusion and exclusion criteria.

Inclusion criteria include (1) clinical trials involving the use of AI and (2) clinical trials with IC documents available. Exclusion criteria include (1) nonhuman research, (2) duplicate records (ie, the same trial listed across multiple platforms), and (3) records with severely redacted or incomplete IC documents that prevented assessment.

### Variables and Data Collection Methods

Basic information about each clinical trial was systematically extracted using a standardized data collection template. This included key trial characteristics such as National Clinical Trial number; title; year first posted; principal investigator; sponsor; country; trial status; phase; enrollment size; medical specialty; study type; and specific details regarding AI implementation, including whether commercially available AI was used, the type and purpose of AI (eg, diagnostic support or risk prediction), the level of model autonomy, supervision by clinicians, and associated clinical risk.

In parallel, ICs were analyzed using a dedicated extraction form to evaluate three core ethical and communicative dimensions: (1) basic information disclosure, assessed against international standards from the World Health Organization and the US National Institutes of Health, including transparency about the trial’s identity, institutional oversight, ethics approval, and the AI system’s role [12]; (2) readability, measured using the SMOG (Simple Measure of Gobbledygook) index to estimate the grade level required for comprehension, with attention to document length, use of plain language, and inclusion of visual aids; and (3) data protection, encompassing disclosures about data use, storage practices, participant rights regarding data control (eg, options to personalize or withdraw consent for data use), references to relevant government or regulatory policies, and clarity regarding data privacy safeguards.

This dual-extraction approach enabled a comprehensive assessment of how IC documents in AI-related trials communicate essential ethical and procedural information to potential participants, and whether current IC practices align with both regulatory expectations and participant-centered principles of transparency and accessibility.

All data were extracted using standardized forms developed in Microsoft Excel, designed to ensure consistency across reviewers. Five researchers (HS, HC, YQ, XC, and ZC) independently extracted data from clinical trials and IC documents. To ensure reliability, a pilot test was conducted on 5% (6/114) of randomly selected IC documents, demonstrating high interrater agreement (intraclass correlation coefficient=0.77). Discrepancies during data extraction were resolved through adjudication by a senior researcher (SH).

### Statistical Analysis

The collected raw data were first imported into Microsoft Excel 2019 for preliminary collation. Any discrepancies were reassessed by a senior researcher (SH). For descriptive statistics, we report frequencies and percentages for categorical characteristics (IC documents were counted in multiple categories if they covered more than one field) and medians with IQRs for continuous characteristics.

To assess the risks and involvement of AI in clinical trials, we adapted and refined an existing framework for AI use, informed by our extracted data and supported by established literature [4,13] (Multimedia Appendix 1). Specifically, the AI-related risk score was categorized across four interrelated dimensions: (1) model autonomy, ranging from AI used solely for data processing to systems whose outputs are indispensable for clinical decisions; (2) deviation from standards of care, ranging from adherence to established guidelines to AI-driven interventions that meaningfully diverge from conventional practice; (3) patient-facing interaction, ranging from minimal advisory functions to highly interactive, personalized AI interfaces; and (4) clinical risk, encompassing the potential for physical, diagnostic, or therapeutic harm, particularly in invasive or high-stakes applications. Each dimension was scored on a 3-tier scale (levels 1-3), with higher levels indicating greater ethical and clinical risk.

To assess the readability of IC documents, we evaluated 2 dimensions: length and linguistic complexity. Document length was measured in characters, with a maximum acceptable reading time of 10 minutes based on a conservative reading speed of 1500 characters per minute (equivalent to approximately 240 words per minute) [14]. Linguistic complexity was assessed using the SMOG index, a validated tool well suited for biomedical texts [15]. The calculation for the SMOG index is 1.0430×**√**(total polysyllables×[30÷total sentences])+3.1291. Higher scores indicate greater reading difficulty. In this study, a SMOG score of 13 was used as the upper threshold, corresponding to the lowest level of college reading demand; documents exceeding this level are unlikely to be fully comprehensible to the general public, whose average reading proficiency in the United States is at or below the college level [16]. To evaluate the association between IC readability and the AI‑related risk score, the Spearman rank correlation coefficient was used to assess the monotonic association. Post hoc pairwise comparisons were conducted using the Dunn test with Bonferroni correction for multiple testing. A 2‑sided *P* value <.05 was considered indicative of statistical significance.

Statistical analysis was performed with R software (version 4.2.2; R Foundation for Statistical Computing). BioRender (BioRender.com) and GraphPad Prism (version 9; GraphPad Software Inc) were used to draw figures and graphs.

### Expert Validation

To ensure the content validity, clarity, and practical applicability of the author-developed instruments (the AI risk scoring framework and the Minimum Requirements for Informed Consent in AI-Related Clinical Trials [MRIC-AI] checklist), we conducted a formal expert validation process. A panel of 4 experts with diverse expertise in medicine (n=3) and law (n=1) was gathered to evaluate the instruments (refer to the Acknowledgments section for details).

For the AI risk scoring framework, a 4D framework (model autonomy, deviation from standards of care, patient-facing interaction, and clinical risk) was initially derived from established literature [4,13]. The experts assessed the relevance, comprehensiveness, and operational clarity of each dimension. Each expert received a structured evaluation form and rated every dimension on a 5-point Likert scale, with 1 indicating “strongly disagree” and 5 showing “strongly agree.” The mean score and SD were calculated for each dimension.

For the MRIC-AI checklist, the 15 proposed minimum requirements were evaluated by each expert using a structured evaluation form. Experts rated every requirement on a 5-point Likert scale across four criteria: (1) necessity as a minimum standard for AI-related trials; (2) clarity and operationalizability for researchers, institutional review boards, and participants; (3) feasibility across different trial phases, geographies, and regulatory contexts; and (4) alignment with core ethical principles and emerging AI and data governance standards. Experts were also invited to provide free-text comments. The mean score and SD were calculated for each requirement. All feedback was considered, and minor wording adjustments were made. The final checklist reflects expert consensus.

### Ethical Considerations

Ethics board review was deemed unnecessary by the ethics committee of Xiangya Hospital because the data used in this study were obtained from publicly available sources and did not directly involve human participants or animals. This determination is consistent with the National Health Commission of the People’s Republic of China (Order No. 4, 2023).

## Results

### Representativeness of Included Clinical Trials

We identified 114 AI-related clinical trials with available IC documents (the selection process is available in Multimedia Appendix 1). We analyzed their registration information in clinical trial registry platforms to confirm their representativeness (detailed data for individual trials are available in Multimedia Appendix 1).

**Figure 1:**
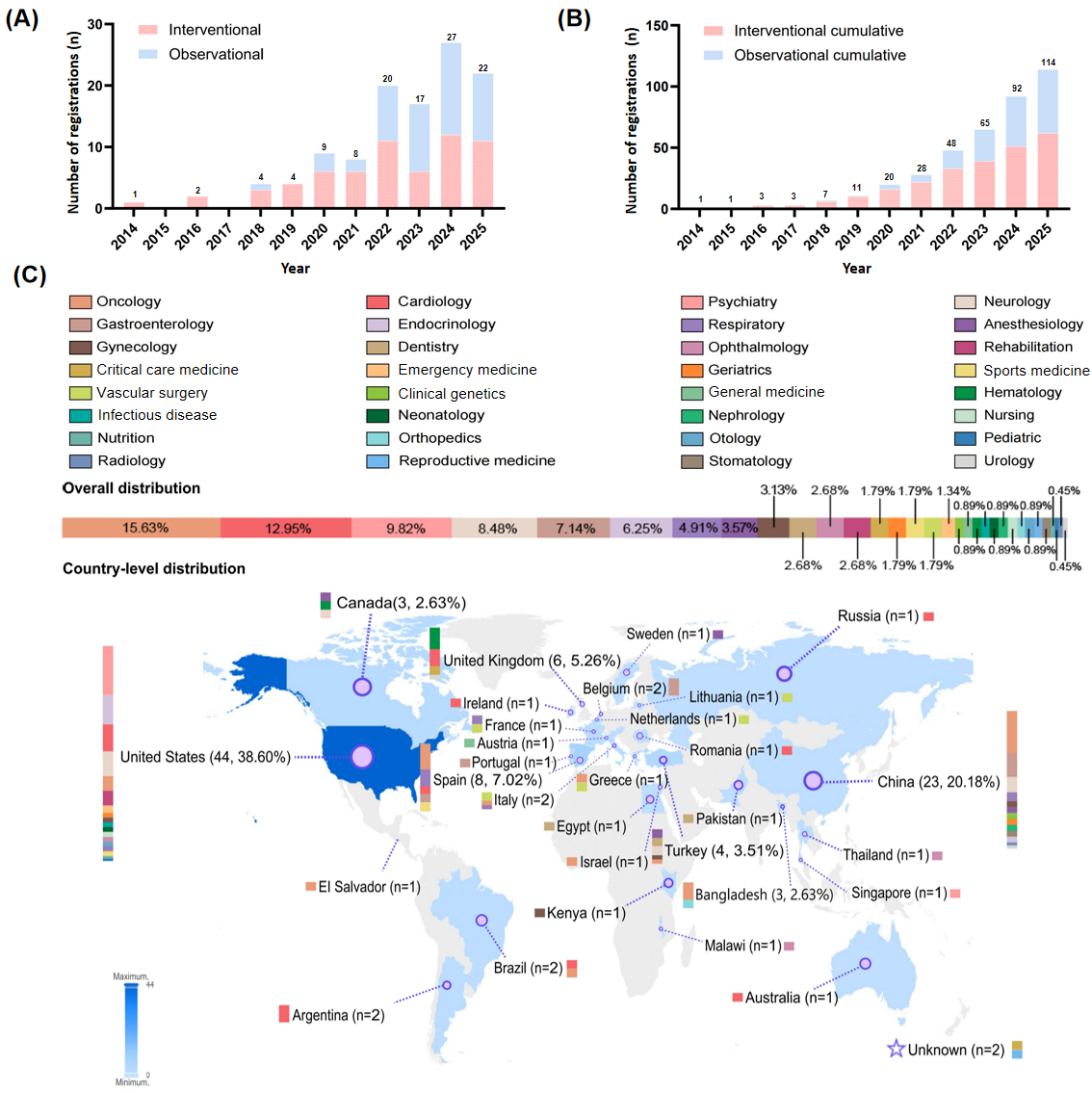
Overview of the included clinical trials. (A) Annual registration of trials, (B) cumulative registration of trials, and (C) distribution of clinical trials across regions and clinical domains. Informed consent documents were counted in multiple countries if they covered more than one country; therefore, total number may exceed 114.

Figures 1A and 1B show the registration timeline of the 114 trials, demonstrating a significant increase in recent years. Of the 114 clinical trials, 32 clinical domains were involved, covering most medical specialties. A total of 19 (16.7%) trials were related to oncology, 15 (13.2%) to cardiology, 11 (9.6%) to psychiatry, and 10 (8.8%) to neurology (Figure 1C).

**Figure 2:**
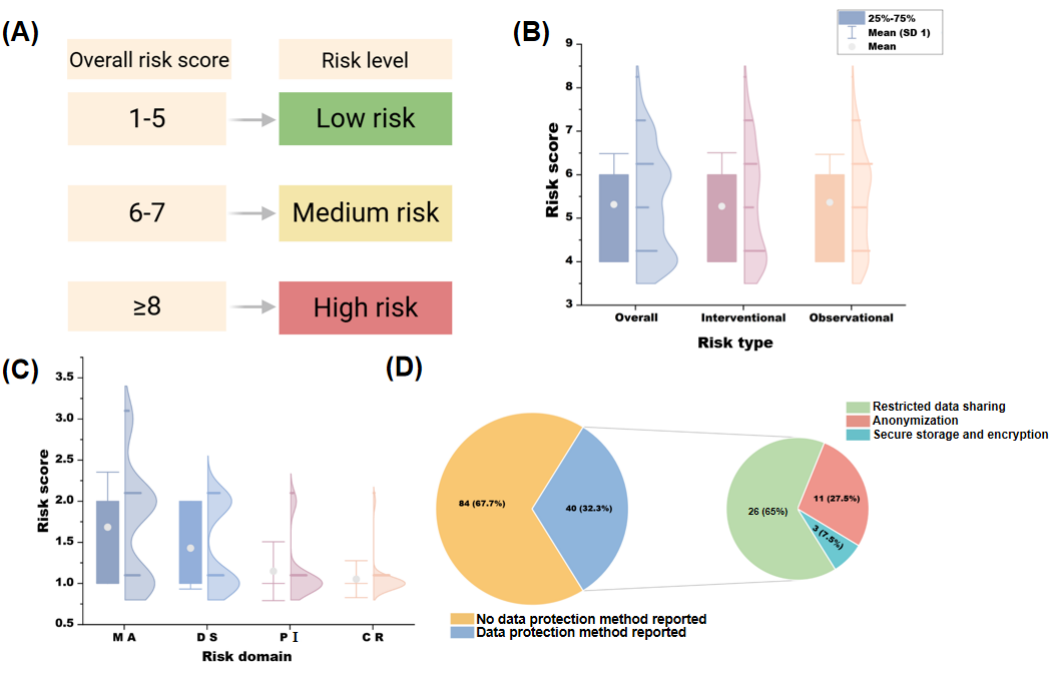
Risk and data protection methods of the included clinical trials. (A) Illustration of the risk score and associated risk levels, (B) overall risk scores of the included clinical trials, (C) risk scores by specific dimensions, and (D) data protection methods stated in the trial registry platform. CR: clinical risk; DS: deviation from standards of care; MA: model autonomy; PI: patient-facing interaction. Informed consent documents were counted in multiple categories if they covered more than one category; therefore, total number may exceed 114.

Further examination of the risk of each trial based on predefined categories (Figure 2A) showed that the included trials had a median overall risk score of 5 (IQR 4-6); among the trials, 3 (2.6%) were high risk, 49 (43%) were medium risk, and 62 (54.4%) were low risk. Stratification by interventional vs observational study design revealed similar risk distributions (mean score for interventional trials 5.3, SD 1.2; mean score for observational trials 5.4, SD 1.2). Additionally, model autonomy risk showed greater variability, while clinical risk scores were mostly consistent (Figures 2B and 2C).

A total of 37 (32.5%) trials involving AI were conducted under the supervision of external physicians, while 77 (67.5%) trials were conducted without physician supervision. Overall, 58 (50.9%) trials mentioned signed IC as part of their basic inclusion and exclusion criteria, while 56 (49.1%) trials did not (Table 1).

The most commonly described AI technology in the trials was machine learning (39/92, 42.4%), followed by deep learning (n=19, 20.7%) and a general description of “AI-specific tools or scenario-based algorithms” (n=15, 16.3%; Table 1). Regarding AI-related data protection methods, only 40 (32.3%) provided specific measures to protect sensitive data, with most referring to restricted data sharing and anonymization (Figure 2D).

**Table 1:** General characteristics of the included trials (N=114).

Characteristics		Trials, n (%)
**Risk distribution of trials**		
	Low risk	62 (54.4)
	Medium risk	49 (43)
	High risk	3 (2.6)
**AI^a^ supervision in trial**		
	Without supervision	77 (67.5)
	With supervision	37 (32.5)
**Informed consent included in eligibility criteria**		
	Mentioned	58 (50.9)
	Not mentioned	56 (49.1)
**Disclosure of AI type in trial design^b^**		
	Machine learning	39 (42.4)
	Deep learning	19 (20.7)
	AI-specific tools or scenario algorithms	15 (16.3)
	Convolutional neural network	7 (7.6)
	Natural language processing	5 (5.4)
	Reinforcement learning	4 (4.3)
	Large language model	2 (2.2)
	Human-in-loop	1 (1.1)

^a^AI: artificial intelligence.

^b^Trials without specific AI type disclosure were excluded from this calculation to avoid ambiguity (N=92).

### Analysis of Necessary Information Disclosure in IC Documents

**Figure 3:**
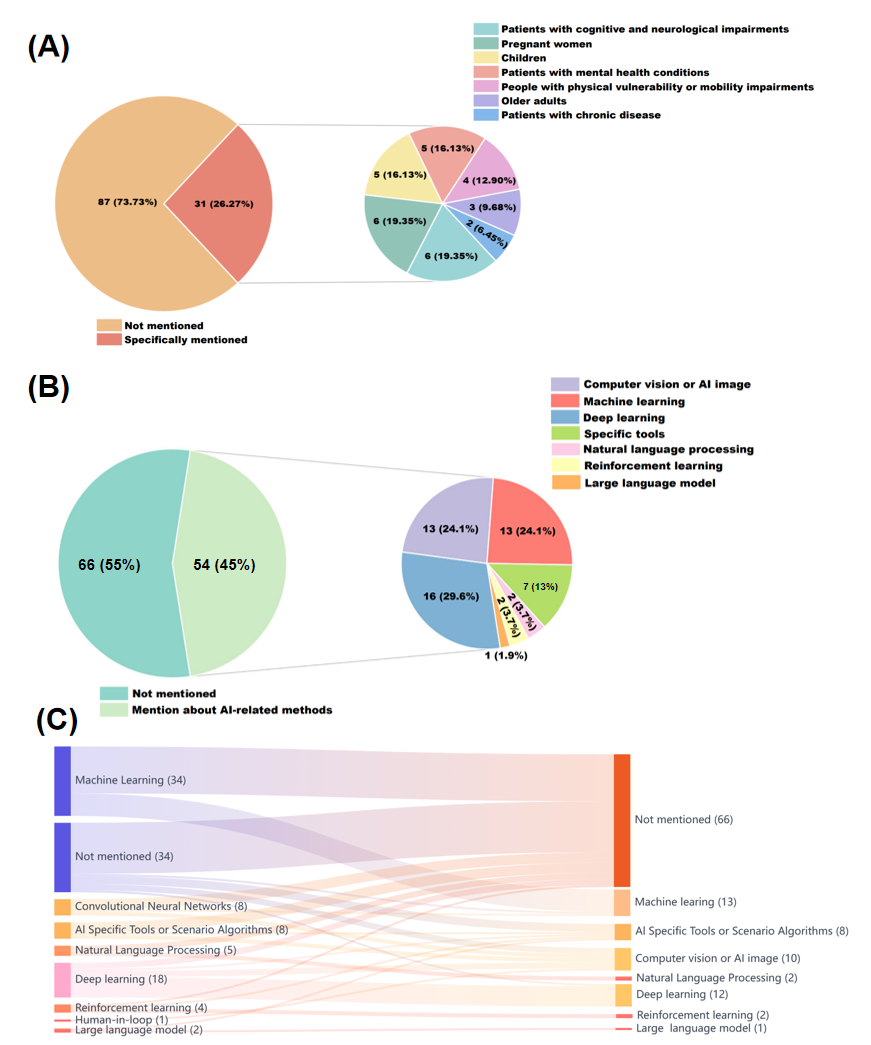
Necessary information disclosed in informed consent (IC) documents. (A) Vulnerable population disclosed for protection in IC documents, (B) types of artificial intelligence (AI) disclosed in IC documents, and (C) Sankey plot of AI descriptions in trial registries and IC documents (the left side represents data from trial registries, and the right side represents data from IC documents). IC documents were counted in multiple categories if they covered more than one category; therefore, total number may exceed 114.

The ICs were extracted from the 114 included trials; detailed characteristics are provided in Multimedia Appendix 1. We examined the necessary information disclosed in IC documents according to World Health Organization and National Institutes of Health guidelines. Results are provided in Figure 3 and Table 2.

Although 88.6% (101/114) of ICs disclosed potential benefits, 11.4% (n=13) failed to mention any benefits at all, with the most commonly reported advantages being clinical and diagnostic improvements (n=25, 21.9%), treatment optimization (n=21, 18.4%), and participant well-being (n=18, 15.8%). Conversely, the disclosure of risks was more deficient, as 18.4% (n=21) of ICs omitted any mention of risks entirely, and privacy and confidentiality concerns (n=26, 22.8%) were the most frequently noted among those that did report risks. Most ICs (n=98, 85.96%) included both basic study information and confirmation of ethics approval; however, 14.04% (n=16) did not reference ethics review (Table 2).

Identification of vulnerable populations was inadequate, with 76.3% (87/114) of ICs failing to specify any such groups, despite the need for special protections. Additionally, transparency regarding trial methodology was lacking, as 58% (n=66) of ICs did not disclose the type or use of AI, leaving participants uninformed about the technological basis of the research (Figures 3A and 3B). Additionally, we identified a significant discrepancy in the reporting of AI methods between trial registries and IC documents. IC documents frequently failed to disclose the AI methodology (n=66, 58% not disclosed in IC documents vs n=34, 30% not disclosed in trial registries) or described it only vaguely, in contrast to the more detailed information typically found in trial registries (Figure 3C).

Although the right to withdraw was universally guaranteed in all ICs, the protocols for handling participant data after withdrawal were inconsistent. A total of 51 (44.7%) ICs provided no information on how patient data would be handled after withdrawal. Among the ICs that clarified, 30 (26.3%) specified that data would be destroyed upon withdrawal, 29 (25.4%) stated that already collected data would continue to be used, and only 4 (3.5%) offered participants a choice in the matter. An analysis of emergency contact information revealed that 15 (13.16%) IC forms lacked these details entirely. Among the forms that included an emergency contact, the role was assigned to the principal investigator in 54 (47.4%) ICs and to another member of the research team in 45 (39.5%) ICs (Table 2).

**Table 2:** Necessary information disclosure in informed consent documents (N=114).

Characteristics			Trials, n (%)
**Information disclosure on benefits**			
	Clinical and diagnostic improvements	25 (21.9)	
	Treatment and care optimization	21 (18.4)	
	Participant well-being and empowerment	18 (15.8)	
	Research and societal benefit	16 (14)	
	System and process efficiency	14 (12.3)	
	No direct participant benefit	7 (6.1)	
	Not applicable	13 (11.4)	
**Information disclosure on risks^a^**			
	No additional risks	42 (36.8)	
	Privacy and confidentiality risks	26 (22.8)	
	Clinical or physical safety risks	21 (18.4)	
	Psychological or emotional risks	15 (13.2)	
	Operational or technical risks	3 (2.6)	
	Not applicable	21 (18.4)	
**Disclosure on ethics approval**			
	Basic information and ethics approval	98 (86)	
	Basic information only	16 (14)	
**Postwithdrawal data handling policies**			
	Data destroyed after withdrawal	30 (26.3)	
	Continued use of previously collected data	29 (25.4)	
	Decided by participant	4 (3.5)	
	Not applicable	51 (44.7)	
**Emergency contact specified in the** **informed consent document**			
	Principal investigator	54 (47.4)	
	Member of the research team	45 (39.5)	
	Not applicable	15 (13.2)	

^a^Informed consent documents were counted in multiple categories if they covered more than one field; therefore, total percentage may exceed 100%.

### Readability and Comprehensiveness of IC Documents

**Figure 4:**
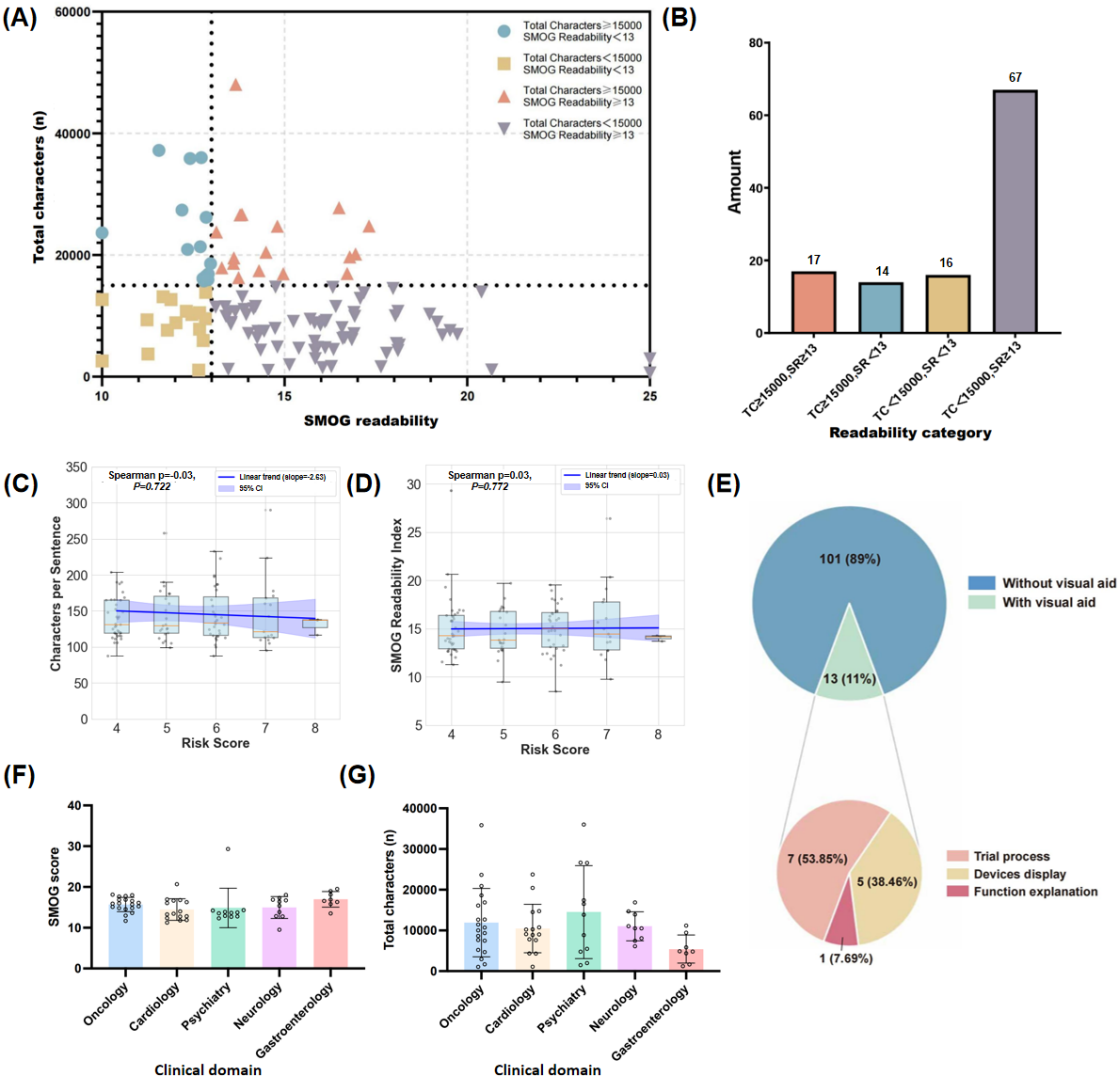
Readability and comprehensiveness of informed consent (IC) documents. (A) Quadrant analysis of the length and readability of IC documents; (B) distribution of IC documents by readability and length; (C) length of IC documents stratified by risk level, with Spearman rank correlation coefficient calculated; (D) readability of IC documents stratified by risk level, with Spearman rank correlation coefficients calculated; (E) percentage of supervised AI and unsupervised AI in clinical trials; (F) length of IC documents stratified by the top 5 clinical domains; and (G) readability of IC documents stratified by the top 5 clinical domains. SMOG: Simple Measure of Gobbledygook; SR: Simple Measure of Gobbledygook readability; TC: total characters.

Federal regulations on IC emphasize that documents should be brief, readable, and prioritize participants’ understanding [17]. Yet, the results revealed that only 14% (16/114) of current ICs met both criteria for brevity (fewer than 15,000 characters) and readability (SMOG score <13). Most consents were brief but difficult to read (character count <15,000; SMOG score ≥13; Figures 4A and 4B). Stratification by trial risk level showed an even more concerning trend: consent forms for higher-risk trials were not easier to understand (Spearman *P*>.05; Figures 4C and 4D). Further analysis across the top 5 clinical domains revealed inconsistent readability and length, with considerable variation in SMOG scores and overall length among domains (Figures 4F and 4G).

Additionally, only 11.4% (13/114) of the ICs included a visual aid to facilitate understanding. Among them, 7 (n=13, 53.8%) were used to illustrate the trial process, 5 (38.5%) were used to illustrate the device outlook and teach basic operational skills, and 1 (7.7%) was used to explain the function of AI (Figure 4E). Theoretical guidance suggests that visual aids should accompany complex IC forms. However, our data do not support this, as forms with visual aids demonstrated a lower, not higher, level of reading difficulty (SMOG score with visual aid 13.61, IQR 12.34-15.34 vs SMOG score without visual aid 14.52, IQR 12.96-16.80).

### Data Disclosure in IC Documents

The analysis of data types mentioned in ICs revealed a clear hierarchy in their prevalence. Clinical and medical history data were the most frequently referenced category (n=67, 58.8%), establishing this category as a core element of the studies. This was followed by imaging and biometric data (n=47, 41.2%) and demographic and contact information (n=35, 30.7%). Other data types, such as survey and self-reported data (n=24, 21%), biospecimens and derived data (n=23, 20.2%), and digital or device-generated sensor data (n=19, 16.7%), appeared less often (Table 3).

**Table 3:** Detailed information on data-related disclosures in informed consent (IC) documents (N=114).

Characteristics			Trials, n (%)
**Types of data explicitly mentioned in IC documents^a^**			
	Clinical and medical history data	67 (58.8)	
	Imaging and biometric data	47 (41.2)	
	Demographic and contact information	35 (30.7)	
	Survey, interview, and self-reported data	24 (21.1)	
	Biospecimens and derived data	23 (20.2)	
	Digital or device-generated sensor and use data	19 (16.7)	
	Not applicable	7 (6.1)	
**Data for future use of IC documents**			
	Consent waived	31 (27.2)	
	Consent required	23 (20.2)	
	No future use	8 (7)	
	Not applicable	52 (45.6)	
**Data storage disclosure in IC documents^a^**			
	Local storage	38 (33.3)	
	Institutional cloud server	31 (27.2)	
	Third-party cloud server	20 (17.5)	
	Not applicable	40 (35.1)	
**Cited data protection laws and regulations**			
	Certificate of Confidentiality	15 (13.2)	
	General Data Protection Regulation	12 (10.5)	
	Health Insurance Portability and Accountability Act	11 (9.6)	
	Organic Law 3/2018 (Spain)	6 (5.3)	

^a^IC documents were counted in multiple categories if they covered more than one field; therefore, total percentage may exceed 100%.

**Figure 5:**
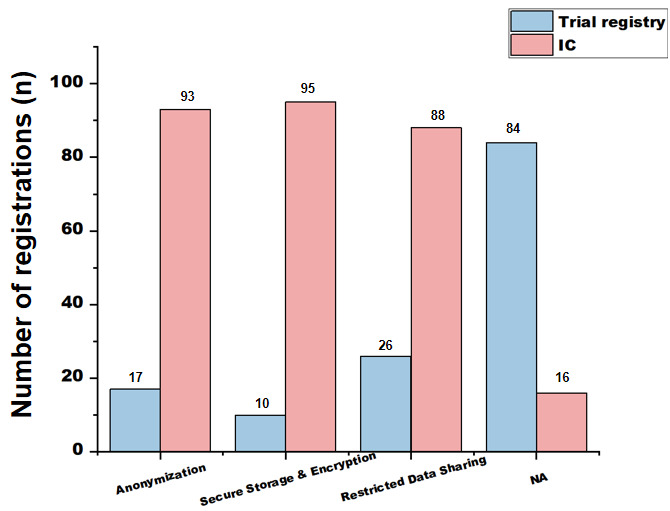
Discrepancies in data protection methods between trial registries and informed consent (IC) documents. NA: not applicable.

Regarding data security and storage provisions, local storage (38/114, 33.3%) and institutional cloud servers (n=31, 27.2%) were more commonly specified than third-party cloud servers (n=20, 17.5%; Table 3). There was also a significant discrepancy in the disclosure of data protection methods between trial registries and IC documents (Figure 5). For future research applications, data were most often designated for use without additional consent (n=31, 27.2%), with a smaller subset requiring such consent (n=23, 20.2%) and a minor portion explicitly barred from future use (n=8, 7%; Table 3).

Finally, the most cited data protection laws and frameworks were the Certificate of Confidentiality (n=15, 13.2%), the General Data Protection Regulation (GDPR; n=12, 10.5%), the Health Insurance Portability and Accountability Act (HIPAA; n=11, 9.6%), and Spain’s Organic Law 3/2018 (n=6, 5.3%; Table 3).

### Expert Validation Results

All 4 experts reviewed the AI risk scoring framework and MRIC-AI checklist independently (detailed data are provided in Multimedia Appendix 1). They confirmed that the AI risk scoring framework, with its 4 dimensions, collectively captured the key ethical and clinical risks of AI in clinical trials. The average Likert scale scores were 5 (SD 0) for model autonomy, 4.75 (SD 0.5) for deviation from standards, 4.75 (SD 0.5) for patient-facing interaction, and 5 (SD 0) for clinical risk.

For the MRIC-AI checklist, the mean scores were 4.25 (SD 0.87) for AI-specific disclosure, 4.83 (SD 0.39) for risk-benefit communication, 3.7 (SD 0.70) for readability and accessibility, 4.9 (SD 0.25) for data governance, and 3.9 (SD 0.67) for dynamic consent and support. No requirement was rated <3 (neutral) by any expert.

## Discussion

### Principal Findings

Our cross-sectional analysis of 114 AI-related clinical trials reveals a significant disconnection between established ethical principles for IC and current practices. Most IC documents fail to provide adequate transparency, readability, and data disclosure, undermining genuine participant comprehension and autonomy [5,18].

Our findings indicate that essential information about the AI system itself was frequently omitted. More than half (66/114, 58%) of the ICs did not disclose the type or specific use of AI, leaving participants uninformed about the core technological intervention at the heart of the research. This lack of transparency fundamentally undermines the principle of IC, as participants cannot consent to what they do not understand [12]. Furthermore, although most ICs disclosed potential benefits, the reporting of risks was deficient, with 18.4% (n=21) omitting any mention of risks entirely. This imbalance in risk-benefit communication could lead to a therapeutic misconception, whereby participants overestimate the potential for personal gain while underestimating the associated perils, particularly concerning data privacy.

The assessment of readability and comprehensiveness yielded equally troubling results. Only 14% (16/114) of IC documents met the dual criteria of brevity and readability. The widespread use of complex language (as evidenced by high SMOG scores) creates a substantial barrier to understanding, effectively excluding a large proportion of the general public. Notably, our data did not show that higher-risk trials used simpler consent forms; in fact, the opposite trend was observed. This suggests a critical failure to tailor communication to the ethical and clinical stakes involved, potentially exposing participants in the most sensitive trials to the greatest comprehension deficits.

Data governance and participant rights after withdrawal were also areas of concern because of substantial inconsistency. Although the right to withdraw was universally acknowledged, the protocols for handling data after withdrawal were highly variable, with only a small fraction (4/114, 3.5%) offering participants a choice in the matter. This ambiguity contravenes the principle of ongoing consent and participant control over personal data, a cornerstone of modern data protection regulations such as the GDPR. The heterogeneous citation of data protection laws further suggests the absence of standardized and robust data governance frameworks across trials.

### Minimum Requirements for Patient Education Regarding the Understanding of AI in Clinical Trials

To address the substantial gaps identified in our analysis and to ensure that IC documents fulfill their ethical purpose as educational tools, we propose a set of minimum requirements for disclosing AI-related information in clinical trials (Textbox 1). These requirements are grounded in the principles of transparency, accessibility, and participant autonomy and are designed to be actionable for researchers, institutional review boards, and policymakers.

**Checklist of minimum requirements for informed consent in artificial intelligence (AI)-related clinical trials.**

**AI-specific disclosure**

- Specify AI type, role, and level of autonomy
- Describe deviation from standard care, if applicable
- Explain patient-facing interactions, if relevant

**Risk-benefit communication**

- Disclose clinical, privacy, and algorithmic risks with equal prominence to benefits
- Contextualize benefits to avoid therapeutic misconception
- Acknowledge uncertainties and monitoring plans

**Readability and accessibility**

- Limit document length to ≤15,000 characters (approximately 10 minutes of reading time)
- Achieve a Simple Measure of Gobbledygook readability score ≤13 (≤10 for high-risk trials)
- Include at least 1 comprehension-tested visual aid
- Offer layered or interactive digital consent options, if feasible

**Data governance**

- Specify data types, storage, access, and retention
- Clarify postwithdrawal data handling and offer choice
- Disclose future data use and consent mechanisms
- Cite applicable protections in plain language

**Dynamic consent and support**

- Commit to providing updates for evolving AI systems
- Provide multiple accessible channels for questions
- Train research staff to explain AI-related concepts

First, consent documents must provide clear, specific disclosure about the AI system itself. This includes stating the type of AI used (eg, machine learning, deep learning, or natural language processing), its precise role within the trial (eg, diagnostic support, risk stratification, or data processing), and the level of model autonomy—specifically, whether AI outputs are advisory or directly determine clinical actions. Documents should also explain any deviation from standard care attributable to the AI intervention and describe how participants will interact with the technology, if applicable. Such disclosures enable participants to understand not merely that AI is involved, but how it functions within the research context and what that means for their care and data.

Second, risk-benefit communication must be balanced, contextualized, and honest about uncertainty. Risks should be presented with equal prominence to benefits and should encompass clinical risks (eg, misdiagnosis or therapeutic delay), data privacy risks (eg, reidentification or secondary use), and algorithmic risks (eg, bias or performance limitations in underrepresented populations). Benefits must be clearly framed as either potential contributions to science or probable individual gains to avoid therapeutic misconception. Crucially, documents should explicitly acknowledge what remains unknown about the AI system, such as its long-term performance or generalizability, and describe how emerging uncertainties will be monitored and communicated throughout the trial.

Third, readability and accessibility standards are nonnegotiable for genuine comprehension. Consent documents should not exceed 15,000 characters (approximately 10 minutes of reading time) and should achieve a SMOG readability score of 13 or lower, corresponding to a college-level reading demand or below. For trials involving higher AI-related risk, we recommend targeting a 10th-grade reading level or lower. A layered consent structure, featuring a concise summary page with key AI-related information followed by expandable sections for technical details, can accommodate varying levels of participant interest and health literacy. Visual aids, such as flowcharts or icon arrays explaining data flow or decision pathways, should be intentionally designed and evaluated for comprehensibility with representative patient groups. Where feasible, interactive digital consent tools (eg, clickable frequently asked questions or AI-powered chatbots for question answering) may supplement static documents to enhance engagement and understanding.

Fourth, data governance disclosures must be comprehensive and participant centered. Documents should specify what data are collected, how they are stored and secured, who has access, and how long they will be retained. Protocols for data handling after participant withdrawal must be clearly stated, and participants should be offered choices regarding future data use whenever ethically and practically feasible. References to applicable data protection regulations (eg, GDPR and HIPAA) should be presented in plain language, avoiding legal jargon.

Finally, consent should be conceptualized as an ongoing process. For AI systems that evolve during a trial, researchers should commit to providing accessible updates about significant changes, and multiple channels for participant questions should be maintained throughout the study [19,20].

### Limitations

This study, although comprehensive, has several limitations. First, it relies on IC documents uploaded to public registries, which may not represent the final, patient-facing versions used at the point of care. There may be institutional addenda or verbal explanations that supplement these documents, which our methodology could not capture. Second, our analysis focused on the content and readability of the documents themselves and did not assess actual participant comprehension, which is the ultimate measure of consent quality. Third, although our search was comprehensive, it is possible that some relevant trials or IC documents were missed. Finally, the SMOG index, although validated for biomedical texts, is only one measure of readability and may not fully capture all elements of document complexity, such as conceptual density or organizational clarity.

## Appendix

Multimedia Appendix 1 Search strategy and detailed data for included clinical trials.

## Abbreviations

AI: artificial intelligence
GDPR: General Data Protection Regulation
HIPAA: Health Insurance Portability and Accountability Act
IC: informed consent
MRIC-AI: Minimum Requirements for Informed Consent in AI-Related Clinical Trials
SMOG: Simple Measure of Gobbledygook
STROBE: Strengthening the Reporting of Observational Studies in Epidemiology

## Data Availability

The datasets used and/or analyzed during this study are available from the corresponding author on reasonable request.
