# Supplementary material for: Informed Consent Disclosures and Minimum Requirements in AI Clinical Trials: Cross-Sectional Analysis
Source: J Med Internet Res. 2026 Jul 2;28:e94504. doi: 10.2196/94504 (PMC13376845; doi:10.2196/94504)
Supplement: Multimedia Appendix 1 [file jmir_v28i1e94504_app1.docx]

**Supplementary Material**

**Table of contents**

**Supplementary Material 1.** Search strategy and trial selection

**Supplementary Material 2.** Risk Scoring System for Use of AI Models in Clinical Trial

**Supplementary Material 3.** Detailed data of included clinical trial

**Supplementary Material 4.** Detailed data of informed consent

**Supplementary Material 5.** Expert evaluation results

**Supplementary Material 1.** Search strategy and trial selection

**Search strategy**

Databases: Clinicaltrials.gov

Date Run: 9/20/2025

**Clinicaltrial.gov**

| No. | Query | Results |
| --- | --- | --- |
| #1 Artificial intelligence | ("Artificial Intelligence*" OR "AI" OR "Machine Learning*" OR "Deep Learning*" OR "Neural Network*" OR "Natural Language Processing*" OR "NLP" OR "Reinforcement Learning*" OR "Computer Vision*" OR "Predictive Modeling" OR "Predictive Model*" OR "Data Mining*" OR "Pattern Recognition*" OR "Expert System*" OR "Cognitive Computing*" OR "Generative AI" OR "GenAI" OR "Large Language Model*" OR "LLM" OR "Foundation Model*" OR "Transfer Learning*" OR "Supervised Learning*" OR "Unsupervised Learning*" OR "Semi-supervised Learning*" OR "Self-supervised Learning*" OR "Explainable AI" OR "XAI" OR "AI Ethics" OR "Autonomous System*" OR "Intelligent Agent*" OR "Robot* Learning" OR "Speech Recognition*" OR "Image Recognition*" OR "Facial Recognition*" OR "AI in Medicine" OR "AI in Healthcare" OR "Clinical Decision Support*" OR "Automated Diagnosis*" OR "AI Algorithm*" OR "Neural Architecture*" OR "Transformer Model*" OR "Chatbot*" OR "Virtual Assistant*") | 9,918 |
| #2 Informed consent | #1 AND study document: Informed Consent Forms | 311 |

**Trial selection process**


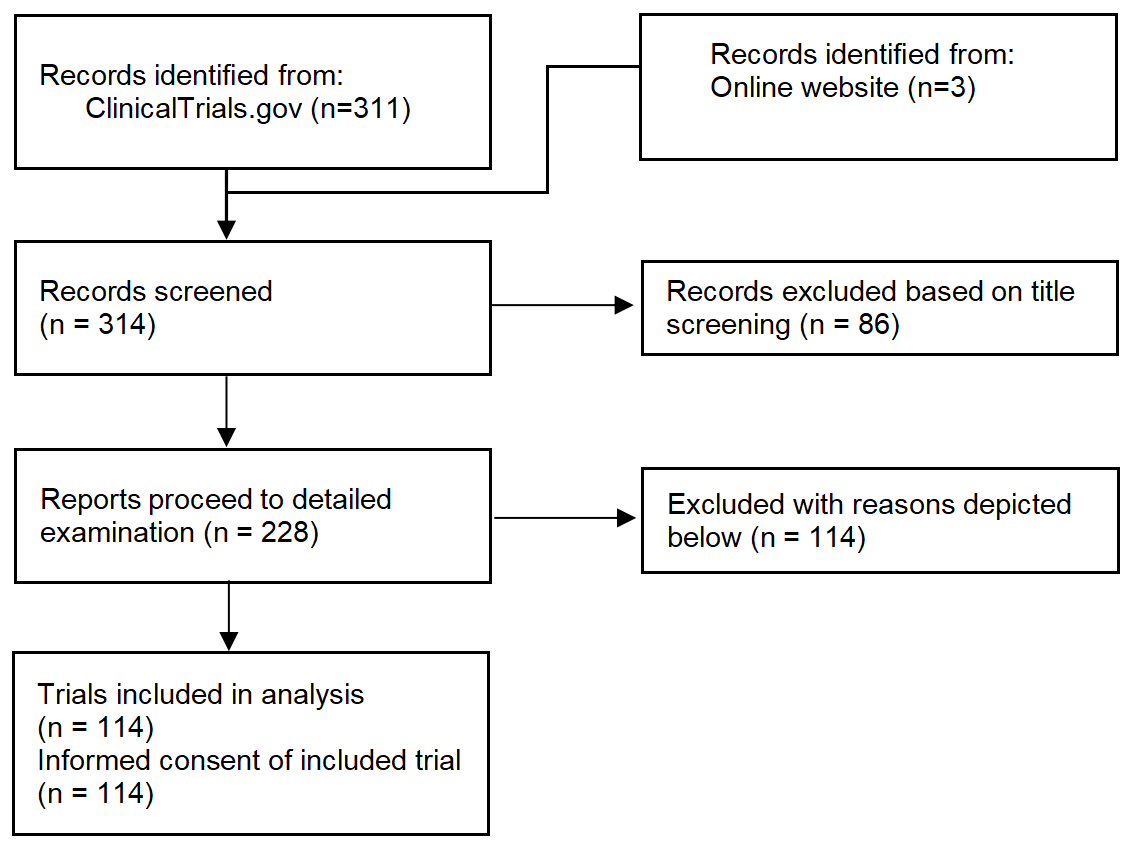


**Supplementary figure 1.** Trial selection process.

**Reasons for trial exclusion after detailed examination**

| **Trial number** | **Exclusion reasons** |
| --- | --- |
| NCT05320406 | not related to AI |
| NCT03153371 | not related to AI |
| NCT04407325 | not related to AI |
| NCT06339190 | not related to AI |
| NCT07127887 | AI is not used to patient |
| NCT03205709 | not related to AI |
| NCT02786472 | not related to AI |
| NCT03407729 | not related to AI |
| NCT06860945 | not related to AI |
| NCT06187077 | not related to AI |
| NCT05263791 | not related to AI |
| NCT06499831 | not related to AI |
| NCT04219670 | not related to AI |
| NCT02813889 | not related to AI |
| NCT05956210 | not related to AI |
| NCT05481138 | not related to AI |
| NCT05455905 | not related to AI |
| NCT05063552 | not related to AI |
| NCT05008640 | not related to AI |
| NCT07076329 | not related to AI |
| NCT07174206 | not related to AI |
| NCT02969707 | not related to AI |
| NCT07138599 | not related to AI |
| NCT06134180 | not related to AI |
| NCT04535414 | not related to AI |
| NCT03105700 | not related to AI |
| NCT04620915 | not related to AI |
| NCT01984710 | not related to AI |
| NCT04252170 | not related to AI |
| NCT06773416 | not related to AI |
| NCT05556772 | not related to AI |
| NCT04005183 | not related to AI |
| NCT02657993 | not related to AI |
| NCT05178381 | not related to AI |
| NCT04043234 | not related to AI |
| NCT03253627 | not related to AI |
| NCT05023252 | not related to AI |
| NCT05516108 | not related to AI |
| NCT04536701 | not related to AI |
| NCT05046886 | not related to AI |
| NCT05447910 | not related to AI |
| NCT05511467 | not related to AI |
| NCT07129889 | not related to AI |
| NCT04348591 | not related to AI |
| NCT07020546 | not related to AI |
| NCT05746195 | not related to AI |
| NCT05806190 | not related to AI |
| NCT03930342 | not related to AI |
| NCT05125133 | not related to AI |
| NCT06743477 | not related to AI |
| NCT05530603 | not related to AI |
| NCT05326438 | not related to AI |
| NCT02290938 | not related to AI |
| NCT04615819 | not related to AI |
| NCT05824754 | not related to AI |
| NCT04617938 | not related to AI |
| NCT06624124 | not related to AI |
| NCT05267990 | not related to AI |
| NCT04866303 | not related to AI |
| NCT03844282 | not related to AI |
| NCT02824263 | not related to AI |
| NCT03694418 | not related to AI |
| NCT03592368 | not related to AI |
| NCT02577835 | not related to AI |
| NCT04948450 | not related to AI |
| NCT05342701 | not related to AI |
| NCT07137741 | not related to AI |
| NCT05413265 | not related to AI |
| NCT07080840 | not related to AI |
| NCT03314688 | not related to AI |
| NCT04018092 | not related to AI |
| NCT04218604 | not related to AI |
| NCT04029298 | not related to AI |
| NCT05178121 | not related to AI |
| NCT06781307 | not related to AI |
| NCT06827873 | not related to AI |
| NCT05987384 | not related to AI |
| NCT04126603 | not related to AI |
| NCT03883867 | not related to AI |
| NCT03651076 | not related to AI |
| NCT06929455 | not related to AI |
| NCT05998005 | not related to AI |
| NCT05937243 | not related to AI |
| NCT02467478 | not related to AI |
| NCT03645941 | not related to AI |
| NCT04294225 | not related to AI |
| NCT04512690 | not related to AI |
| NCT03705169 | not related to AI |
| NCT04972786 | not related to AI |
| NCT03623698 | not related to AI |
| NCT03905863 | not related to AI |
| NCT04583774 | not related to AI |
| NCT04294264 | not related to AI |
| NCT03872856 | not related to AI |
| NCT02646982 | not related to AI |
| NCT02160730 | not related to AI |
| NCT05360888 | not related to AI |
| NCT06487143 | not related to AI |
| NCT05087823 | not related to AI |
| NCT03948295 | not related to AI |
| NCT03944954 | not related to AI |
| NCT06856668 | not related to AI |
| NCT03535974 | not related to AI |
| NCT04522336 | not related to AI |
| NCT03563690 | not related to AI |
| NCT02562716 | not related to AI |
| NCT02739789 | not related to AI |
| NCT06001346 | not related to AI |
| NCT04481204 | not related to AI |
| NCT02730546 | not related to AI |
| NCT04164069 | not related to AI |
| NCT04761614 | not related to AI |
| NCT00217737 | not related to AI |

**Supplementary Material 2.** Risk Scoring System for Use of AI Models in Clinical Trial

| **Risk criteria^a^** | **Level 1^b^** | **Level 2** | **Level 3** |
| --- | --- | --- | --- |
| Model Autonomy | The AI is used solely for data processing, collection, and statistical analysis, with minimal involvement in clinical decision-making. | The AI offers novel suggestions or insights, but the final decision rests entirely with the physician. | The AI plays a highly active role; its recommendations are substantial enough that the physician cannot make a final decision alone and must consult with other clinicians or the responsible authority. |
| Departure from standard of care | No deviation from standard treatment guidelines. | Acceptance of the AI-proposed treatment plan without deviating from standard guidelines. | The AI-proposed treatment plan diverges from both standard clinical guidelines and typical physician decision-making. |
| Patients facing AI | Provides only basic advice with minimal interaction with the patient. | Engages the patient in interaction and offers relevant recommendations. | Demonstrates strong human-machine interactivity, delivering personalized medical advice tailored to the patient’s specific needs. |
| Clinical risk | Even if invasive, artificial intelligence is based on existing procedures to enhance diagnostic accuracy without introducing additional risk of harm. | Uses artificial intelligence to assist in decision-making and provide appropriate treatment, but may contain inaccuracies or lack personalization. | Invasive to the human body, with a potential risk of harm. |

**Note:** ^a^Risk criteria and risk level is designed based on published articles; ^b^The higher the level, the higher the risk of AI.

**Supplementary material 3.** Detailed data of included clinical trial

| **NCT Number** | **Title** | **Year First Posted** | **PI** | **Sponsor** | **Country** | **Status** | **Phase** | **Enrollment** | **Medical speciality** | **Study Type** | **Use of commercially available AI** | **Kind of AI been used** | **Purpose of using AI** | **With the supervision of doctor or not** | **Model autonomy** | **Departure from standards of care** | **Patient-facing AI** | **Clinical risk** | **Primary outcome** | **Mention of informed consent in inclusion or exclusion criteria** | **Additional description on data privacy** |
| --- | --- | --- | --- | --- | --- | --- | --- | --- | --- | --- | --- | --- | --- | --- | --- | --- | --- | --- | --- | --- | --- |
| **NCT02176226** | IntelliCare: Artificial Intelligence in a Mobile Intervention for Depression and Anxiety (AIM) | 2014-06-27 | David C Mohr, Ph.D | Northwestern University | United States | Completed | NA | 105 | Psychiatry | Interventional | IntelliCare | Machine learning | tailor treatment of anxiety and depression | No | 2 | 2 | 2 | 2 | Patient Health Questionnaire - 9(PHQ-9),Generalized Anxiety Disorder Scale-7(GAD-7) | Yes | Data collected will also be used to develop and evaluate machine learning methods which will be used in the subsequent AIM trials |
| **NCT02801877** | IntelliCare Study: Artificial Intelligence in a Mobile (AIM) Intervention for Depression (AIM) | 2016-06-16 | David C Mohr, Ph.D | Northwestern University | United States | Completed | NA | 301 | Psychiatry | Interventional | IntelliCare Hub App | Machine learning | tailor treatment of anxiety and depression | Yes | 2 | 2 | 2 | 2 | Adherence to the Mobile Application Intervention, Patient Health Questionnaire - 9(PHQ-9),Generalized Anxiety Disorder Scale-7(GAD-7) | Yes | Data collected will also be used to further develop and evaluate machine learning methods for future research and deployment efforts. |
| **NCT03685240** | Fall Detection and Prevention for Memory Care Through Real-time Artificial Intelligence Applied to Video: A Randomized Control Trial | 2018-09-26 | NA | SafelyYou | United States | Unknown status | NA | 460 | Geriatrics | Interventional | Not mentioned | AI-enabled cameras, Human in the Loop (HIL) | detect fall related events and upload video only when these events are detected | No | 1 | 1 | 1 | 1 | Enrollment rate, Fall rate due to sit to stand transition detection, Fall rate due to gait change detection | No | NA |
| **NCT03833804** | Data-driven Strategies for Substance Misuse Identification in Hospitalized Patients | 2019-02-07 | NA | University of Wisconsin, Madison | United States | Completed | NA | 64559 | Psychiatry | Interventional | Not mentioned | Machine learning, Natural language processing algorithm | provide a solution to analyze text data in the EHR to identify substance misuse. | No | 1 | 1 | 1 | 1 | Proportion of patients that had a universal screen positive and received SBIRT (screening, brief intervention, or referral to treatment) | No | NA |
| **NCT04442607** | Clinical vAliDation of ARTificial Intelligence in POlyp Detection | 2020-06-22 | Raf Bisschops, MD,PhD | Universitaire Ziekenhuizen KU Leuven | Belgium | Completed | NA | 856 | Gastroenterology | Interventional | Not mentioned | novel state-of-the-art computer-aided detection (CADe) tool | have a beneficial outcome for the patient, a better polyp detection | Yes | 2 | 2 | 1 | 1 | otal polyp detection during single pass colonoscopy by the artificial intelligence tool in comparison to polyp detection by the endoscopist with endoscopic diagnosis as a gold standard | Yes | NA |
| **NCT04444908** | Development and Validation of an Artificial Intelligence-assisted Strategy Selection System for Colonoscopy Cleaning | 2020-06-24 | Honggang Yu, Doctor | Renmin Hospital of Wuhan University | China | Unknown status | NA | 657 | Gastroenterology | Observational | Endoangel | Deep learning | develop the EndoAngel with real-time intestinal cleanliness assessment | Yes | 2 | 2 | 1 | 1 | The adenoma detection rate (ADR), Cleanliness assessment of different intestinal segment in the artificial intelligence system | Yes | NA |
| **NCT04458220** | Research on Early Warning and Solution System of Difficult Airway in Perioperative Period Based on Artificial Intelligence | 2020-07-07 | NA | Shanghai Ninth People's Hospital Affiliated to Shanghai Jiao Tong University | China | Unknown status | NA | 16000 | Anesthesiology | Observational | Not mentioned | Deep learning and intelligent analysis | Several of the most relevant landmarks will be selected to build an early warning model | Yes | 1 | 1 | 1 | 1 | Diagnosed as a difficult airway | Yes | NA |
| **NCT04749277** | Artificial Intelligence in the Characterization of Small and Diminutive Colorectal Polyps: A Prospective Study in a Clinical Setting Using CAD EYE® | 2021-02-11 | Elisa Gravito-Soares, MD | Centro Hospitalar e Universitário de Coimbra, E.P.E. | Portugal | Completed | NA | 197 | Gastroenterology | Observational | CAD EYE® | Deep learning | evaluate the diagnostic accuracy of computer-aided diagnosis | Yes | 2 | 2 | 1 | 1 | Real-time optical characterization of colorectal polyps (CAD EYE® versus Histopathology), | No | NA |
| **NCT04761211** | Effect and Safety of Smart Bra of Peking Union Medical College & Hospital | 2021-02-18 | NA | Peking Union Medical College Hospital | China | Completed | NA | 2141 | Gynecology | Interventional | Not mentioned | Not mentioned | breast disease screening in outpatients with breast surgery | No | 1 | 1 | 1 | 1 | Sensitivity | Yes | NA |
| **NCT04892329** | A Multi-center Study on the Efficacy and Safety of Artificial Intelligence-assisted Navigation System for Biliopancreatic Endoscopic Ultrasonography | 2021-05-19 | Honggang Yu, Doctor | Renmin Hospital of Wuhan University | China | Unknown status | NA | 285 | Oncology | Interventional | Endoangel | Deep learning | improve the quality of EUS and reduce the missed diagnosis of pancreatic lesions | Yes | 2 | 2 | 1 | 1 | Missed scanning rate of adjacent important anatomical structures in pancreatic endoscopic ultrasonography | Yes | NA |
| **NCT04903444** | A Single-center Study on the Effectiveness and Safety of Artificial Intelligence Assisted System in Clinical Application of Endoscopic Retrograde Cholangiopancreatography | 2021-05-26 | Honggang Yu, Doctor | Renmin Hospital of Wuhan University | China | Unknown status | NA | 62 | Oncology | Interventional | Not mentioned | Deep learning | instruct the direction of guide wire and the position of stent placement in real time. | Yes | 2 | 2 | 1 | 1 | Procedure time | Yes | NA |
| **NCT04912037** | A Study on the Effectiveness of Artificial Intelligence-assisted Colonoscopy in Improving the Effect of Colonoscopy Training for Trainees | 2021-06-03 | Yu w Honggang, Doctor | Renmin Hospital of Wuhan University | China | Unknown status | NA | 385 | Gastroenterology | Interventional | Endoangel | Deep learning | improve the colonoscopy performance of novice physicians and assist the colonoscopy training | Yes | 2 | 2 | 1 | 1 | CUSUM learning curve for colonoscopy (ACE scoring scale), Average test score difference before and after training | Yes | NA |
| **NCT05287477** | Passive Evaluation in Operational Environment of the AI Clinician Decision Support System for Sepsis Treatment | 2022-03-18 | NA | Imperial College London | United Kingdom | Recruiting | NA | 15 | Critical Care Medicine | Observational | Not mentioned | Reinforcement learning | capable of processing patient data within the electronic patient record of NHS hospitals in real-time to suggest a course of action | Yes | 1 | 1 | 1 | 1 | Data Availability, Anonymised patients' data, Rate of intravenous fluids administered to patients, Evaluators' data (the doctors assessing the AI in the background), System Availability | No | NA |
| **NCT05311046** | Biomarker-enhanced Artificial Intelligence Based Pediatric Sepsis Screening Tool Towards Early Recognition and Personalized Therapeutics | 2022-04-05 | NA | Computer Technology Associates, Inc | United States | Active, not recruiting | NA | 12961 | Pediatric, Emergency departments | Observational | Not mentioned | Machine learning | used in combination with the hospital's electronic health record (EHR) system to monitor and assess real-time emergency department (ED) electronic health record (EHR) data towards the enhancement of early pediatric sepsis recognition and the initiation of timely, aggressive personalized sepsis therapy known to improve patient outcomes | No | 1 | 1 | 1 | 1 | Effective Expert System-based Pediatric Sepsis Screening Tool (PSCT), High performance Expert System-based Pediatric Sepsis Screening Tool (PSCT) | No | NA |
| **NCT05335889** | Feasibility of Using Wearable Sensors and Artificial Intelligence for Carbohydrate Counting in Chinese Americans With Type 2 Diabetes | 2022-04-20 | Yaguang Zheng, PhD, RN | NYU Langone Health | United States | Completed | NA | 12 | Endocrinology | Observational | eButton | Not mentioned | determine food names, volumes, and nutrient value of the consumed food | Yes | 1 | 1 | 2 | 1 | Accuracy of Carbohydrate Counting using eButton (absolute error, relative error), Proportion of participants who are fully compliant with eButton use | No | NA |
| **NCT05352399** | Artificial Intelligence + Care Coach Intervention for Persons Living With Dementia and Caregivers | 2022-04-28 | Cameron Gettel | Yale University | United States | Completed | NA | 40 | Gynecology | Interventional | NeuViCare AI services | Not mentioned | enables review of ordered tests and external resources recommended by the care provider, provides context-sensitive, personalized text-based assistance to help patients' complete care plan captured in the NeuViCare Planner | No | 1 | 1 | 2 | 1 | Intervention Appropriateness Measure, Feasibility of Intervention Measure, Acceptability of Intervention Measure, System Usability Scale | No | NA |
| **NCT05381064** | Effect of a Deep Learning-based Bile Duct Scanning System on the Diagnostic Accuracy of Common Bile Duct Stones During Examination by Novice Ultrasound Endoscopists: a Single-center, Tandem, Randomized Controlled Trial | 2022-05-19 | Honggang Yu, Doctor | Renmin Hospital of Wuhan University | China | Unknown status | NA | 184 | Gastroenterology | Interventional | Not mentioned | Deep learning | improving the diagnostic accuracy of common bile duct stones and reducing the rate of missed gallstones during bile duct scanning | Yes | 2 | 2 | 1 | 1 | Accuracy of diagnosis of common bile duct stones in patients with low and intermediate risk by novice combined with AI-assisted and expert | Yes | NA |
| **NCT05437237** | Algorithm Development Through Artificial Intelligence for the Triage of Stroke Patients in the Ambulance With Electroencephalography | 2022-06-29 | Jonathan M Coutinho, MD, PhD | Academisch Medisch Centrum - Universiteit van Amsterdam (AMC-UvA) | Netherlands | Recruiting | NA | 1192 | Vasculary surgery | Interventional | Not mentioned | AI based electroencephalography (EEG) algorithms (the AI-STROKE algorithms) | maximal diagnostic accuracy to identify patients with an large vessel occlusion of the anterior circulation (LVO-a) in a population of patients with suspected acute ischemic stroke | Yes | 2 | 2 | 1 | 1 | One or more novel AI-based EEG algorithms based on dry electrode EEG-data with optimal diagnostic accuracy for LVO-a | Yes | NA |
| **NCT05534178** | Machine Learning Model to Predict Hospital Length of Stay (HOLS) and Mortality After Discharge in Hospitalized Oncologic Patients [Plantology Database]: a Multicenter Cross-validation Study | 2022-09-09 | Oriol Mirallas, MD | Vall d'Hebron Institute of Oncology | Spain | Unknown status | NA | 2500 | Oncology | Observational | Not mentioned | Machine learning | develop a predictive tool at admission to help physicians adjust medical interventions and detect possible actions that will need to be implemented during hospitalization in order to improve the overall survival and quality of life of our patients | No | 2 | 2 | 1 | 1 | Predict Mortality, hospital length of stay | No | NA |
| **NCT05537922** | I3LUNG: Integrative Science, Intelligent Data Platform for Individualized LUNG Cancer Care With Immunotherapy | 2022-09-13 | NA | Fondazione IRCCS Istituto Nazionale dei Tumori, Milano | United States, Greece, Israel, spain | Recruiting | NA | 2200 | Oncology | Observational | Not mentioned | Machine learning | create a first version of the PDSS tool, an AI-based tool to provide an easy and ready-to-use access to predictive models, increasing care appropriateness, reducing the negative impacts of prolonged and toxic treatments on wellbeing and healthcare costs. | No | 2 | 2 | 1 | 1 | Response Rate | Yes | NA |
| **NCT05558605** | Use of Artificial Intelligence-Guided Echocardiography to assIst cardiovascuLar Patient managEment | 2022-09-28 | Tom Marwick, MBBS, PhD | Baker Heart and Diabetes Institute | Australia | Recruiting | NA | 612 | Cardiology | Interventional | Caption Health, Brisbane, CA | AI-guided echocardiography | triage and management of patients with known or suspected heart disease in RRA | Yes | 2 | 2 | 1 | 1 | Diagnosis of cardiac dysfunction or heart valve disease | No | NA |
| **NCT05619042** | Detection of Coronary Artery Calcifications by Whole Blood Transcriptome Analyzed by Artificial InTelligence Algorithms. (CAC-TRAIT Study) | 2022-11-16 | Santiago G Miriuka, MD MSc PhD | Santiago Gabriel Miriuka | Argentina | Completed | NA | 800 | Cardiology | Observational | Not mentioned | Artificial InTelligence Algorithms (CAC-TRAIT) | detect the presence and extent of coronary calcification in individuals without a history of known cardiovascular disease | No | 2 | 2 | 1 | 1 | Coronary artery calcium score | Yes | NA |
| **NCT05708846** | Observational Study for the Improvement of a Digital Health Platform for Remote Monitoring of Patients With Heart Failure | 2023-02-01 | Julio César MD Blázquez | humanITcare | Romania, Spain | Completed | NA | 154 | Cardiology | Observational | Vitalera | Machine learning | improve the remote monitoring system and its alarm-based system by making it more robust, trustworthy and reliable | No | 1 | 1 | 1 | 1 | Number of Patients Included in the Dataset, Implement ML Models to Improve the Current Alarm-based System Using the Dataset Created | Yes | NA |
| **NCT05754476** | Multi-center and Prospective Cohort Study of Artificial Intelligence Model for Gadolinium-based Contrast Agent Reduction in Brain MRI (MAGNET) | 2023-03-03 | Yaou Liu, PhD | Beijing Tiantan Hospital | China | Unknown status | NA | 3000 | Radiology, Neurology | Observational | Not mentioned | Deep learning | process pre-contrast images and/or low-dose T1 images to predict virtual contrast-enhanced T1 (vir-T1c) images, taking the full-dose images as the reference standard | Yes | 1 | 1 | 1 | 1 | quantitative metrics, qualitative assessments | Yes | NA |
| **NCT05843682** | Technological Innovation in the Virtual Assistance of Patients With Uncontrolled Arterial Hypertension - Hyper 2 | 2023-05-06 | NA | University of Sao Paulo General Hospital | Brazil | Unknown status | NA | 100 | Cardiology | Interventional | Avatr | Not mentioned | monitor their health data, as well as provide individualized care and early intervention through alerts generated for the multidisciplinary team | Yes | 1 | 1 | 1 | 1 | Blood pressure control,Glycemic control, Change of out-of-hospital visits | No | NA |
| **NCT05872945** | Development and Implementation of Model-based Systems for Professional Football Teams, Aimed at Optimizing Health and Performance | 2023-05-24 | Adolfo Munoz Macho, Dr | RCD Mallorca SAD | Spain | Completed | NA | 54 | Sports Medicine | Observational | Not mentioned | Machine learning | obtain models and results in the interpretation of physical, biomedical and physiological parameters of the players | No | 2 | 2 | 2 | 1 | Waves Detection | No | NA |
| **NCT05967260** | A Crossover Study to Assess the Effect of an Artificial Intelligence (AI)-Based Bedtime Smart Snack Intervention in Preventing Overnight Low Glucose in People With T1D on Multiple Daily Injections. | 2023-08-01 | NA | Oregon Health and Science University | United States | Completed | NA | 21 | Endocrinology | Interventional | DailyDose Smart Snack app | Not mentioned | predict the likelihood of overnight low glucose at bedtime and will recommend a personalized snack to help avoid nocturnal hypoglycemia | No | 2 | 1 | 2 | 1 | Probability of Overnight Hypoglycemia | Yes | NA |
| **NCT06017089** | The Pediatric Artificial Pancreas Automated Initialization Trial (PEDAP-AI): A Pilot Study of AI Advisor-Driven Pump Initiation and Parameter Adaptation in Young Children With Type 1 Diabetes | 2023-08-30 | Raj Paul Wadwa, MD | Marc Breton | United States | Completed | NA | 33 | Endocrinology | Interventional | t:slim X2 with Control-IQ Technology | AI-based Advisor system | adverse events related to hypoglycemia and hyperglycemia, CGM-measured time spent below 54mg/dL, and CGM-measured time spent above 250 mg/dL. | Yes | 1 | 1 | 1 | 1 | Percentage below 54mg/dL, above 250 mg/dl, Hierarchical Efficacy Endpoints (tested for superiority compared with baseline) CGM Measured | No | NA |
| **NCT06240234** | Artificial Intelligence - to Predict and Prevent Hypotension During Surgery | 2024-02-02 | Greg Winski, Dr | Region Stockholm | Sweden | Not yet recruiting | NA | 300 | Anesthesiology | Interventional | Not mentioned | Machine learning | train AI algorithms with the goal of constructing Effective and accurate methods for predicting low blood pressure preventively. | Yes | 1 | 1 | 1 | 1 | Accuracy of prediction of hypotension 5 minutes ahead of time using capnodynamics and continuous arterial blood pressure measurement. | Yes | NA |
| **NCT06240897** | Exploring the Feasibility of Digital Intervention Games as a Diagnostic and Management Tool for Delirium at SGH Inpatient Wards | 2024-02-05 | Dean Ho, PhD, Esther M Fan Peijin | Institute for Digital Medicine (WisDM) | Singapore | Not yet recruiting | NA | 20 | Psychiatry | Interventional | CURATE.DTx | Not mentioned | dynamically personalise cognitive training by modifying the game intensity | No | 2 | 2 | 2 | 1 | Patient Acceptability, Trial Team Members Acceptability, Demand, Implementation, Practicality | Yes | NA |
| **NCT06253065** | Prospective Validation of Pathology-based Artificial Intelligence Diagnostic Model for Lymph Node Metastasis in Prostate Cancer | 2024-02-12 | NA | Sun Yat-Sen Memorial Hospital of Sun Yat-Sen University | China | Recruiting | NA | 100 | Oncology | Observational | Not mentioned | Deep learning | detecting pathological lymph node metastasis (LNM) of prostate cancer | Yes | 2 | 2 | 1 | 1 | sensitivity | Yes | NA |
| **NCT06421324** | Interventional Study Focused on Providing Personalised Health Recommendations to the General Population Through an Integrated AI Guided App as a Strategy for Gastric Cancer Prevention (AIDA) | 2024-05-20 | Tania Fleitas, MD, PhD | Fundación para la Investigación del Hospital Clínico de Valencia | Spain | Recruiting | NA | 450 | Oncology | Interventional | Not mentioned | Not mentioned | helps clinicians diagnose precancerous inflammation, suggests personalised therapeutic strategies for medical treatment and follow-up, and makes personalised recommendations for monitoring patient health status, thus contributing to gastric cancer prevention | No | 2 | 2 | 2 | 1 | Risk of developing Gastric Cancer based on medical records, H. pylori Eradication Therapy Recommendation, GIM risk score assessment using imaging modalities | Yes | NA |
| **NCT06357039** | Validation Study of an Artificial Intelligence-based Sleep Stage Classification for a Home Sleep Tracking Device | 2024-04-10 | NA | PNAPS Health Informatics and Space Technologies Inc. | Turkey | Completed | NA | 305 | Neurology | Observational | Not mentioned | Convolutional Neural Networks model (CNN) | extracting features for each epoch window independently from before and after sleep onset (epoch encoder), and then trained in the context of long-term relationships in the sleep process (sequence encoder), | No | 1 | 1 | 2 | 1 | Sleep Stages Classification Accuracy, Interoception analysis from PPG data collected from facial skin | No | NA |
| **NCT06435286** | Effectiveness and Performance of a Mobile, Automated, Optical Biopsy Technology for Esophageal Cancer Screening: A Clinical Study in Brazil and the United States | 2024-05-30 | Sharmila Anandasabapathy, MD | Baylor College of Medicine | United States, Brazil | Recruiting | II | 200 | Oncology | Interventional | Microendoscope (AI-mHRME) imaging | Deep learning | aid in the detection of neoplastic images and determine the performance, efficiency, and impact of the AI-mHRME when to Lugol's chromoendoscopy (LCE) alone and when using AI-mHRME with LCE | Yes | 2 | 2 | 1 | 1 | Clinical Impact, Performance Characteristics, Procedure Efficiency, Clinician Confidence | Yes | NA |
| **NCT06455111** | Evaluation of an Artificial Intelligence-Assisted, Image-Based Dietary Assessment Tool in the Framingham Heart Study: A Block Randomized Controlled Trial | 2024-06-12 | Boston University | Boston University | United States | Recruiting | NA | 115 | Cardiology, Nutrition | Interventional | Keenoa | enhanced, image-assisted tool | capture real-time food intake may aid in overcoming limitations of existing methods | Yes | 1 | 1 | 1 | 1 | Acceptability of Dietary Assessment Method, Completion of 3 Day Dietary Assessment With >= 600 kcal | No | NA |
| **NCT06474338** | Artificial Intelligence Detection of Bladder Tumors Under Endoscopy | 2024-06-25 | Zixing Ye | Peking Union Medical College Hospital | China | Recruiting | NA | 1000 | Oncology, Urology | Observational | Not mentioned | HRNet algorithm/ Convolutional neural networks (CNNs) | detect bladder tumors better than urologists under cystoscopy | No | 2 | 2 | 1 | 1 | intersection over union | No | Data Management and Confidentiality All data in this study is properly stored to ensure security without loss or leakage. Sensitive information and patient information will not be uploaded to public platforms. During data processing, patients' personal information will be anonymized, and patient identification codes will be used to replace patient names and IDs. If technical services are needed, data will be appropriately encrypted, and a confidentiality agreement will be signed. |
| **NCT06644248** | Development and Evaluation of an Artificial Intelligence Model for Cervical Cancer Detection From Colposcopic Images | 2024-10-16 | Taufiq Hasan, Taufiq | Bangladesh University of Engineering and Technology | Bangladesh | Recruiting | NA | 500 | Oncology | Observational | Not mentioned | Deep learning | enhance the accuracy and efficiency of cervical cancer detection, particularly in low-resource settings | No | 2 | 2 | 1 | 1 | Swede Score Evaluation | No | NA |
| **NCT06652061** | Development and Evaluation of an Artificial Intelligence Model for Bone Mineral Density Prediction From X-Ray Images | 2024-10-22 | Taufiq Hasan, PhD | Bangladesh University of Engineering and Technology | Bangladesh | Recruiting | NA | 600 | Orthopedics | Observational | Not mentioned | Deep learning | predicting Bone Mineral Density (BMD) from X-ray images using deep learning techniques, with a particular focus on improving the model's generalizability across diverse populations | No | 2 | 2 | 1 | 1 | Bone Mineral Density (BMD) - Hip and Spinal (L1-L4) | Yes | NA |
| **NCT06652854** | Implementation of Large Language Models in Anesthesia to Answer Patients' Questions During Pre-Anesthesia Visits: A Prospective, Observational Study | 2024-10-22 | NA | Ottawa Hospital Research Institute | Canada | Not yet recruiting | NA | 190 | Anesthesiology | Observational | ChatGPT | Large language model | answering common questions patients have, allowing anesthesiologists to focus their limited time on addressing complex and personalized issues for each patient | no | 2 | 2 | 2 | 2 | knowledge | Yes | The data collected from this study will not be shared with other researchers, however the data may be used to inform and guide future research on the integration of AI technologies in clinical practice, particularly in improving patient communication and decision-making during pre-anesthesia consultations. |
| **NCT06712160** | Diagnostic Accuracy of Artificial Intelligence, CBCT, and Clinical Examination in Detecting Number of Root Canals in Conventional and Retreated Maxillary and Mandibular Molars | 2024-12-02 | NA | Misr International University | Egypt | Completed | NA | 212 | Dentistry | Interventional | Diagnocat | convolutional neural network (CNN) | detecting root canals in upper first, upper second, and lower first molars | No | 1 | 1 | 1 | 1 | The number of canals detected | Yes | NA |
| **NCT06713122** | The Effect of Symptom Management Training Given to Gynaecological Cancer Patients Receiving Chemotherapy With Artificial Intelligence Supported Mobile Application on Supportive Care Needs, Symptom Severity and Psychological Well-Being | 2024-12-03 | Gülten Güvenç, Prof.Dr | Esra Nur Erdoğan | Turkey | Enrolling by invitation | NA | 70 | Gynecology, Oncology | Interventional | Not mentioned | Not mentioned | evaluate the effect of symptom management training given with an artificial intelligence supported mobile application developed for gynaecological cancer patients receiving chemotherapy on supportive care needs, symptom severity and psychological well-being. | Yes | 2 | 2 | 2 | 1 | Supportive Care Needs, Symptom Severity, Psychological Well-Being | No | NA |
| **NCT06717984** | A Study on Artificial Intelligence Algorithms for Breast Cancer Classification From Histopathology Images | 2024-12-05 | Taufiq Hasan, PhD, Farida Arjuman | Taufiq Hasan, PhD | Bangladesh | Recruiting | NA | 500 | Oncology | Observational | Not mentioned | Deep learning | classify invasive and noninvasive breast cancer types from histopathological images | No | 2 | 2 | 1 | 1 | Accuracy of Deep Learning Model in Classifying Breast Tissue as Normal Benign, In Situ, or Invasive. | No | NA |
| **NCT06815939** | Validation of a Lab-free Low-cost Screening Test for Prevention of Cervical Cancer: Automated Visual Evaluation | 2025-02-10 | Karla Alfaro, MD, David Levitz, PhD | DL Analytics | El Salvador | Recruiting | NA | 10000 | Oncology | Observational | Automated Visual Evaluation (AVE), CINFinder version | point-of-care screening and triage diagnostic tool | compare the sensitivity of AVE (CINFinder version) with traditional screening and triage tests | No | 2 | 2 | 1 | 1 | Positive Predictive Value (PPV), Sensitivity | Yes | For storage and management, data from paper forms will be transferred to REDCap, an electronic data management system widely used for clinical research. CervManager and REDCap can be downloaded and merged periodically to easily identify and correct any entry errors, duplications, or missing data on either database. All AVE algorithms implemented as part of this project and used for subsequent analyses will run on DL Analytics servers. |
| **NCT06842927** | DETECT-PD -- Dialysis Efficiency and Transporter Evaluation Computational Tool in Peritoneal Dialysis | 2025-02-24 | NA | Tuen Mun Hospital | China | Enrolling by invitation | NA | 350 | Nephrology | Observational | Not mentioned | Deep learning | predicting peritoneum transporter status and dialysis efficiency in adult patients undergoing peritoneal dialysis | No | 2 | 2 | 1 | 1 | Peritoneal Equilibration Test (PET) Parameters | Yes | NA |
| **NCT06858553** | A Prospective, Multi-center Study to Characterize Intestinal Fibrosis in Patients With Crohn's Disease (CD) Using MR Enterography (MRE)-Based Artificial Intelligence | 2025-03-05 | NA | Minhu Chen | China | Recruiting | NA | 234 | Gastroenterology | Observational | Not mentioned | Deep learning | accurately characterize intestinal fibrosis | Yes | 2 | 2 | 1 | 1 | histologic inflammation score, histologic fibrosis score, Magnetization Transfer Ratio, Apparent Diffusion Coefficient, Percentage of Enhancement Gain | No | Raw data may include sensitive personal information, such as names and medical history. Public release could expose this information, violating patient privacy rights and privacy regulations. |
| **NCT06859216** | Evaluating AI-Generated Plain Language Summaries on Patient Comprehension of Ophthalmology Notes Among English-Speaking Patients | 2025-03-05 | Prashant Tailor, MD | University of California, Los Angeles | United States | Recruiting | NA | 460 | Ophthalmology | Interventional | Not mentioned | Large Language Model | help people understand their eye doctor's notes better | No | 1 | 1 | 2 | 1 | Patient Comprehension Score (Immediate Post-Visit) | Yes | De-identified individual participant data (IPD) underlying the study's results will be shared. The IPD includes patient survey responses on comprehension of ophthalmology visit notes (both immediately post-visit and at 1-week follow-up), patient satisfaction ratings, and demographic information (age, gender, education level, and previous ophthalmology experience). Additionally, survey responses from ophthalmologists regarding the accuracy, clarity, and time efficiency of the AI-generated plain language summaries will be provided. All data will be fully de-identified in compliance with HIPAA and UCLA guidelines using unique study IDs to replace personal identifiers, and no code keys linking data to individual participants will be shared. |
| **NCT06904586** | Evaluation of Anthropometric and Ultrasonographic Measurements With Different Machine Learning Methods in Predicting Difficult Intubation: A Prospective Observational Study | 2025-04-01 | Gizem DEMIR SENOGLU | Duzce University | Turkey | Completed | NA | 329 | Anesthesiology | Observational | Not mentioned | Not mentioned | accurately predict difficult intubation and facilitate early preparation | No | 1 | 1 | 1 | 1 | Support Vector Machine Algorithm Percentage of Accuracy in Predicted Difficult Intubations | No | NA |
| **NCT06989255** | A Multi-center Cohort Study for Conventional Ultrasound Image Set Collection to Create a Training Data Set for Research Purposes (Image Processing and Analysis, AI Model Training). | 2025-05-25 | Andrius Macas, Prof. Dr, Michail Potoupnis, Prof, Elvira Grandone, Prof, Maxime Gautier, Dr, Savvas Defteraios, Prof | ThrombUS+ | France, Greece, Italy, Lithuania | Recruiting | NA | 3000 | Vasculary surgery | Observational | ThrombUS+ | Not mentioned | achieve automated early DVT detection, provide a continuous assessment of DVT risk and support DVT prevention via extended reality and serious gaming | No | 2 | 2 | 1 | 1 | Ultrasound data | Yes | The data set will be completely anonymized to be used for research purposes, in compliance with the General Data Protection Regulation (GDPR) and the European Health Data Space (EHDS) and the upcoming Artificial Intelligence Act (AIA). Furthermore, the anonymized data set will be described in the Argos/OpenAIRE tool and will be made available through the European Open Science Cloud (EOSC) portal via OpenAIRE, to be used by other researcher. |
| **NCT07000721** | Research on New Intelligent Diagnosis and Treatment Technologies for Early Lung Cancer Based on Multimodal Imaging Bronchoscopy Navigation | 2025-06-03 | NA | Jisong Zhang | China | Completed | NA | 92 | Respiratory, Oncology | Interventional | SARS-pro | Deep learning | realize accurate construction of small airways and guide accurate biopsy | No | 2 | 2 | 1 | 1 | Diagnostic positive yield | No | NA |
| **NCT07108452** | Leveraging AI to Transform Dietary Choices for Cardiovascular Health in Young Adults Experiencing Food Insecurity | 2025-08-07 | Lauren E Au, PhD, RDN | University of California, Davis | United States | Not yet recruiting | NA | 114 | Cardiology | Interventional | Not mentioned | Not mentioned | assessing dietary intake | No | 1 | 1 | 1 | 1 | App acceptability by version assigned to participants, App's usability by version assigned to participants, App adherence rates by version assigned to participants | No | NA |
| **NCT04473326** | Optimizing Message Framing for Healthy Habits for Patients With Type 2 Diabetes - Phase II (Pragmatic Trial) | 2020/7/16 | NA | Brigham and Women's Hospital | United States | Completed | NA | 60 | Endocrinology | Interventional | Not mentioned | Reinforcement Learning | enhanced text messaging program to support medication adherence in patients with type 2 diabetes. | No | 1 | 1 | 1 | 1 | Medication Adherence | No | NA |
| **NCT05369806** | Leveraging Interactive Text Messaging to Monitor and Support Maternal Health in Kenya | 2022/5/11 | Keshet Ronen, PhD | University of Washington | Kenya | Completed | NA | 80 | Gynecology | Interventional | Mobile WACh system (AI-NEO) | Natural Language Processing | use of a natural language processing computer algorithm on incoming SMS messages with pregnant people and new mothers in Kenya to see if it can help to identify urgent messages. | No | 1 | 1 | 1 | 1 | Acceptability, Nurse Response Time | No | Data from AI-NEO will be available at end of the project by contacting the study team at the University of Washington (keshet@uw.edu). |
| **NCT07136207** | Research on Delirium Recognition in Neurocritical Patients Based on Facial Expression Behavior Patterns | 2025/8/22 | NA | Beijing Tiantan Hospital | China | Recruiting | NA | 1000 | Neurology | Observational | Not mentioned | Machine learning, convolutional neural network (CNN) | image processing, and pattern recognition technologies to perform digital analysis of facial expression behaviors in neurocritical care patients with delirium | No | 1 | 1 | 1 | 1 | Accuracy of the delirium prediction model, Sensitivity of the delirium prediction model, Specificity of the delirium prediction model | Yes | This study involves collecting facial information of patients, which pertains to their privacy. To protect participants' confidentiality, all data will be uniformly destroyed after the study is completed. The investigators will not share or disclose patients' information to other researchers. |
| **NCT06008548** | A New Conception About Individualized Treatment Allocation for HCC-Using Machine Learning | 2023/8/23 | NA | Tang-Du Hospital | China | Completed | NA | 4991 | Oncology | Observational | Not mentioned | Machine learning | simulate the survival outcomes of patients allocated to different treatments. | No | 2 | 2 | 1 | 1 | Overall survival | No | NA |
| **NCT07102810** | Standardized Hypnotic Susceptibility Testing to Facilitate Development of a Machine Learning Tool to Characterize Physiological Biomarkers of Calm and Tranced States | 2025/8/5 | David L Reich, MD | Icahn School of Medicine at Mount Sinai | United States | Completed | NA | 50 | Psychiatry | Interventional | Not mentioned | Machine learning | quantifies a patient's instantaneous emotional/arousal state along the spectrum that spans anxiety through states of calmness and trance. | No | 1 | 1 | 1 | 1 | Harvard Group Scale of Hypnotic Susceptibility (HGSHS:A) Total Score | Yes | Aggregate deidentified data and results will be shared. Individual participant video and EEG data will not be shared due to PHI concerns. |
| **NCT04870099** | Leveraging Computational Social Sciences and Natural Language Processing to Optimize Engagement and Response to Low-intensity CBT for Depression and Anxiety | 2021/5/3 | NA | Indiana University | United States | Completed | NA | 141 | Psychiatry | Interventional | Not mentioned | Natural Language Processing | Optimize Engagement and Response to Low-intensity CBT for Depression and Anxiety | No | 1 | 1 | 1 | 1 | 6-week Change in Kessler 6 Psychological Distress Scale (K6), 6-week Change in the WHO 5 Well-being Index (WHO-5) | No | NA |
| **NCT05819151** | Diabetes Screening and Monitoring Using Tongue Images and Self-reported Symptoms: a Machine Learning Approach | 2023/4/19 | Shi Ping Zhang, PhD | Hong Kong Baptist University | Hong Kong | Unknown status | NA | 4000 | Endocrinology | Observational | Not mentioned | Machine learning, Convolutional Neural Networks (CNN) | build an algorithm for HbA1c prediction with reasonable accuracy. | Yes | 2 | 1 | 1 | 1 | Tongue image features | Yes | NA |
| **NCT06400277** | In-Vivo Comparison of Different Impression Methods in Complete Edentulous Upper Jaw | 2024/5/6 | Sinem Kahya Karaca | Hacettepe University | Turkey | Completed | NA | 15 | Dentistry | Interventional | Not mentioned | Not mentioned | digital impression taken | No | 1 | 1 | 1 | 1 | Three Dimensional Difference Between Irreversible Hydrocolloid and Zinc Oxide Eugenol Impressions, Three Dimensional Difference Between Zinc Oxide Eugenol and Intraoral Scanner AI Off Impressions, Three Dimensional Difference Between Intraoral Scanner AI Off and AI On Impressions, Three Dimensional Difference Between Intraoral Scanner AI Off and Modified Impressions | No | NA |
| **NCT07112599** | Clinical Study on Predicting Lymph Node Metastasis of High-risk Prostate Cancer Based on Artificial Intelligence Multi-omics Analysis：A Multicenter, Prospective and Observational Clinical Study | 2025/8/8 | NA | Anhui Medical University | China | Not yet recruiting | NA | 2000 | Oncology | Observational | Not mentioned | Not mentioned | determines whether the patient has lymph node metastasis based on the MRI results and the pathological section image information of the case combined with clinical data before radical resection of the prostate. | Yes | 2 | 1 | 1 | 1 | The area under curve (AUC) of Receiver Operating Characteristic (ROC) curves of the radiopathology artificial intelligence model | Yes | NA |
| **NCT04378660** | Artificial Intelligence Validation Trial for Polyp Detection: Pilot Study | 2020/5/7 | Raf Bisschops | Universitaire Ziekenhuizen KU Leuven | Belgium | Completed | NA | 357 | Gastroenterology | Interventional | Not mentioned | Not mentioned | automated endoscopic tool as second observer during routine diagnostic colonoscopy | Yes | 2 | 2 | 1 | 1 | Diagnostic accuracy of the novel AI system compared to endoscopic diagnosis as gold standard | Yes | We don't share any patient information with other researchers, nor when it's anonymized |
| **NCT05671601** | Application of Deep Learning Automation Based on Time-lapse Imaging to Jointly Assess Embryo Development to Improve Pregnancy Outcome of Single Blastocyst Transfer | 2023/1/4 | NA | The Affiliated Nanjing Drum Tower Hospital of Nanjing University Medical School | No location data | Unknown status | NA | 100 | Reproductive medicine | Observational | Not mentioned | Deep Learning | apply the deep learning automation based on Time-lapse imaging to jointly assess embryo development，so that it can ensure the consistency of embryo evaluation and improve the accuracy of evaluation | Yes | 3 | 2 | 1 | 1 | Implantation rate | No | NA |
| **NCT03482466** | A Virtual World/Neurofeedback Real Time Functional MRI Approach to PTSD Treatment | 2018/3/29 | Ramiro Salas, PhD,Michael E. DeBakey | VA Office of Research and Development | United States | Terminated | NA | 14 | psychiatry | Interventional | Not mentioned | Machine learning | Use of Virtual World/Neurofeedback with Real-Time fMRI as therapy for PTSD,Study of wearable technology to assess treatment success in PTSD | No | 2 | 1 | 1 | 1 | CAPS-5 PTSD Criteria | No | NA |
| **NCT04507360** | Enhancing Engagement With Digital Mental Health Care | 2025/8/3 | Michael Pullmann, PhD | University of Washington | United States | Completed | NA | 78390 | Psychiatry | Interventional | MHA and TS | Machine learning, Natural Language Processing | create a digital mental health research platform leveraging MHA and TS's marketing platforms and consumer base to describe the characteristics of optimal engagement with digital mental health treatment, and to identify effective, personalized methods to enhance motivation to engage in digital mental health treatment in order to improve mental health outcomes | No | 3 | 2 | 1 | 1 | Study 1: Mental Health America Disengagement (After Stage 1 Randomization),Mental Health America Engagement, Clicks on Featured Resources (After Stage 2 Randomization),Mental Health America Engagement, Number of Webpages Clicked (After Stage 2 Randomization),Mental Health America Disengagement (After Stage 2 Randomization),DIY Completion Rate (After Stage 2 Randomization);Study 2: Engagement (Dosage),Tool Use Helpfulness,Emotion Mechanisms,Tool Mechanisms,Tool Mechanisms (Part 2),DIY Skill Use: Competencies of Cognitive Therapy Scale - Self-Report;Long-term Adoption of the Intervention,Hopefulness | No | NA |
| **NCT06637774** | The Aspirometer: A Noninvasive Tool for Detecting Aspiration | 2024/10/15 | James Coyle | University of Pittsburgh | United States | Completed | NA | 50 | Respiratory | Interventional | Not mentioned | Machine-learning | discriminate normal from abnormal airway protection and kinematic functions noninvasively via machine-learning analysis of Aspirometer/HRCA (high resolution cervical auscultation) signals, with similar accuracy as human judgment of VF | Yes | 3 | 2 | 1 | 1 | Numbers of Participants for Which the Aspirometer/HRCA Predicted the Passed Versus Failed Swallow Test Result,Percentage of PAS Scores That Were Accurately Predicted by the Aspirometer/HRCA, in Comparison to Modified Barium Swallow Test. | No | algorithms, not individual participant data, will be made available to other researchers through an FTP (file transfer protocol) site |
| **NCT05554042** | Kintsugi Voice Device Study | 2022/9/26 | Grace Chang, MBA | Kintsugi Mindful Wellness, Inc. | United States | Completed | NA | 132 | Psychiatry | Observational | Kintsugi Voice Device | Machine learning | evaluate the ability of the Device to aid clinical assessment for depression and anxiety by comparing its output with the established diagnostic standard consisting of a diagnosis made by a specialist clinician based on DSM-5 criteria | Yes | 3 | 2 | 1 | 1 | Sensitivity and Specificity of the Kintsugi Voice Device for a Significant Depressive Episode, Sensitivity and Specificity of the Kintsugi Voice Device for a Significant Anxiety State | Yes | IPD will not be shared with other researchers. |
| **NCT05042063** | Acoustic Cough Monitoring for the Management of Patients With Known Respiratory Disease | 2021/9/13 | Carlos Chaccour, MD, PhD | Clinica Universidad de Navarra, Universidad de Navarra | Spain | Recruiting | NA | 100 | Respiratory | Observational | Hyfe | Convolutional Neural Networks (CNN) | evaluate the potential use of Hyfe Cough Tracker (Hyfe) to screen for, diagnose, and support the clinical management of patients with respiratory diseases, while enriching a dataset of disease-specific annotated coughs, for further refinement of similar systems | No | 3 | 2 | 1 | 1 | Correlation between subjective perception of cough and objective frequency | No | Datasets with anonymized IPD, including cough registries and VAS scores will be shared at the end of the study.IPD Sharing Supporting Information Type: Study Protocol Informed Consent Form (ICF) Clinical Study Report (CSR) |
| **NCT07183111** | Comparison of Traditional Instructions Vs AI Based Instructions With Customized Chat GPT as Remote Support System on Education of Orthodontic Patients:A Randomised Control Trial | 2025/9/19 | NA | Pakistan Institute of Medical Sciences | Pakistan | Recruiting | NA | 60 | Dentistry | Interventional | Chat GPT | Not mentioned | assess the impact of voice instructions delivered by advance voice feature of Chat gpt followed by remote support with chatbot in form of customized chat gpt named Brace AI which is customized by principal investigator | yes | 3 | 2 | 1 | 1 | Plaque index, Modified gingival index | No | NA |
| **NCT05863494** | Toward Personalized Treatment of Chronic Pain Using Transcranial Direct Current Stimulation Paired With Deep Learning | 2023/5/18 | Allison J Huff, DHEd | University of Arizona | United States | Unknown status | NA | 40 | Rehabilitation | Interventional | Not mentioned | Deep learning | apply transcranial direct current stimulation (tDCS) as an alternative to opioids for the reduction in chronic pain | No | 2 | 2 | 1 | 1 | Determine the impact of tDCS on pain in chronic pain participants using pain perception scale, and the impact of tDCS on the self-reported reduction in opioid use, or the desire for opioid use. Compare the safety of the tDCS system (tKIWI) versus placebo (sham) utilizing blood pressure, the tDCS system (tKIWI) versus placebo (sham) utilizing heart rate, and the tDCS system (tKIWI) versus placebo (sham) utilizing heart rate | Yes | Study results will not be shared with participants. They will be shared via publication in peer-reviewed journals. The data-sharing agreement with our industry partner will use deidentified data from tKIWi to strengthen the algorithm and patented machine learning. The data will be stored on an encrypted SD card and shared with our industry partner via upload to HIPAA Compliant Google Workspace. |
| **NCT04614376** | A Machine Learning Approach to Alzheimer's Detection From Continuous Blood Glucose Monitoring Data | 2020/11/4 | NA | Bio Conscious Technologies Inc. | Canada | Unknown status | NA | 100 | Neurology | Observational | Endobits Companion | Machine learning | keep track of patients diabetes management through a journal and relay this information with their CGM readings to their caring physician | Yes | 2 | 1 | 1 | 1 | Blood Glucose Levels | Yes | NA |
| **NCT06065319** | Estimating Recovery in Cardiac Rehabilitation Using Mobile Health Technology and Personalized Machine Learning | 2023/10/3 | Thomas Kurian | Texas A&M University | United States | Active, not recruiting | NA | 18 | Cardiology | Observational | Not mentioned | Machine learning | collect wearable sensor data that the investigators hypothesize will be useful in future algorithm development for monitoring recovering of participants enrolled in cardiac rehabilitation programs | No | 2 | 1 | 1 | 1 | Improvement in cardiac function | No | Deidentified wearable data and 6minute walk data will be made available, upon reasonable request, for other researchers.IPD Sharing Supporting Information Type:Study Protocol Informed Consent Form (ICF) Analytic Code |
| **NCT05735288** | Pilot-scale, Single-arm, Observational Study to Assess the Utility of a Machine Learning Algorithm in Assessing Fluid Status in Haemodialysis Patients | 2023/2/21 | O'Seaghdha | Royal College of Surgeons, Ireland | Ireland | Completed | NA | 24 | Cardiology | Observational | Not mentioned | Machine learning | assess the validity and reproducibility of an algorithm for assessing fluid status in a cohort of dialysis patients | Yes | 2 | 2 | 1 | 1 | The primary objective is to determine the validity of the machine learning model in estimating bioimpedance-determined dry weight in haemodialysis patients. | Yes | NA |
| **NCT06303986** | A Multi-center Study to Collect Data for Basic Physiological Research in Neonatal Abstinence Syndrome (NAS) and Evaluate the Automated Finnegan/ESC Data Collection Process | 2024/3/12 | Nitin Chouthai, MD | Rekovar Inc. | United States | Enrolling by invitation | NA | 100 | Neonatology | Observational | Not mentioned | Machine learning | collect data that will be used to develop an AI-based tool that can automate scoring with predictive analytics, establish the advantages of continuous monitoring in NAS that should lead to decreased length of stay in the NICU and improved patient outcomes. | Yes | 2 | 2 | 1 | 1 | Training/validation data collection | No | NA |
| **NCT06579768** | Preoperative Differentiation of Jaw Cystic Lesions Based on Radiomics From Computed Tomography Images: A Multicenter, Prospective Machine Learning Study | 2024/8/30 | NA | Sun Yat-Sen Memorial Hospital of Sun Yat-Sen University | China | Recruiting | NA | 300 | Stomatology | Observational | Not mentioned | Machine learning | enhance a previously developed predictive model that integrates machine learning with CT radiomics | Yes | 3 | 2 | 1 | 1 | Statistical Analysis Metrics for Machine Learning Model Predictions | No | NA |
| **NCT04577573** | Cognitive-based Rehabilitation Platform of Hand Grasp After Spinal Cord Injury Using Virtual Reality and Instrumented Wearables | 2020/10/8 | Noam Y. Harel, MD PhD,James J. Peters | VA Office of Research and Development | United States | Completed | NA | 13 | Neurology | Interventional | Not mentioned | Machine learning | investigate how the "cognition" glove may improve functional grasp, investigate how Veterans with SCI may learn greater arm muscle control during virtual reaching while using a "sensory" brace that provides isometric resistance to one arm to elicit electromyography (EMG) patterns that can drive a virtual arm | No | 2 | 1 | 1 | 1 | Percent Change in Time to Achieve Secure Grasp (Cognition Glove Only), Percent Change in Time to Complete Pick-up and Placement of Object-Cognition Glove, Percent Change in Time to Complete Trial-Sensory Brace | No | A Limited Dataset (LDS) will be shared in electronic format pursuant to a VA-approved Data Use Agreement. This will include all outcomes data and deidentified demographics.Individually Identifiable Data will be shared pursuant to valid HIPAA Authorization, Informed Consent, and an appropriate written agreement limiting use of the data to the conditions as described in the authorization and consent, and a written assurance from the recipient that the information will be maintained in accordance with the security requirements of 38 CFR Part 1.466. |
| **NCT03458806** | Phono- and Electrocardiogram Assisted Detection of Valvular Disease | 2018/3/8 | John Chorba, MD | University of California, San Francisco | United States | Completed | NA | 156 | Cardiology | Observational | Eko Duo | Machine learning | develop an automated system to identify VHD by phono- and electrocardiogram | No | 3 | 2 | 1 | 1 | Differentiation of clinically significant aortic stenosis from structurally normal hearts, Differentiation of clinically significant mitral stenosis from structurally normal hearts | No | We will create several de-identified databases of information and will be open to requests to share data as requested on a case-by-case basis. |
| **NCT06915909** | Robotic Prostatectomy Artificial Intelligence Low Pressure Pain Study Trial - "The Monitoring of Patients Outcomes Intraoperatively and Perioperatively Using the Airseal and Stryker Insufflator Undergoing Robotic Assisted Laparoscopic Prostatectomy at a Pressure and Stability of Pneumoperitoneum of 8 mmHg" | 2025/4/8 | NA | East and North Hertfordshire NHS Trust | United Kingdom | Not yet recruiting | NA | 40 | Oncology, Urology | Interventional | the Conventional Insufflator System (CIS), the AIRSEAL Insufflation System (AIS) | Not mentioned | compare intra and post-operative pain when using two different insufflator devices when performing robotic prostatectomies | Yes | 2 | 1 | 1 | 1 | Recruitment rates, Evaluate treatment-based adverse events, serious adverse events, anticipated adverse device effects, unanticipated adverse device effects and all device deficiencies and use errors, regardless of relationship to an adverse event. Pre-op vitals, Pre-op BMI (height and weight), Pre-op labs, Insufflation Pressure, Estimated blood loss, Blood transfusion, Procedure time (initial incision to closure), Surgeon-determined need to increase IAP beyond "study pressure", Administration of transversus abdominis plane block, Anesthetic/pain medication administration, MedaSense PMD-200 monitor for pain documentation, Intraoperative Peak Airway Pressure every 15 minutes, Intraoperative End Tidal Carbon Dioxide (ETCO2) every 15 minutes, Time in recovery room, Post-op pain incidence and severity, Post-op analgesia usage, Post- op labs as per standard of care, Vital signs 24 hr, Presence or absence of postoperative nausea or vomiting, Length of hospital stay, Any complications that were observed. Post-operative complications through 30 days, reported using Clavien-Dindo Classification, including 30-day mortality, Return to operating room within 24 hour, Readmission to hospital within 30 days, All Adverse Events (intra-operative and post-operative through 30 days) | Yes | NA |
| **NCT05537792** | User-Independent Intent Recognition on a Powered Transfemoral Prosthesis | 2022/9/13 | Aaron Young, Ph.D. | Georgia Institute of Technology | United States | Completed | NA | 10 | Rehabilitation | Interventional | Not mentioned | Machine learning | a proposed novel AI system to self-adapt an intent recognition system in powered prostheses to aid deployment of intent recognition systems that personalize to individual patient gait | No | 3 | 2 | 1 | 1 | Overground Self-selected Walking Speed | No | NA |
| **NCT04502563** | Continuous Wearable Monitoring Analytics to Improve Outcomes in Heart Failure - LINK-HF2 Multicenter Implementation Study | 2020/8/6 | Josef Stehlik, MD MPH | VA Office of Research and Development | United States | Completed | NA | 208 | Cardiology | Interventional | LINK-HF2 | Machine learning | Implement remote monitoring into the clinical workflow of HF care, Conduct a feasibility study of non-invasive remote monitoring in chronic HF | Yes | 3 | 2 | 1 | 1 | Heart failure hospitalization rate | Yes | NA |
| **NCT06183138** | Multicenter Analysis of Genomic and Metabolic Data of Neonatal Genetic Diseases | 2023/12/27 | NA | Sixth Affiliated Hospital, Sun Yat-sen University | China | Recruiting | NA | 40000 | Clinical Genetics | Observational | Not mentioned | genome and metabolome big data and Machine learning | construct prediction models for common genetic diseases, and strive to achieve accurate diagnosis and prediction of common genetic diseases using simple tandem mass spectrometry metabolome data, and expand the application range of tandem mass spectrometry technology for disease detection | No | 2 | 1 | 1 | 1 | Number of gene sequencing data in neonatal gene bank, Gene mutation rate | No | Privacy protection measures: All the data of the subjects during the study period will be entered into the computer for confidential storage and analysis. If necessary, the relevant institutions may review the records to confirm the authenticity, accuracy and integrity of the data. The data obtained from the study may also be published in academic journals, but the names of the subjects will not be published, and the privacy of the subjects will be kept confidential. |
| **NCT05918003** | Validation by Three-dimensional Actimetry Measurements of a Predictive Algorithm for Excessive Inactivity in Chronic Obstructive Pulmonary Disease (COPD) Patients | 2023/6/26 | NA | Association pour la Complementarite des Connaissances et des Pratiques de la Pneumologie | France | Unknown status | NA | 104 | Respiratory | Observational | Not mentioned | Machine learning | verify and improve the validity of the MLA on a new smaller population of 104 patients, using a physiological GOLD standard such as three-dimensional actimetry | No | 3 | 2 | 1 | 1 | AUC-ROC as the primary endpoint to judge the performance of the algorithm | Yes | Undecided to Share Individual Participant Data (IPD)to Share Individual Participant Data (IPD) |
| **NCT04154904** | Development of a Context-aware Glucose Prediction Algorithm in Patients With Type 1 Diabetes | 2019/11/7 | Jessica Castle, MD | Oregon Health and Science University | United States | Completed | NA | 30 | Endocrinology | Interventional | Not mentioned | Not mentioned | the creation of a data set that will include contextual patterns along with glucose, insulin and physiological data | No | 3 | 1 | 1 | 1 | Comparison of the Mean Absolute Relative Difference When Including Patterns in Hypoglycemia Prediction. Comparison of the Mean Relative Difference When Including Patterns in Hypoglycemia Prediction. | Yes | NA |
| **NCT04184791** | Computational Modeling of 60 Hz Subthalamic Nucleus Deep Brain Stimulation for Gait Disorder in Parkinson's Disease | 2019/12/4 | Ritesh Ramdhani, MD | Northwell Health | United States | Completed | NA | 24 | Neurology | Interventional | Not mentioned | Machine learning | further the understanding and application of 60Hz subthalamic deep brain stimulation (STN-DBS) in Parkinson's patients with gait disorder | No | 2 | 1 | 1 | 1 | Gait Kinematic Response for 180 and 60Hz DBS, Accuracy of Discriminating STN-DBS (60hz vs. High Frequency) and Medication States With Machine Learning(ML) | No | NA |
| **NCT05231954** | Digital Detection of Dementia (D Cubed) Studies: D2 | 2022/2/9 | Malaz Boustani, MD | Indiana University | United States | Completed | NA | 5325 | Neurology | Interventional | Not mentioned | Machine learning | evaluate the practical utility and effect of the PDM, the QDRS, and the combined approach (PDM + QDRS) in improving the annual rate of new documented ADRD diagnosis in primary care practices | No | 3 | 1 | 1 | 1 | 12-Month Cumulative Incidence of ADRD Diagnoses | Yes | NA |
| **NCT05802771** | Lung Cancer Multi-omics Digital Human Avatars for Integrating Precision Medicine Into Clinical Practice: the LANTERN Study | 2023/4/7 | NA | Fondazione Policlinico Universitario Agostino Gemelli IRCCS | Italy | Not yet recruitin | NA | 600 | Respiratory, Oncology | Observational | Not mentioned | Machine learning, Applying Text Mining, Natural Language Processing | To develop prevention models for early lung cancer diagnosis;To set up personalized predictive models for individual-specific treatments | No | 1 | 1 | 2 | 1 | To develop prevention models for early lung cancer diagnosis | Yes | NA |
| **NCT05600101** | Development of Avatar-based Intervention to Support Patients Undergoing Reduced-Intensity Allogenic Stem Cell Transplantation | 2022/10/31 | Gregory Abel, MPH, MD | Dana-Farber Cancer Institute | United States | Completed | NA | 18 | Oncology | Interventional | Care.coach | Not mentioned | communicate with participants regarding transplantation education and provide reminders for eating, drinking, and activity | No | 1 | 1 | 2 | 1 | Number of Participants Retained | No | The Dana-Farber / Harvard Cancer Center encourages and supports the responsible and ethical sharing of data from clinical trials. De-identified participant data from the final research dataset used in the published manuscript may only be shared under the terms of a Data Use Agreement. Requests may be directed to: [contact information for Sponsor Investigator or designee]. The protocol and statistical analysis plan will be made available on Clinicaltrials.gov only as required by federal regulation or as a condition of awards and agreements supporting the research |
| **NCT06443073** | The Mere-measurement Effect in Patient-reported Outcomes: A Randomized Control Trial With Speech Pathology Patients | 2024/6/5 | NA | Medical University of Vienna | Austria | Recruiting | NA | 170 | General medicine | Interventional | ChatGPT | Not mentioned | recreate novel text of the same length and difficulty as the "Rainbow Passage" which is called "In the heart of a lush valley"；speech analysis | No | 1 | 1 | 1 | 1 | audio recording of a standardized text | No | Patients will consent that pseudonymized patient data will be hosted and analysed at the MUW. MUW will not share the data with any third parties or collaborators. MUW regularly processes medical data and is subject to the GDPR requirements. MUW is responsible for data quality and will perform oversight of the data management of this study. Investigators and study coordinators will receive an initial training session on the protocol, study flow, study database, survey tool, documentation, and expectations, and any applicable study processes. Access to data for secondary use cases will not be enabled. |
| **NCT06365099** | dentifying Personalized Brain States Predicting Residual Corticospinal Tract Output After Stroke | 2024/4/15 | NA | University of Texas at Austin | United States | Recruiting | I | 20 | Neurology | Interventional | Not mentioned | Machine learning | identifies personalized brain activity patterns reflecting strong CST activation | No | 1 | 2 | 1 | 2 | Personalized classifier performance | Yes | NA |
| **NCT05506358** | Evaluation of Low-cost Techniques for Detecting Sickle Cell Disease and β-thalassemia in Nepal and Canada | 2022/8/18 | Boris Stoeber | University of British Columbia | Canada，Nepal | Completed | NA | 145 | Hematology | Interventional | Not mentioned | Machine learning | classify SCD, SCT and healthy individuals | No | 1 | 1 | 1 | 1 | Sensitivity, Specificity, Positive Predictive Value and Negative Predictive Value | No | Only de-identified data and test results will be shared. The test results for the low-cost tests and HPLC tests will be published in aggregate form. De-identified images of blood films will be deposited in an online public repository, such as Federated Research Data Repository (FRDR). |
| **NCT03930199** | Personalized Mobility Interventions Using Smart Sensor Resources for Lower-Limb Prosthesis Users | 2019/4/29 | Arun Jayaraman, PT, PhD | Shirley Ryan AbilityLab | United States | Active, not recruiting | NA | 66 | Rehabilitation | Interventional | Not mentioned | Machine-learning and Data-mining techniques | identify a subset of measures from this toolbox that sensitively and accurately reflect real-world function, enabling clinicians to predict and assess activity and provide effective interventions to optimize prosthesis use | No | 1 | 1 | 1 | 1 | Pre-intervention:10 Meter Walk Test (10MWT)，Pre-intervention: 6 Minute Walk Test (6MWT)，Pre-intervention: Berg Balance Scale Test，Post-intervention:10 Meter Walk Test (10MWT)，Post-intervention: 6 Minute Walk Test (6MWT)，Post-intervention: Berg Balance Scale Test，Delayed post-intervention:10 Meter Walk Test (10MWT)，Delayed post-intervention: 6 Minute Walk Test (6MWT)，Delayed post-intervention: Berg Balance Scale Test | Yes | NA |
| **NCT07159711** | Evaluation of Seismocardiography (SCG) as a Tool for Assessing Fitness and Predicting Outcomes in Oesophageal Cancer Surgery | 2025/9/8 | Andrew Davies, MD | Guy's and St Thomas' NHS Foundation Trust | United Kingdom | Not yet recruiting | NA | 164 | Oncology | Observational | Seismofit® | Machine learning | calculate the VO2 peak to estimate fitness | No | 1 | 1 | 1 | 1 | Rate of pneumonia as defined by the Esophageal Complications Consensus Group criteria (ECCG)，Overall rate of complications as defined by the Clavien-Dindo classification，Total hospital length of stay for patients being treated for Oesophageal cancer | Yes | All collected pseudonymised datapoints that underlie results in publication will be shared. The individual consent forms will be stored at each of the research sites. At the time of upload to the REDCap research database, a case reporting form code number will be generated. Patient identifiable information will not leave the research site at which the patient receives treatment. |
| **NCT05789875** | Youth Ending the HIV Epidemic - Automated Directly Observed Therapy Pilot: Improving HIV Care Among Youth | 2023/3/29 | Parya Saberi, PharmD | University of California, San Francisco | United States | Completed | NA | 28 | Infectious Diseases | Interventional | AiCure | computer vision and deep learning algorithms | track and support ART adherence | No | 1 | 1 | 1 | 1 | Feasibility: Rate of Participant Retention，Feasibility: Mean Logins Per Week，Feasibility: Mean Number of Seconds in App Per Day，Feasibility: Mean Percent Doses a Participant May Have "Falsified" Med-taking，Acceptability: System Usability Scale (SUS) >68, Considered Above Average and Acceptable，Acceptability: Client Satisfaction Questionnaire (CSQ-8) Score of ≥17, Considered Above Average and Acceptable，Acceptability: Likelihood of Recommending the Study to a Friend (Extremely, Very)，Acceptability: Satisfaction With the App+Incentives (Mostly, Very) | No | NA |
| **NCT06796283** | Research on AIS Recurrence Risk Prediction Model Using XGBoost Combined With Convolutional Neural Network Algorithm | 2025/1/28 | yingping Y Yi | Second Affiliated Hospital of Nanchang University | China | Completed | NA | 2628 | Neurology | Observational | XGBoost | Convolutional Neural Network (CNN) algorithm | construct a recurrence risk prediction model for ischemic stroke within 1, 3, 6, and 12 months | No | 1 | 1 | 1 | 1 | Recurrent ischemic stroke | Yes | NA |
| **NCT07083791** | AI-ECG Accessory Pathway Localisation Study | 2025/7/24 | Ahran Arnold, PhD | Imperial College London | United Kingdom | Not yet recruiting | NA | 100 | Cardiology | Observational | Not mentioned | AI-ECG algorithm | identifying the location of an accessory pathway from the 12-lead electrocardiogram | No | 1 | 1 | 1 | 1 | Performance and accuracy of the AI-ECG accessory pathway localisation algorithm | No | NA |
| **NCT04906135** | Auditory Neural Function in Implanted Patients With Usher Syndrome | 2021/5/28 | Shuman He, MD, PhD | Ohio State University | United States | Completed | NA | 29 | Otology | Interventional | Not mentioned | Supervised Machine learning | develop an objective tool for assessing the electrode-neuron interface at individual electrode locations. | No | 2 | 2 | 1 | 2 | The electrically evoked compound action potential | No | NA |
| **NCT06762613** | Enhancing CBCT-Guided Lung Nodule Puncture Efficiency With Generative AI | 2025/1/7 | NA | Union Hospital, Tongji Medical College, Huazhong University of Science and Technology | China | Completed | NA | 220 | Respiratory | Interventional | Generative AI Based Puncture Surgery Navigation System (GPS) | Not mentioned | guide lung puncture in adults better，quality improvement of cone beam CT (CBCT) by GPS | Yes | 2 | 2 | 1 | 2 | Number of puncture needles of participants for GPS and CBCT-guided lung puncture procedures. | Yes | NA |
| **NCT02990377** | Reducing Non-Medical Opioid Use: An Automatically Adaptive mHealth Intervention | 2016/12/13 | Amy S Bohnert, Ph.D. | University of Michigan | United States | Completed | NA | 459 | Emergency department | Interventional | Not mentioned | Reinforcement learning | select the action most likely to reduce non-medical opioid use | No | 1 | 1 | 2 | 1 | Level of Non-Medical Opioid Use | Yes | NA |
| **NCT05797974** | Does a Virtual Coach Offer a Better Solution for Weight Reduction in Ventral Hernia Patients With Obesity? | 2023/4/4 | Jana Sacco, MD | University of Florida | United States | Completed | NA | 33 | Endocrinology | Interventional | Not mentioned | Machine learning | determine the important features for weight lost | No | 1 | 1 | 1 | 1 | pounds of weight change per group | No | NA |
| **NCT06194526** | Whole Blood Transcriptomic Signal According to Coronary Atherosclerotic Plaque Burden Assessed by CT Angiography: CORPLAQ-TRAIT Pilot Study | 2024/1/8 | Rosana Poggio, MD MSc PhD | MultiplAI Health Limited | Argentina | Enrolling by invitation | NA | 200 | Cardiology | Observational | Not mentioned | Not mentioned | detect the presence of aortic disease and pro-inflammatory cardiometabolic alterations | No | 1 | 1 | 1 | 1 | Coronary calcium score，Total coronary plaque burden，Degree of coronary stenosis，Coronary high-risk plaques (low-attenuation, positive remodeling; spotty calcification, or napkin-ring sign) | Yes | NA |
| **NCT06915285** | Analyzing the Performance of Automated Software in the Identification of Malarial Retinopathy in Digital Retinal Images of Cerebral Malaria Patients | 2025-04-08 | NA | VisionQuest Biomedical LLC | Malawi | Completed | NA | 834 | Ophthalmology | Interventional | ASPIRE | Not mentioned | processing mydriatic retinal images of a patient with clinically diagnosed Cerebral Malaria (CM), to detect malarial retinopathy (MR). | No | 2 | 1 | 1 | 1 | Sensitivity and Specificity of ASPIRE to Detect Malarial Retinopathy in Eyes of the Subjects Clinically Diagnosed With CM | Yes | The IPD may remain as a part of trade secret and/or proprietary information for the sponsoring organization, and hence cannot be shared at individual participant's level. |
| **NCT06570486** | Contrast Between Traditional Regression Model and AI in Predicting Prolonged Stay Stay After Head and Neck Tumors | 2024-08-26 | NA | Sun Yat-Sen Memorial Hospital of Sun Yat-Sen University | China | Recruiting | NA | 700 | Oncology | Observational | Not mentioned | Not mentioned | compared the prediction effect of AI and the traditional prediction model on whether patients can be transferred to ICU within 24 hours of head and neck tumors. | No | 1 | 1 | 1 | 1 | roll-out icu | No | If others need it, they can apply to us for the data, and we will provide it. |
| **NCT06324981** | Using Artificially Intelligent Text Messaging Technology to Improve American Heart Association's Life's Essential 8 Health Behaviors: LE8 Bot + Backup | 2024-03-22 | P. Michael Ho, MD PhD | University of Colorado, Denver | United States | Recruiting | NA | 2100 | Cardiology | Interventional | AI chatbot | Not mentioned | have the promise of improving the impact of text messaging, particularly if they integrate evidence based communication strategies, including tailoring, behavioral nudges that support intuitive decision-making, and persuasive messaging. | Yes | 1 | 1 | 2 | 1 | Change in Life's Essential 8 risk score | No | NA |
| **NCT05447884** | Learning-based Control of a Hip Exoskeleton to Improve Balance and Energetics of Human Walking Functions | 2022-07-07 | Helen Huang, Ph.D.,Michael D. Lewek, Ph.D. | North Carolina State University | United States | Recruiting | NA | 100 | Sports Medicine | Interventional | Not mentioned | Reinforcement learning | increase the assistive outcomes and guarantee safety when using exos | No | 1 | 1 | 1 | 1 | Human lower limb joints angular position,Human lower limb joints angular velocity | Yes | NA |
| **NCT06264479** | Prospective Evaluation of AI R&D Tool for Patient Stratification: a Trial for Renal Immuno-oncology Model Experimental Evaluation 2 | 2024-02-20 | Ekaterini Boleti | Ourotech, Inc. | United Kingdom | Terminated | NA | 7 | Oncology | Observational | Not mentioned | Not mentioned | measure the response of an individual patient's tumor sample to different systemic therapy regimens that are tested simultaneously ex vivo. | No | 2 | 1 | 1 | 1 | Objective Response Rate correlation accuracy (sensitivity & specificity) | Yes | Pseudonymised data will be shared between study sites, such as treatment and outcome data for each patient. No publication will be made revealing individual patient data; only group-level data will be published. |
| **NCT07169461** | Effectiveness of Low Level Light Therapy and Intense Pulse Light on Mite Count as Adjuntive Therapies in Demodex Blepharitis Using Artificial Intelligent Program (Ai-Demodex): A Factorial Randomized Sham-Controlled Trial | 2025-09-11 | Vannarut Satitpitakul, MD | Chulalongkorn University | Thailand | Enrolling by invitation | NA | 88 | Ophthalmology | Interventional | Ai-Demodex | Not mentioned | To evaluate the efficacy of LLLT, IPL, and IPL + LLLT in re ducing demodex count in patients with Demodex Blepharitis. | No | 1 | 1 | 1 | 1 | Mean of Demodex Count in 30 days after treatment | No | NA |
| **NCT06256978** | Continuous Temperature Measurement by Thermal Imaging Camera: Concordance, Patterns, and Intelligent Prediction of Events in Critical Patients | 2024-02-13 | NA | Universidad Europea de Madrid | No location data | Not yet recruiting | NA | 224 | Critical Care Medicine | Observational | Not mentioned | Machine learning | develop predictive models for critical events such as the onset of fever, nosocomial infections, or shock. | No | 1 | 1 | 1 | 1 | Concordance between thermal imaging camera and axillary contact thermometer | Yes | NA |
| **NCT03683472** | Developing a Novel Digital Therapeutic for the Treatment of Generalized Anxiety Disorder | 2018-09-25 | Judson Brewer | MindSciences, Inc. | United States | Completed | NA | 65 | psychiatry | Interventional | Not mentioned | Not mentioned | (1) develop and refine Unwinding Anxiety for individuals with generalized anxiety disorder, (2) determine user engagement and acceptability as well as measure effect sizes of the program vs. treatment as usual, and (3) preliminarily test mechanisms of action. | No | 1 | 1 | 1 | 1 | UA Program engagement,Change in worry | No | NA |
| **NCT05612217** | A Study to Evaluate a Accuracy of the AIVARIX AI-based Application in Detecting Signs C 1-2 Classes of CVD in Outpatients Seeking Consultancy of Phlebologists in the Russian Federation | 2022-11-10 | IGOR ZOLOTUKHIN | Servier Russia | Russia | Completed | NA | 433 | Cardiology | Observational | AIVARIX AI-based Application | Not mentioned | detecting C1 and C2 classes by CEAP classification of chronic venous disease (CVD) in patients who are consulted by phlebologists on symptoms and signs suggestive to CVD. | No | 1 | 1 | 1 | 1 | Evaluation of Sensitivity (Sn) of the AI-based AIVARIX App in Detecting C1 and C2 Classes of CVD in Patients Who Are Seeking for Professional Advice From a Phlebologist Regarding Symptoms and Signs Suggestive to CVD.Evaluation of Specificity (Sp) of the AI-based AIVARIX App in Detecting C1 and C2 Classes of CVD in Patients Who Are Seeking for Professional Advice From a Phlebologist Regarding Symptoms and Signs Suggestive to CVD | Yes | NA |
| **NCT06988189** | Unmasking Concealed Arrhythmia Syndromes | 2025-05-23 | Zachary Whinnett, PhD | Imperial College London | United Kingdom | Recruiting | NA | 200 | Cardiology | Observational | Not mentioned | Not mentioned | automate the recognition of the type 1 Brugada ECG pattern on 12 lead ECGs. | No | 1 | 1 | 1 | 1 | Sensitivity, specificity, and area under the curve (AUC) of AI algorithm for detection of Brugada type 1 ECG pattern on 12-lead ECGs.Detection rate of Brugada ECG pattern using extended-duration multi-electrode ambulatory ECG monitoring (wearable ECG) in patients with concealed Brugada syndrome.Number of cases of Brugada or Long QT Syndrome (LQTS) detected using extended-duration multi-electrode ambulatory ECG monitoring in patients with idiopathic ventricular fibrillation (VF), after application of AI ECG detection algorithms. | Yes | NA |
| **NCT05390684** | Predictive Model to Early Diagnosis of Anastomotic Leak After Esophagectomy and Gastrectomy. PROFUGO Study. | 2022-05-25 | Marcos Bruna | Hospital Universitario La Fe | Spain | Unknown status | NA | 800 | Gastroenterology | Observational | Not mentioned | Not mentioned | allows identifying cases with a high risk of anastomotic leak and/or major complications through the analysis of different clinical and analytical variables collected during the postoperative period. of patients undergoing esophagectomy or gastrectomy. | No | 2 | 1 | 1 | 1 | anastomotic leak | Yes | NA |
| **NCT06848036** | Effectiveness of a Smart Community- and Home-based Integrated Care Services System for Elderly People in Rural Chinese Communities: A Randomized Controlled Trial in Changsha County | 2025-02-26 | Zhihan Liu | Liu Zhihan | China | Active, not recruiting | NA | 64 | Geriatrics | Interventional | Not mentioned | Not mentioned | ensure that key elderly groups with disabilities, dementia, chronic diseases, advanced age, and disabilities can receive the necessary medical services at home. This not only allows the elderly to live in familiar home environments, maintaining their independence and dignity, but also alleviates the pressure on medical resources, enabling more resources to be allocated to emergency care and highly specialized nursing. | No | 1 | 1 | 1 | 1 | SF-36 scale (Short Form 36 Health Survey) | Yes | NA |
| **NCT06421402** | Knowledge for Improving Indoor Air Quality and Health: Follow up of 200 High-risk Chronic Respiratory Patients During 24 Months. | 2024-05-20 | JOSEP ROCA TORRENT, MD, PHD | Institut d'Investigacions Biomèdiques August Pi i Sunyer | Spain | Enrolling by invitation | NA | 200 | Respiratory | Observational | Not mentioned | Machine learning | early detection and management exacerbations. | No | 2 | 1 | 1 | 1 | Changes in use of healthcare resources - Unplanned hospital admissions,Changes in use of healthcare resources - Exacerbations,Changes in use of healthcare resources - Emergency room visits,Changes in use of healthcare resources - Primary care visits. | No | During the entirety of the project, it is not allowed to disseminate data. Nonetheless, should a formal request be submitted, it may be permissible to disclose certain elements of the information, albeit not in its totality. |
| **NCT06337526** | Identifying Factors Associated With Acute Pain Exacerbation in Children With Complex Regional Pain Syndrome (CRPS): A Novel Research Plan | 2024-03-29 | ANDREW DINH, MD | Stanford University | United States | Not yet recruiting | NA | 150 | Neurology | Observational | Medeloop | Not mentioned | photograph all meals for analysis of the dietary content by AI, which will be transmitted to Medeloop after capture for AI analysis | No | 2 | 1 | 1 | 1 | Change from baseline in pain score | No | NA |
| **NCT05454514** | Automated Medication Platform With Video Observation and Facial Recognition to Improve Adherence to Antiretroviral Therapy in Patients With HIV/AIDS | 2022-07-12 | NA | Charles Gellman | United States | Completed | NA | 7 | Nursing | Interventional | HiDO | Not mentioned | integrates medication dispensing, pill count and a front-facing video cameras to confirm the right medications are given at right time to the right patient | No | 1 | 1 | 1 | 1 | Rate of Medication Adherence | No | Results will not be published. |
| **NCT04447794** | Can a Chatbot-delivered Alcohol Intervention Engage Users and Enhance Outcomes Over a Smartphone App? Development and Feasibility Testing of a StepAway 'Bot' | 2020-06-25 | Patrick Dulin, PhD | University of Alaska Anchorage | United States | Completed | NA | 191 | Psychiatry | Interventional | StepAway 'Bot' | Not mentioned | holds potential for providing enhanced user engagement and effectiveness as it can reach out through a text interface to introduce new intervention steps and respond to the user with Step Away's in-the-moment help with having a craving, experiencing distress or needing social support. | No | 1 | 1 | 2 | 1 | Change in Alcohol Consumption (Drinks Per Day),Change in Alcohol-Related Problems,Change in Alcohol Consumption (Heavy Drinking Days),Change in Alcohol Consumption (Percentage of Days Abstinent) | No | NA |

**Supplementary material 4.** Detailed data of informed consent

| **NCT Number** | **Disclosure of basic information (Trial name, related institution, ethical approval)** | **Disclosure of type of AI been used (e.g. mentioning of machine learning or large language model)** | **Disclosure of AI's role in the trial (e.g. diagnostic support, risk prediction)** | **Disclosure of protection for the vulnerables** | **Disclosure of benefits in using AI** | **Disclosure of risks** | **SMOG Readability** | **Characters** | **Sentences** | **Includes visual aid for understanding** | **Disclosure of data usage** | **Disclosre of data storage** | **Mentioning of government policy** | **Disclosure of rights to quit** | **Future usage** | **Contact infotmation** | **Data usage after withdrawal** |
| --- | --- | --- | --- | --- | --- | --- | --- | --- | --- | --- | --- | --- | --- | --- | --- | --- | --- |
| NCT02176226 | Yes (Trial name, related institution, ethical approval) | Machine learning | Therapeutic support (Aims to create a mobile phone intervention that utilizes “machine learning” to provide care for depression and anxiety) | To protect vulnerable populations, researchers may also need to break confidentiality if participants report knowledge of abuse or neglect of a child, elderly person, or disabled individual. | Improved patticipant mood or quality of life, helping to shape a new intervention for people experiencing depression or anxiety, help scientists to better understand how to treat depression and anxiety using new technologies | May experience emotional discomfort or increased anxiety | 14.3 | 17418 | 126 | NA | Collecting data about how and when participants use the IntelliCare apps, and other applications on paticipant's smartphone. Researchers will also collect information data on they location, movement, WiFi network, surrounding Bluetooth devices, mobile phone usage, and sounds in pariticipants environment | Data is stored on our secure servers, it will not contain any identifying information about participants | NA | Yes | Data will be use in the future study with additional consent | Principal Investigator | NA |
| NCT02801877 | Yes (Trial name, related institution, ethical approval) | NA | Therapeutic support (The AI, via the IntelliCare suite of apps, teaches skills to manage mood, helps anticipate stressful times, and provides in-the-moment tools for managing anxiety and sadness.) | To protect vulnerable populations, confidentiality may be broken if a participant reports being a danger to themselves or others, or reports knowledge of abuse or neglect of a minor, elderly person, or disabled individual. | Possible improvements in mood or quality of life, helping researchers better understand how to treat depression and anxiety, and continued independent use of apps after the study. | May experience emotional discomfort or increased anxiety from answering questions about mental health or from reactions to study materials. | 13.74 | 16263 | 118 | Yes | Collecting data on app usage, interactions with study staff (via phone, email, text), and information provided through study assessments. Audio recordings of phone calls may also be collected. | Data containing direct identifiers is kept in secure, password-protected, and locked files. App usage data is stored on secure servers in a format with no identifying information. | NA | Yes | Data will be use in the future study without additional consent | Principal Investigator | NA |
| NCT03685240 | Yes (Trial name, related institution, ethical approval) | AI-enabled cameras ( Human in Loop) | Risk prediction (Fall detection and event detection) | Participants are residents with dementia who cannot consent; consent is provided by Legally Authorized Representatives. Residents can non-verbally object, leading to camera covering/removal. Cameras are not placed in bathrooms. No audio recording. Signs are posted on doors. | Faster response times to falls, reduced time spent on the ground, reduced unnecessary ER visits, reduced overall fall rates through root cause analysis, improved safety for residents who cannot advocate for themselves. | Loss of privacy. False alarms can cause video to be uploaded at any time. The system is not designed to detect abuse or theft, so such incidents may be missed. | 16.49 | 27749 | 188 | Yes | Video is recorded and uploaded only when a fall-related event is detected. Data collected includes time of fall, time to staff notification, time on ground, demographics, medical conditions, ER visits, and hospitalizations. | Video is stored encrypted on a password-protected computer at the facility and transmitted via encrypted channel to SafelyYou. Data is stored on a password-protected, HIPAA-compliant AWS website. Data is retained for 5 years post-study. | Mentions compliance with California Department of Social Services (DSS) Community Care Licensing Division guidelines for video recording and Welfare and Institutions (WIC) code for abuse reporting. | Yes | NA | Member of the research team | Data already collected will continue to be used in the research. |
| NCT03833804 | Yes (Trial name, related institution, ethical approval) | Natural Language Processing (NLP) | Diagnostic support (Aims to identify substance exposure/misuse from Electronic Medical Records) | NA | NA | NA | 29.31 | 624 | 4 | NA | Data is existing identifiable private health information from the Electronic Medical Record system, used for secondary research. | NA | Explicitly references and is granted under U.S. federal regulations: 45 CFR 46.104(d)(4), 45 CFR parts 160 and 164 (HIPAA) | NA | NA | NA | NA |
| NCT04442607 | Yes (Trial name, related institution, ethical approval) | Computer-Aided Detection (CADe) tool | Diagnostic support (Real-time polyp detection during colonoscopy withdrawal phase) | Excludes pregnant women. Participation is voluntary with right to withdraw. Data is anonymized/encoded | Potential for better polyp detection rate compared to standard colonoscopy alone. | No additional risks | 16.92 | 9936 | 53 | NA | Data collected includes video recordings of the procedure, polyp detection counts/categorizations (true positive, false negative, false positive), and histological results. Used for validating the AI tool and future research within the study's context. | Data is encoded (anonymized with an ID code) before being sent to the study data manager (Dept. of Gastroenterology, UZ Leuven). Stored on secure servers. Encoded data may be transferred internationally. Retained for research validity even if participant withdraws. | Explicitly mentions compliance with the European General Data Protection Regulation (GDPR) and the Belgian Law of 7 May 2004 related to experiments on humans. | Yes | NA | Principal Investigator | Data already collected will continue to be used in the research. |
| NCT04444908 | Yes (Trial name, related institution) | Deep learning | Diagnostic support (Real-time assessment of bowel cleanliness using the Boston Bowel Preparation Scale to assist endoscopists in deciding if a second colonoscopy is needed) | NA | Patients will likely have a better view of lesions, more accurate recommendations for follow-up colonoscopy, and more comprehensive disease information. | No additional risks | 15.82 | 8869 | 67 | NA | Endoscopic electronic images and case information will be collected and preserved in the hospital for scientific research by the doctor/researcher. | Data will be stored in the hospital,The ICF states personal identity will not be disclosed in public reports and privacy will be protected "within the law," but specific technical details (e.g., encryption, server location) are not provided. | NA | Yes | Data will be use in the future study with additional consent | NA | NA |
| NCT04458220 | Yes (Trial name, related institution, ethical approval) | AI portrait feature recognition | Risk prediction (Predict difficult airway) | NA | Participant will know if they are at risk of having a difficult airway | No additional risks | 15.82 | 4366 | 33 | NA | States that photo information will be entered into a confidential database | States data will be stored in the Big Data Research Institute; only researchers and ethic committee can access it | NA | Yes | NA | NA | Data will not be used and destroyed after withdrawal |
| NCT04749277 | Yes (Trial name, related institution, ethical approval) | CAD EYE® | Diagnostic support (Aims to optimize "detection and optical characterization of colorectal polyps" in real-time during colonoscopy.) | NA | Reduction in cancer incidence/mortality, reduced polypectomy morbidity; and economic benefits: reduced histopathology costs. | No additional risks | 18.97 | 10277 | 44 | NA | States data from clinical history, colonoscopy, AI tool, and histopathology will be collected and submitted to statistical analysis with anonymity. Also details in section 7 how data will be used, communicated to sponsor/authorities, and anonymized for publication | Partially (States data will be kept "confidential and anonymized" and that auditors/authorities may access records. It mentions computerization by the sponsor but does not specify physical or digital storage locations or retention periods like "AWS" or "5 years".) | NA | Yes | NA | Member of the research team | Data already collected will continue to be used in the research. |
| NCT04761211 | Yes (Trial name, related institution, ethical approval) | NA | Diagnostic support (The AI app analyzes photos taken by the smart bra for breast disease screening, to be compared with ultrasound/mammogram results) | NA | No direct benefit to participant. Societal benefit: Data can provide a safer and more effective choice for future women to diagnose breast diseases. | Very low risk of skin allergy from bra material. Risk of device not working if bra pressure is insufficient. | 14.75 | 14760 | 105 | Yes | Photos of 5 breast sites and patient information (name, phone, email, medical history, questionnaire data) are uploaded via the app and exported for AI learning by Tsinghua University. | Patient medical records are kept in the hospital. Implies data is stored on the app's backend/server for export, but specific storage location/security (e.g., HIPAA-compliant) is not detailed. | NA | Yes | NA | Member of the research team | NA |
| NCT04892329 | Yes (Trial name, related institution, ethical approval) | Deep learning (EndoAngel) | Diagnostic support / Procedural assistance (The AI system "can assist in identifying important anatomical structures adjacent to the pancreas in real time" to "improve the quality of EUS and reduce the missed diagnosis of pancreatic lesions") | NA | Patients in the experimental group "will likely have a better view of the lesion, a higher exam quality, and more comprehensive disease information." | No additional risks | 16.1 | 9021 | 63 | NA | Patient's EUS electronic images and case information will be collected and preserved in the hospital" for the researcher's access for "scientific research" | Data will be "preserved in the hospital" and that "researchers will do everything within the law to protect the privacy of paticipants personal medical data. | NA | Yes | Data will be use in the future study with additional consent | Member of the research team | NA |
| NCT04903444 | Yes (Trial name, related institution) | Deep learning (EndoAngel) | Diagnostic support / Procedural assistance (The AI's role is clearly defined as providing real-time instruction for the direction of the guide wire and the position of stent placement during the ERCP procedure) | NA | Patients in the experimental group (using EndoAngel) will "likely have fewer surgical times, higher surgical success rates, and lower incidences of adverse events." The protocol also mentions "improve the quality of ERCP." | No additional risks | 15.24 | 9087 | 67 | NA | During MRCP and ERCP, the patient's "electronic images and case information will be collected and preserved in the hospital" and that the doctor/researcher will be given access to this information for "scientific research." | Data will be "preserved in the hospital" and that "personal identity will not be disclosed in any public report." | NA | Yes | Data will be use in the future study with additional consent | Member of the research team | NA |
| NCT04912037 | Yes (Trial name, related institution) | Deep learning (EndoAngel) | Diagnostic support / Procedural assistance (The IC states the AI can be used for "colonoscopy quality monitoring and lesion indication in real time" | Indirectly addressed (Exclusion criteria protect vulnerable groups like those with mental disorders or substance abuse. The IC emphasizes voluntary participation and the right to withdraw, which protects autonomy) | Higher quality and more comprehensive information about their disease, lower incidences of adverse events | No additional risks | 19.53 | 7608 | 34 | NA | Endoscopic electronic image and case information will be collected for scientific research and development of new medical technologies. Public reports will not disclose personal identity. | Data will be stored in the hospital. Information will be identified by a code. Only research physician and authorized personnel can link the code to the name via a secure checklist. | NA | Yes | Data will be use in the future study with additional consent | Member of the research team | NA |
| NCT05287477 | Yes (Trial name, related institution, ethical approval) | AI Clinician XP1 | Therapeutic support (For sepsis treatment). | NA | NA | NA | 12.65 | 1082 | 9 | NA | Sections of any of my research data may be looked at by responsible individuals" and consent for future research use can be given. | NA | NA | Yes | Data will be use in the future study with additional consent | NA | NA |
| NCT05311046 | Yes (Trial name, related institution, ethical approval) | NA | Risk prediction / Diagnostic support.(Develop a screening tool that can accurately recognize sepsis earlier) | Protections include parental permission, data de-identification, secure storage, a Certificate of Confidentiality, and HIPAA compliance. The document also states the PI may withdraw a participant if it's in their best interest. | Participating in the study will not benefit to participant's child directly, but help improve the treatment of septic patients in the future | No additional risks | 14.49 | 20444 | 143 | NA | The document states data (medical record info, lab results, vital signs, etc.) will be used to "examine their response to treatment" and to "develop a screening tool." It also mentions data may be used for future research, shared with other sites, and published (de-identified). | Data will be kept "in the PI’s office in a secure location" and in "a locked cabinet on a secure and restricted research floor." Electronic data is stored on a "password protected file on the hospital server." Samples are de-identified and shipped to Children’s National Research Institute. | The document explicitly mentions compliance with the "Health Insurance Portability and Accountability Act (HIPAA)" and references a "Certificate of Confidentiality from the Department of Health and Human Services (DHHS)." | Yes | Data will be use in the future study without additional consent | Principal Investigator | Data will not be used and destroyed after withdrawal |
| NCT05335889 | Yes (Trial name, related institution, ethical approval) | eButton (a wearable camera) | Diagnostic support (The recorded food picture data from eButton are processed by the artificial intelligence technology to automatically determine food names, volumes, and nutrient values | NA | Indirect benefit: The data collected will inform the development of an intervention using eButton for dietary management, which may benefit future patients. | Privacy Breach due to eButton (accidental recording, device loss, human observation, other people in frame). Mentions data encryption and face blurring as mitigations. | 13.13 | 23753 | 197 | Yes | Collecting food picture data from the eButton, data from the continuous glucose monitor (CGM), survey/questionnaire data, medical record data (HbA1c, height, weight, etc.), and audio interview data | Food image data is encrypted. Data is uploaded to a "study server." The document states that identifiable private information will not be used or distributed for future research, implying secure storage | Mentions compliance with federal and state laws, specifically the Health Insurance Portability and Accountability Act (HIPAA) and the 21st Century Cures Act. | Yes | Data will not be use in the future study | Member of the research team | Data will not be used and destroyed after withdrawal |
| NCT05352399 | Yes (Trial name, related institution, ethical approval) | NeuViCare | Therapeutic Support (The "artificial intelligence+ care coach intervention" via the "NeuViCare" app aims to improve care transitions and provide support/resources for patients and caregivers after an ED visit.) | The study involves persons living with dementia. Assent is obtained from the participant, and full informed consent is required from a Next of Kin, Caregiver, or Legally Authorized Representative. | Providing access to community resources, a care plan, peer community, and educational information via the NeuViCare app to help after an ED visit. Helping researchers understand more about ED care transitions | A slight risk regarding the confidentiality of participation. Potential burden from completing interviews or workshops. | 12.67 | 7821 | 72 | NA | Collects research study records, specific medical/laboratory records related to the study, phone call records, and records of study visits. Protected Health Information (PHI) under HIPAA | Information is kept confidential. Shared with specific entities (DHHS, Yale IRB, research team) under confidentiality agreements. Covered by an NIH Certificate of Confidentiality, which provides strong legal protection against compelled disclosure. Data retention period is not specified in the provided text. | Explicitly mentions compliance with the federal "Privacy Rule of the Health Insurance Portability and Accountability Act (HIPAA)" and references a "Certificate of Confidentiality from the National Institutes of Health." | Yes | Data will be use in the future study without additional consent | Principal Investigator | NA |
| NCT05381064 | Yes (Trial name, related institution) | Deep Learning | Diagnostic support (The AI system provides real-time alerts to novice endoscopists about the current scanning site and the presence of common bile duct stones to improve diagnostic accuracy.) | NA | The AI system can assist novice endoscopists in scanning standard bile duct sites and detecting lesions, reducing dependence on the endoscopist's experience, improving examination quality, and improving the accuracy of diagnosing common bile duct stones. | No additional risks | 18.1 | 10746 | 54 | NA | During the procedure, the subject's "endoscopic electronic images and case information will be collected and preserved in the hospital" for the researcher/doctor to access for scientific research. | Data will be "preserved in the hospital" and that the research team will "do everything within the law to protect the privacy" of the subject's personal medical data. It implies secure, institutional storage but lacks specific technical details like encryption. | NA | Yes | Data will be use in the future study with additional consent | Member of the research team | NA |
| NCT05437237 | Yes (Trial name, related institution, ethical approval) | NA | Diagnostic support (Aim is to develop an algorithm to identify which patients have a large ischemic stroke in the ambulance for triage purposes.) | NA | No personal benefit for the participant. The benefit is societal/for future patients: contributing to knowledge about early stroke detection so patients can be taken directly to the right hospital in the future. | No additional risks | 12.46 | 10223 | 102 | NA | Details data collected: EEG data, medical history, medication use, physician's exam/scan results, diagnosis, treatment, ambulance transport data. States data is used to answer study questions and publish results. Mentions sharing coded data with external commercial parties for algorithm development.) | Data will be saved by the investigator for 15 years. Uses coding to protect privacy. Encryption key stored locally. Data stored on secure servers compliant with EU regulations/CE-marked equipment mentioned for EEG.) | NA | Yes | Data will not be use in the future study | Member of the research team | Data will not be used and destroyed after withdrawal |
| NCT05534178 | Yes (Trial name, related institution, ethical approval) | Machine learning | Risk prediction (The AI/ML model is intended to predict "Hospital Length of Stay (HOLS) and Mortality After Discharge") | NA | There may be no direct benefit to participation in the study. However, the identification of possible prognostic and/or predictive factors of improvement or worsening of the quality of life and survival of hospitalized patients could benefit other patients in the future and contribute to a better understanding and treatment of patients who require inpatient care. | No additional risks | 18.1 | 4318 | 23 | NA | Data will be used "in this research project," for investigating prognostic factors, and may be transmitted to third parties for the same study purposes. | Data is kept in the hospital's SAP computer system and analyzed at VHIO. Also references compliance with data protection regulations. | Explicitly references compliance with "current European and national regulations on the Protection of Personal Data" and the "Spanish Agency for Data Protection." | Yes | Data will not be use in the future study | Principal Investigator | Data will not be used and destroyed after withdrawal |
| NCT05537922 | Yes (Trial name, related institution, ethical approval) | Deep and Machine learning | Risk prediction / Patient stratification (Aims to "identify patients who will benefit from the immunotherapy treatment and possible resistance mechanisms" to "further personalize treatment") | NA | Helping physicians personalize treatment ("give the right treatment at the right time for the right patient"), contributing to future improvements in lung cancer care. | NA | 17.97 | 14521 | 73 | NA | Collection of clinical data, radiological images (CT, PET), tissue, blood, and stool samples. Data will be used to identify predictive factors and resistance mechanisms. | Data will be stored encrypted for 25 years, then anonymized. Stored under supervision of Dr. Prelaj at the institute. Transfer to other I3LUNG centers is detailed with safeguards. | Explicitly mentions and complies with EU General Data Protection Regulation (GDPR) (UE) 2016/679. Also references Italian Legislative Decree 196/2003. | Yes | Data will be use in the future study without additional consent | Member of the research team | Data already collected will continue to be used in the research. |
| NCT05558605 | Yes (Trial name, related institution, ethical approval) | NA | Diagnostic support (To assist a non-expert in acquiring echocardiogram images for identifying heart valve and function problems) | NA | More rapid and convenient exclusion of a cardiac problem because the scan can be provided locally and interpreted remotely. | Risk of missing significant findings on any echo test (including the AI-guided one); Unease from discovering a previously unknown problem; The AI device (Caption Health software) is not approved for use in Australia, implying potential unknown risks. | 13.28 | 17890 | 145 | NA | Images and personal/health information will be collected, used for the research, and may be accessed from health records. Data may be shared with other researchers in aggregated or coded form via secure repositories. | Images will be stored at the Baker Institute, labelled with a unique study number (not name). Personal information will be securely stored. Data will be stored for 15 years. Data is re-identifiable via a secure code list. | NA | Yes | Data will not be use in the future study | Member of the research team | Data already collected will continue to be used in the research. |
| NCT05619042 | Yes (Trial name, related institution, ethical approval) | NA | Diagnostic support (To develop a diagnostic method for the detection of cardiovascular disease; to train an AI algorithm to differentiate which samples carry the genetic information of the disease) | NA | Every participant will receive a written Report on the presence and extent of coronary artery calcifications, useful for cardiovascular prevention. The study will improve early detection of diseases in the future. | The document details risks of blood draw, swab, and CT scan radiation | 16.78 | 19669 | 125 | NA | Collected data includes identifying data, medical data from procedures and interviews. Purposes include research, developing diagnostics, training AI, creating reports/publications, and validating results. | Data is pseudonymized. Stored on AWS servers in the USA. RNA sent to a lab in the UK. Clinical analysis at Stamboulian Lab in Argentina. Samples may be stored in a biobank at FLENI or abroad. Center keeps data as per legal regulations. | Explicitly mentions compliance with Argentine National Law 25.326 on Personal Data Protection and the role of the Agency for Access to Public Information. | Yes | Data will be use in the future study with additional consent | Member of the research team | Data will not be used and destroyed after withdrawal |
| NCT05708846 | Yes (Trial name, related institution) | NA | NA | NA | NA | NA | 20.67 | 1115 | 7 | NA | the data collected will be used solely for the purposes of the 'study'" and that "the minimum data necessary to achieve its objectives will be collected." | Data will be stored on a secure server. | Explicitly mentions compliance with "Regulation EU 2016/679... (GDPR)" and "Organic Law 3/2018... (LOPD)". | Yes | NA | NA | NA |
| NCT05754476 | Yes (Trial name, related institution, ethical approval) | NA | Diagnositic support (Aims to use AI to predict full-dose MRI images from low-dose scans, thereby reducing contrast agent dose without affecting diagnosis) | NA | Reducing the dose of gadolinium contrast agent, reducing the risk of gadolinium exposure for patients, improving the safety of contrast agent use. | The document mentions general risks of MRI (noise, discomfort) and risks associated with the contrast agent itself | 16.9 | 10570 | 69 | NA | Clinical information and MRI images will be collected and used for the study's evaluation purposes. | Data (medical records) will be kept at the hospital. Access is granted to the research team, ethics committee, and regulatory bodies, all of whom have confidentiality obligations. Identity information will be kept confidential in any publications. | NA | Yes | NA | Member of the research team | NA |
| NCT05843682 | Yes (Trial name, related institution, ethical approval) | NA | Risk prediction (The app is used for participants to measure and transmit blood pressure data. The team uses this data to contact participants for guidance when values are abnormal. | NA | Provides important information for participants to better control their blood pressure and avoid disease complications. Facilitates virtual guidance from the multidisciplinary team. | Minimal risk. Highlights potential for data loss from the application or software update problems. Notes that alerts may not be received if there is no internet network. | 16.64 | 6435 | 39 | NA | Data collected includes home BP measurements via the app, clinical data (office BP, weight, etc.), lab results, and questionnaire responses. The app transmits BP data for team review. | States "All information obtained in this study will be of complete confidentiality, confidentiality and privacy." | NA | Yes | NA | Principal Investigator | NA |
| NCT05872945 | Yes (Trial name, related institution, ethical approval) | Machine learning | Diagnostic support (Detection of T-wave inversion in ECGs to prevent sudden death), Risk prediction (Detection of abnormal deviations in biological parameters and physical data for injury prevention and performance optimization) | NA | Optimizing health and performance of players, generating algorithms/mathematical models for better health controls and sports load management, potential for paradigm change in sports science. | NA | 19.17 | 8972 | 40 | NA | Biomedical and physiological data collected during routine practice (specifically: ECGs, blood tests, urine tests, sports geolocation data, DEXA, MRI, rx, CT scans, functional assessments) will be processed for research purposes to generate algorithms or mathematical models. | Data will remain anonymous. Storage details are not specified in the consent form, but the main document mentions data will be processed disassociated from identity and comply with data protection laws. | Explicitly mentions compliance with the General Data Protection Regulation (GDPR - "RGPD" in the text) and Organic Law 15/1999 on Data Protection. | Yes | NA | NA | Data will not be used and destroyed after withdrawal |
| NCT05967260 | Yes (Trial name, related institution, ethical approval) | NA | Risk prediction and therapeutic support (The "DailyDose" app tracks sensor glucose and makes personalized snack recommendations at bedtime to prevent low glucose while participant's sleep.) | Specific protection for pregnant individuals: exclusion from the study, mandatory pregnancy tests, requirement for effective birth control. A Certificate of Confidentiality is obtained to protect participant privacy from court orders. | The research may benefit in the future people with type 1 diabetes by helping to develop better management tools for diabetes. (No direct benefit to participant is guaranteed.) | Risk of high or low glucose | 12.19 | 27393 | 241 | Yes | Data collected includes: glucose sensor data, sleep data from Apple Watch, self-logged snacks/exercise, survey responses, vitals, medical history, lab results. Data may be shared with NIH, FDA, Dexcom, OHRP, and stored in a repository for future research. | Data is stored on a secure OHSU-approved cloud database repository. Identifying information is removed before sharing with outside researchers. Data is linked to a 3-digit ID. The key linking ID to personal info is encrypted on a restricted OHSU drive. Data is stored indefinitely until the PI decides to discontinue the repository. | Mentions compliance with federal law (HIPAA), Oregon law, FDA regulations, and Medicare reporting requirements. References the Oregon Tort Claims Act. | Yes | Data will be use in the future study without additional consent | Member of the research team | Data will not be used and destroyed after withdrawal |
| NCT06017089 | Yes (Trial name, related institution, ethical approval) | Automated initialization (AI) program | Therapeutic support (The AI program suggests insulin pump settings for the study doctor to review, aiming to help manage the child's type 1 diabetes.) | The study involves young children (ages 2-6) who are vulnerable. Consent is provided by a parent/LAR. The AI does not control the pump directly; all suggestions must be reviewed and entered by the study doctor, providing a human safeguard. The study team will contact the child's personal doctor for safety verification. | Possible benefits are a better understanding of the child’s diabetes or a positive impact on the family’s ability to manage it. The information gained may help others with type 1 diabetes in the future. | The AI program might suggest pump settings that cause too little or too much insulin to be delivered. | 11.56 | 37182 | 329 | Yes | Data collected includes contact information, medical/diabetes history, height/weight, HbA1c levels, and continuous data uploaded from the insulin pump and CGM via the t:connect Mobile App. | Data is stored at the Jaeb Center for Health Research (JCHR) in Tampa, FL, using a coded system. Companies involved (UVA, Tandem, Dexcom) and monitoring agencies (FDA) may also access the data, sometimes in coded form, sometimes with identifiers like date of birth. Data is protected under a Certificate of Confidentiality. | Mentions FDA approval/oversight and a Certificate of Confidentiality from NIDDK. | Yes | Data will be use in the future study without additional consent | Member of the research team | Data already collected will continue to be used in the research. |
| NCT06240234 | Yes (Trial name, related institution, ethical approval) | NA | Risk prediction (To predict and avoid blood pressure drops during surgery) | NA | Early warning system to allow preventive treatment, potentially reducing the risk of blood pressure drops and subsequent post-surgical complications. | No additional risks | 14.25 | 6421 | 55 | NA | Collecting blood pressure, ECG, oxygen saturation, other standard monitoring data, and blood samples (1-2ml/hour) for oxygen and other parameters. Basic medical history and lab test data will also be obtained from hospital records. | Data will be retrieved from the hospital's encrypted storage, saved in an anonymized form, and stored by study managers for up to 10 years. | Explicitly mentions compliance with the EU's General Data Protection Regulation (GDPR). | Yes | NA | Member of the research team | Data will not be used and destroyed after withdrawal |
| NCT06240897 | Yes (Trial name, related institution, ethical approval) | NA | Diagnostic support (Management tool for delirium) | The study targets patients aged 65+, a potentially vulnerable group. Protections include using unique codes for data, storing the code-to-identity link securely, and allowing withdrawal at any time without affecting medical care. For incidental findings, participants can choose whether to be notified. | Potential to add to medical knowledge about using this digital intervention for delirium. | Risk of data breach leading to loss of privacy/confidentiality. | 14.2 | 11553 | 95 | NA | Collecting deidentified game play data, information from medical records (e.g., diagnosis of cognitive impairment/delirium), and data from questionnaires (demographics, daily cognitive assessments, game feedback). | Data stored in Singapore. Personal data is labelled with a unique code. The link between code and identity is kept in a separate, secure file with restricted access. Data is the property of SingHealth and used only for this study. | NA | Yes | NA | Principal Investigator | Data already collected will continue to be used in the research. |
| NCT06253065 | Yes (Trial name, related institution, ethical approval) | Deep learning | Diagnostic support (AI system interprets digital pathology images to assist pathologists in detecting lymph node metastases, particularly micrometastases, to enhance diagnostic accuracy and efficiency) | NA | Potential to mitigate the risk of misdiagnosing micrometastases, thereby enhancing diagnostic accuracy and efficiency. Contributes to further research and understanding of the disease, potentially fostering future improvements in treatment. | No additional risks | 15.98 | 8510 | 60 | NA | Collecting clinical information (age, PSA levels, treatment history) from hospital records and digital pathology images from lymph node slides. | Data confidentiality and protection "in accordance with the law," | NA | Yes | Data will be use in the future study without additional consent | Member of the research team | NA |
| NCT06357039 | Yes (Trial name, related institution) | NA | NA | NA | NA | NA | 17.79 | 3869 | 23 | NA | The results of the study may be presented in scientific meetings or publications. However, in such cases, my identity will be kept strictly confidential. | NA | NA | Yes | NA | Member of the research team | NA |
| NCT06421324 | Yes (Trial name, related institution, ethical approval) | NA | Risk prediction (Aims to classify patients into risk levels and provide personalized health recommendations for gastric cancer prevention) | NA | Possible that will not gain any benefit to participant's health from participating in this study, but it may help to better understand the disease and improve the prognosis and treatment of future patients. | No additional risks | 16.71 | 16934 | 101 | Yes | Details collection from questionnaires, clinical records, and tests; states data will be encrypted/coded, shared with the research team, and used for publications | Data will be kept under "adequate security conditions"; lists co-responsible institutions; mentions storage in INCLIVA Biobank for future research with committee approval+M34 | Explicitly references EU GDPR, Spanish Law 14/2007 on Biomedical Research, Royal Decree 1716/2011 on Biobanks, and the Spanish Data Protection Agency | Yes | Data will be use in the future study with additional consent | Principal Investigator | Data already collected will continue to be used in the research. |
| NCT06435286 | Yes (Trial name, related institution, ethical approval) | Artificial intelligence-based mobile high-resolution microendoscope (AI-mHRME) | Diagnostic support (Identifies abnormal cells to assist doctors in finding unusual cells in the esophagus, interprets HRME images to aid diagnosis) | Exclusion of pregnant patients (via mandatory urine test), privacy protection through de-identification and secure storage. | No direct benefit to participant. Potential future benefit: More effective methods for detecting esophageal cancer, advancing understanding, improving healthcare practices. | Disclosed procedural risks: Allergic reaction to dye, risks from prolonged anesthesia, aspiration, loss of privacy. | 14.8 | 24728 | 180 | NA | Collects health information (diagnoses, notes, labs, demographics, photos/videos), esophageal descriptions, doctor interpretations, biopsy sites. Used/disclosed to researchers, IRB, BCM/BSLMC/Ben Taub, NCI, NIH, FDA, HHS, Data Coordinating Center, DSMB. | Data stored securely. PHI stored securely. Data coded and de-identified using study ID. | Mentions U.S. Law requiring ClinicalTrials.gov posting, FDA regulations for IDE/IND, HIPAA compliance. | Yes | Data will not be use in the future study | Principal Investigator | Data already collected will continue to be used in the research. |
| NCT06455111 | Yes (Trial name, related institution, ethical approval) | NA | NA | NA | NA | NA | 13.46 | 1223 | 11 | NA | Collecting survey responses on CGM report interpretations. Name/email collected only if participant wants authorship, stored separately from survey responses. | Collecting survey responses on CGM report interpretations. Name/email collected only if participant wants authorship, stored separately from survey responses. | NA | NA | NA | Member of the research team | NA |
| NCT06474338 | Yes (Trial name, related institution) | NA | NA | States that personal information will be hidden in publications to protect privacy. Mentions that government/ethics committees may review data to ensure regulations are followed and rights are not compromised. Patient information will not be disclosed to irrelevant individuals without permission | NA | NA | 17.64 | 1641 | 11 | NA | Data to be collected: personal demographic information, medical records, laboratory test data, imaging, photographs, and surgical videos. States data will be used for clinical diagnosis/treatment, teaching, and medical research | Only mentioned protection in publications/reports. | NA | Yes | NA | NA | NA |
| NCT06644248 | Yes (Trial name, related institution, ethical approval) | Deep learning | Diagnostic support (Aims to automatically detect cervical cancer or precancerous stages using colposcopy images.) | Excludes pregnant women and those with severe medical conditions. Uses unique IDs and removes personally identifiable information to protect privacy | Do not guarantee or promise that will receive any benefits from this study. The study is being conducted for humanitarian purposes, which envision a long-term goal that will benefit patients suffering from or at the risk of having cervical cancer. | Mentions mild discomfort, minimal bleeding, or infection from the colposcopy/biopsy procedure itself, but does not disclose risks specific to the AI | 14.8 | 4885 | 38 | NA | Data (clinical info, colposcopic images, HPV status, medical history) will be used to train the AI algorithm for automatic classification and detection | Video is stored encrypted on a password-protected computer at the facility and transmitted via encrypted channel to SafelyYou. Data is stored on a password-protected, HIPAA-compliant AWS website. Data is retained for 5 years post-study. | NA | Yes | Data will be use in the future study with additional consent | Principal Investigator | Data will not be used and destroyed after withdrawal |
| NCT06652061 | Yes (Trial name, related institution, ethical approval) | Deep Learning, Convolutional Neural Networks (CNNs) | Diagnostic support (To predict Bone Mineral Density (BMD) from X-ray images to aid in the early detection of osteoporosis and osteopenia, and to serve as a clinical decision support tool for determining the necessity of DEXA scans.) | NA | Potential long-term humanitarian benefits for patients at risk of osteoporosis by enabling earlier detection and management, particularly in resource-limited settings like Bangladesh where DEXA scans are scarce. Aims to provide a cost-effective screening tool. | Radiation risk inherent in any radiographic test, not specific AI risks | 14.34 | 4376 | 38 | NA | Data collected includes X-ray images, DEXA scan results, age, gender, phone number, BMI, menopausal status, diabetes, and cardiovascular disease (Page 13). Data will be used to train and evaluate an AI algorithm for automatic BMD prediction (Page 13, 14). | Data will be stored in a database at the Department of BME, BUET (Page 14). The document states safety measures will be used to protect privacy but does not detail the specific storage infrastructure (e.g., encrypted servers, cloud provider). | The justification for the retrospective consent waiver (Page 18) explicitly mentions and cites guidelines from the U.S. Department of Health and Human Services (HHS 45 C.F.R. § 46.116(d)) and the Bangladesh Medical Research Council (BMRC) "Ethical Guidelines for Conducting Research Studies Involving Human Subjects". | Yes | Data will be use in the future study with additional consent | Principal Investigator | Data will not be used and destroyed after withdrawal |
| NCT06652854 | Yes (Trial name, related institution, ethical approval) | Large Language Model (specifically ChatGPT) | Therapeutic Support (To assess how well ChatGPT answers patient questions compared to responses provided by anesthesiologists during pre-anesthesia consultations.) | NA | May not receive direct benefit from participating in this study. However, we hope the information learned will help improve perioperative education for future patients. | Time commitment, emotional discomfort from comparison, privacy concerns | 14.15 | 11180 | 95 | NA | Data will be used "in analyses and will be published/presented to the scientific community." It also mentions data may be used for "future research on the integration of AI technologies in clinical practice." | The form states that authorized representatives may access records at the site and that data sent to organizations will be de-identified (using a participant code, sex/gender, and age). It mentions compliance with privacy laws but does not specify the physical or digital storage platform | The consent form states, participant's rights to privacy are legally protected by federal and provincial laws" and that data transfer will be in compliance with all relevant Canadian privacy laws. | Yes | Data will be use in the future study without additional consent | Principal Investigator | Data will not be used and destroyed after withdrawal |
| NCT06712160 | Yes (Trial name, related institution, ethical approval) | NA | Diagnostic support (Detecting the number of root canals in molars) | NA | Reducing diagnostic errors, reducing treatment time, increasing accuracy, enhancing treatment success rate by eliminating human error. | NA | 15.82 | 5839 | 33 | NA | Two-dimensional and three-dimensional (CBCT) X-rays/images will be taken and used for comparison with the AI program and the clinician's findings. Photographs or videos of teeth (not face) may be taken for documentation, educational, or research purposes. | NA | NA | Yes | NA | Member of the research team | NA |
| NCT06713122 | Yes (Trial name, related institution, ethical approval) | NA | Therapeutic Support (AI is used to help patients "reach the right information without wasting time" for symptom management during chemotherapy. It is not described for diagnosis or risk prediction) | NA | AI integration will help patients access correct information quickly. It also mentions that expert-created content within the app aims to positively affect supportive care needs, symptom severity, and psychological well-being, contributing to a "more harmonious and healthy treatment process." | No additional risks | 15.7 | 9442 | 59 | NA | Data collection tools (various scales/forms) and states they will be sent via the application and filled in by the patient at three specific time points. | NA | NA | Yes | Data will be use in the future study with additional consent | Member of the research team | NA |
| NCT06717984 | Yes (Trial name, related institution) | NA | Diagnostic Support (Classification). The stated purpose is to develop an AI algorithm that will "automatically detect invasive and non-invasive cancer types using histopathological images" to aid in diagnosis and serve as a "clinical decision support tool." | NA | Study do not guarantee or promise that participants will receive any benefits from this study. The study is being conducted for humanitarian purposes, which envision a long-term goal that will benefit patients suffering from or at the risk of having breast cancer | There may be mild discomfort during the procedure and, in rare cases, minimal bleeding or infection at the biopsy site | 15.9 | 4819 | 33 | NA | Data (histopathology images and metadata) will be collected, anonymized, encrypted, and put into a database at BUET's Department of BME for the purpose of developing the AI algorithm and for "future research." | Data will be stored in "one of the databases of the Department of BME, BUET." It also states data will be encrypted and anonymized | The "Justifications for Informed Consent Waiver" section explicitly references and quotes government/official policies: the U.S. Department of Health and Human Services (HHS) regulations (45 C.F.R. § 46.116(d)) and the Bangladesh Medical Research Council (BMRC) "Ethical Guidelines." | Yes | Data will be use in the future study with additional consent | Principal Investigator | Data will not be used and destroyed after withdrawal |
| NCT06815939 | Yes (Trial name, related institution, ethical approval) | (Automated Visual Evaluation (AVE) (Deep learning) | Diagnostic support (The AVE's role is to "analyze a digital image of the cervix to detect pre-cancer" and to help "identify cervical cancer and pre-cancer through this technology." Its purpose is to serve as a screening test.) | NA | Improved access and timeliness. The primary benefit stated is that if effective, the AVE test "can help us detect pre-cancer or cancer in women without waiting for results to come back from a laboratory and will allow more women to have access to prevention and appropriate treatment if needed." It also notes participants have a higher chance of detection due to multiple tests being performed. | Participants may feel discomfort or feel embarrassed about the speculum that will be placed in their vagina. | 12.97 | 18576 | 165 | NA | Data will be analyzed by researchers. De-identified data may be shared with other researchers or used for future, unspecified research projects. Results will be published in journals/conferences without identifiers. Cervical images will be used to train healthcare providers and to "build or improve computer programs such as AVE." | De-identified data will be stored in "secured files and password-protected databases." Cervical images will be stored in a "virtual file managed by DL Analytics" that is "only accessible to researchers of this study and authorized experts from DL Analytics." Identifying information will be kept secure and later destroyed or de-identified. | The document mentions compliance with a "Certificate of Confidentiality from the United States National Institutes of Health." It also provides contact information for the Salvadoran "National Committee of Ethics in Health Research" and the "National Directorate of Medicines," indicating adherence to national regulatory frameworks. | Yes | Data will be use in the future study without additional consent | Principal Investigator | Data will not be used and destroyed after withdrawal |
| NCT06842927 | Yes (Trial name, related institution, ethical approval) | NA | Diagnostic support (To predict dialysis adequacy and peritoneal membrane transporter status) | NA | May improve future care for PD patients by providing more accurate monitoring and better treatment options | No additional risks | 14.74 | 7956 | 64 | NA | Collecting an extra peritoneal dialysate sample and a urine sample; data will be used to help develop the AI model. | Data stored securely on computers accessible only by researchers. Signed consent stored separately. Data will be destroyed 7 years after study completion. Access granted to researchers, REC, and regulatory authorities. | Explicitly mentions compliance with Hong Kong's Personal Data (Privacy) Ordinance, Cap 486, and directs participants to the Privacy Commissioner.) | Yes | NA | Member of the research team | Data will not be used and destroyed after withdrawal |
| NCT06858553 | Yes (Trial name, related institution, ethical approval) | Deep learning | Diagnostic support (To characterize intestinal fibrosis in Crohn’s disease using MR enterography) | NA | Participation in this study does not provide direct personal benefits or improvements in disease prognosis. However, researchers hope this research will contribute to medical advancements, leading to improved diagnostic tools and treatment methods for a broader patient population. | May experience allergic reactions to the contrast agent used in MRE. | 16.59 | 6291 | 48 | NA | Collects clinical baseline information, serum and fecal test indicators, MRE imaging data, and surgical specimens (five tissue samples per patient). Data obtained from routine clinical processes. | Data will be strictly confidential. Surgical specimens labeled with study ID. Records securely stored, accessible only to authorized personnel. Data may be reviewed by government authorities or ethics committee. If transferred internationally, protective measures will be implemented. Paraffin-embedded tissue sections stored at treatment centers. | Explicitly mentions compliance with the Personal Information Protection Law, Cybersecurity Law, and Data Security Law of the People's Republic of China. | Yes | Data will not be use in the future study | Member of the research team | NA |
| NCT06859216 | Yes (Trial name, related institution, ethical approval) | AI-Generated Plain Language Summaries | Therapeutic Support (The AI generates summaries to help patients better understand their medical notes and treatment plans.) | NA | The AI-generated summary may help clarify information about participant eye health and treatment plan. The research aims to improve doctor-patient communication, potentially leading to better health outcomes and satisfaction with care. | No additional risks | 16.14 | 9265 | 50 | NA | Data collected include participants responses to the surveys about the medical notes and the notes themselves (linked via a unique study ID). De-identified data may be used for future research. | Data will be securely stored on an encrypted UCLA server, accessible only to authorized personnel. Physical records will be kept in locked cabinets. Data will be maintained for at least three years. | NA | Yes | Data will be use in the future study without additional consent | Member of the research team | NA |
| NCT06904586 | Yes (Trial name, related institution, ethical approval) | Machine learning | Risk prediction ((The AI's role is to predict if a patient has a "Difficult Airway" or "Difficult Intubation" based on their physical measurements, to allow for precautions to be taken.) ) | NA | Providing "preliminary information" for future situations like surgery or accidents if the participant has a difficult airway, ensuring necessary precautions can be taken | NA | 16.34 | 4498 | 22 | Yes | The measurements that can be obtained from pariticipants will be used for scientific purposes and that their medical data may also be used for scientific purposes if the participant withdraws. It specifies analyses will not be conducted abroad. | All participant medical and identity information will be kept confidential... however, the audience of the study, those conducting the examination, ethics committees and official authorities may access their medical information when necessary." | NA | Yes | Data will be use in the future study with additional consent | Member of the research team | Data already collected will continue to be used in the research. |
| NCT06989255 | Yes (Trial name, related institution, ethical approval) | NA | Diagnostic support (Aims to achieve "automated detection of deep vein thrombosis on conventional ultrasound scans") | NA | No guaranteed direct medical benefits to participate in this study. However, participants may benefit by assisting the study research team to understand better of their condition. | No additional risks | 13.37 | 11506 | 104 | NA | Demographic data, medical/pharmaceutical information, images that we collect during the study | Give participants a code, and only the authorised study personnel could match that code with participant's identity. This code is only for participant. We keep the key to the code in a safe place in the hospital. When researchers process their data, we always use only that code. Even in reports and publications about the study, nobody will be able to understand that it is participant behind this code. | EU regulation 2016/679 GDPR | Yes | NA | NA | Data will not be used and destroyed after withdrawal |
| NCT07000721 | Yes (Trial name, related institution) | NA | NA | NA | NA | NA | 8.52 | 2591 | 14 | NA | NA | NA | NA | Yes | Data will be use in the future study with additional consent | NA | NA |
| NCT07108452 | Yes (Trial name, related institution, ethical approval) | Mobile app (PlantVillage Food works) | Therapeutic Support (Promote healthy eating) | NA | Participant won’t get any personal health benefits from joining. But the research could help us learn moreabout how mobile apps and AI can support healthy eating. | Only risk mentioned is potential discomfort from survey questions | 11.26 | 3724 | 42 | NA | Data will be used for this study and may be used for future research by this or other institutions, including commercial entities, and placed in external databases. Personally identifiable information will not be shared. | Data will be coded and stored in a password-protected Box folder accessible only by the PI and project manager. | NA | Yes | Data will be use in the future study with additional consent | Principal Investigator | NA |
| NCT04473326 | Yes (Trial name, related institution, ethical approval) | Reinforcement learning | Therapeutic support AI system (Via electronic pill bottle + texts) aims to improve medication adherence through adaptive reminders | NA | No any direct benefit from taking part in this study | Using electronic pill bottles during this study could change participant's daily medication-taking routine, text messages are not encrypted, and therefore carry security risks. | 12.69 | 21356 | 176 | NA | Paticipant's name, medical record number, date of birth, Past, present, and future medical records, Research procedures, including research office visits, tests, interviews, and questionnaires | Pillsy data stored on their servers bys data storage companies, accreditors, insurers, and lawyers | Explicitly mentionsA federal Certificate of Confidentiality to protect participant's privacy | Yes | NA | Principal Investigator | Data will not be used and destroyed after withdrawal |
| NCT05369806 | Yes (Trial name, related institution, ethical approval) | NA | Risk prediction (Help nurses prioritize messages sent by participants so they respond to the messages needing urgent responses first.) | When assess participant's mental well-being, if identity that participants are at risk of self-harm or have depression, researcher will disclose this information to participants also refer to a qualified medical provider who can help participants with their mental health and disclose that information to the medical provider | Participants may personally benefit from receiving information and advice about delivery planning, encouragement and counseling about assessment of and advice about their infant’s health. Personally benefit from being able to ask questions toa nurse about participant's infant’s health, family planning and labor and delivery | There is a small risk that participant's someone outside of the study could gain access to participant's information. If this happened, it could be embarrassing, cause stress and discomfort and even cause problems at home.   May experience stress or discomfort if a partner learns of the study participation. There is risk that this disclosure could result in psychological harm or even physical harm  May become embarrassed or worried when we ask personal questions about participant or participant's infant. | 12.9 | 15930 | 132 | NA | Collects SMS message content, responses, contact info, locator info, survey responses, and accesses maternal/infant medical records for research purposes | Medical information that share will be identified by a code number. All of information, including the link between participant's name and code number will be kept in a secure location.Once the study is completed, the link between participant's identifier and research data will be destroyed after the records retention period by law. | Explicitly mentions a Certificate of Confidentiality from the U.S. National Institutes of Health; notes compliance with U.S. law | Yes | Data will be use in the future study without additional consent | Principal Investigator | Data already collected will continue to be used in the research. |
| NCT07136207 | Yes (Trial name, related institution, ethical approval) | Facial action units | Diagnostic support (Automatically identifies emotional states and builds a facial expression–based delirium recognition model for neurocritical patients) | Age under 18 years;Persistent coma (GCS≤ 8) within 7 days before and after surgery, making delirium assessment impossible; Severe dementia, cognitive impairment, or psychiatric disorders that prevent deliriumevaluation; Surviving less than 24 hours in the ICU; Patients with facial paralysis, post-traumatic facial damage, or other conditions that may severely impair facial recognition; Patients with dementia, Parkinson's disease, depression, or other conditions affecting facial emotional expression are exclude to the study | Help determine which treatment is safer and more effective for other patients with similar conditions. | May potentially infringe upon patients' right to portrait and privacy | 16,4 | 11551 | 81 | NA | Collects facial video during CAM-ICU–based cognitive/attention tasks; records correctness of answers and facial expressions; used to extract AUs and build recognition model | Medical records will be kept at the hospital, any public reports on the results of this studywill not disclose participant's personal identity. We will make every effort to protect the privacy of participant's personal medical information within the scope permitted by law | NA | Yes | NA | Member of the research team | NA |
| NCT06008548 | Yes (Trial name, related institution, ethical approval) | Machine learning | Risk prediction (Guide patients tochoose hepatic resection treatment or hepatic artery chemotherapy tethering therapy) | NA | Personalized treatment plans for liver cancer in the future | No additional risks | 16.57 | 8078 | 45 | NA | Age, gender, etiology, blood biochemistry and tumour load will be collected for this study. | Personal information of research subjects will be kept strictly confidential and participant's personal information will not be disclosed except as required by relevant legislation. | NA | Yes | NA | Member of the research team | NA |
| NCT07102810 | Yes (Trial name, related institution, ethical approval) | Machine learning | Risk prediction (Characterize physiological biomarkers of calm and tranced states ) | NA | No direct benefits but involvement may contribute to future tools that help reduce anxiety without medication | A little emotional, sleepy, or more sensitive to suggestions, might bring up old memories or unexpected feelings | 12.91 | 16867 | 137 | NA | Collect data about participant's body during a hypnosis session ( brainwaves, heart rate, breathing, and skin moisture) | Video will be collected and stored/analyzed in secure environments. | Mentioned HIPAA, U.S. law (ClinicalTrials.gov posting), DHHS, OHRP, FDA access | Yes | Data will not be use in the future study | Member of the research team | Data already collected will continue to be used in the research. |
| NCT04870099 | Yes (Trial name, related institution, ethical approval) | Natural Language Processing (NLP) | Therapeutic support (NLP used to explore whether social media data can predict or track engagement with a guided self-help CBT book for depression/anxiety) | NA | Possible reduction in anxiety, stress, or depression symptoms | There is a risk that the information provided becomes compromised and is no longer confidential and a risk that while completing the survey questions may be uncomfortable answering some of the questions | 12.67 | 10505 | 91 | NA | Collects baseline/follow-up survey data, Twitter handle, and social media data including post frequency, timing, thinking styles, and affect | All research interactions will occur on secure platforms, and participant's data will be kept in a secure location. Information collected from their Twitter account will be assigned an anonymous ID | This research is covered by a Certificate of Confidentiality from the National Institutes of Health | Yes | Data will be use in the future study without additional consent | Member of the research team | NA |
| NCT05819151 | Yes (Trial name, related institution, ethical approval) | Machine learning | Diagnostic support (To estimate HbA1c levels and detect diabetes non-invasively using tongue images and symptoms) | NA | No immediate clinical benefit, but may help develop non-invasive diabetic screening methods | It is very unusual that participant may have emotional fluctuation during completing the questionnaire and study procedures | 13.47 | 10522 | 84 | NA | Tongue images, HbA1c/Hb lab results, and DTQ symptom questionnaire collected for machine learning | Stored on School of Chinese Medicine of HKBU computer; paper questionnaires kept 3 years; digital data kept 7 years post-publication; Qualtrics data deleted after project | Explicitly cites Hong Kong’s Personal Data (Privacy) Ordinance, Cap 486; directs participants to Privacy Commissioner | Yes | NA | Principal Investigator | Participants may choose to allow their collected data to remain in the study or destoryed |
| NCT06400277 | Yes (Trial name, related institution) | NA | Therapeutic support (Compare three different traditional and three different digital impression methods) | NA | No direct benefit | No additional risks | 14.27 | 7462 | 62 | NA | NA | NA | NA | Yes | NA | Member of the research team | NA |
| NCT07112599 | Yes (Trial name, related institution) | NA | Risk prediction (Predicting pelvic lymph node metastasis pre-surgery); Diagnostic support (diagnosing lymph node metastasis, invasion, grade, subtype, and probability of biochemical recurrence) | NA | Accurate prediction of lymph node metastasis to avoid unnecessary pelvic lymph node dissection and its complications; more personalized treatment plans; may provide useful information for participant’s treatment or disease research | There may be some very small risks during specimen collection, including brief pain, local bruising, mild dizziness in a few people, or extremely rare needle infection. | 16.84 | 6101 | 38 | NA | MRI images and pathology slides collected | Biological specimens and data stored at The First Affiliated Hospital of Anhui Medical University | NA | Yes | NA | Principal Investigator | Data will not be used and destroyed after withdrawal |
| NCT04378660 | Yes (Trial name, related institution, ethical approval) | Computer-Aided Detection (CADe) tool | Diagnostic support (Real-time polyp detection during standard colonoscopy as a "second observer") | NA | Potential for better polyp detection rate compared to standard colonoscopy alone | No additional risks | 16.32 | 14691 | 88 | NA | Data collected includes: demographic data, medical history, medication, diet, addictions, colonoscopy video, AI detection counts/categorizations (true positive, false negative, etc.), and histological results. | Data is encoded (anonymized with ID code) before being sent to Dept. of Gastroenterology, UZ Leuven. Stored on secure servers. Encoded data may be transferred internationally. Retained for research validity even if participant withdraws | Me+M61ntions compliance with European General Data Protection Regulation (GDPR) and Belgian Law of 7 May 2004 related to experiments on humans | Yes | Data will be use in the future study with additional consent | Member of the research team | Data already collected will continue to be used in the research. |
| NCT05671601 | Yes (Trial name, related institution) | Deep learning | Diagnostic support (AI system automatically evaluates embryo development stages—prokaryotic, mitotic, blastocyst—to select optimal blastocyst for transfer and improve pregnancy outcomes) | NA | May contribute to better pregnancy outcomes through more accurate embryo selection; helps promote single blastocyst transfer, reducing multiple pregnancy risks | No additional risks | 20.39 | 13920 | 48 | NA | Collects embryo images (prokaryotic, mitotic, blastocyst stages), birth date, gender, race, physical/mental health status; data used for clinical research, marketing applications, and new embryo evaluation methods; may be transferred internationally | Data stored at Gulou Hospital (Nanjing University); files kept in locked cabinets; access restricted to researchers; government, sponsor, and ethics committee may access data for oversight; identity not revealed in publications | NA | Yes | NA | Member of the research team | Data already collected will continue to be used in the research. |
| NCT03482466 | Yes (Trial name, related institution, ethical approval) | NA | Therapeutic support (Real-time neurofeedback training: brain activity controls threat level in virtual reality to help veterans learn to regulate PTSD symptoms) | Study involves veterans with PTSD (potentially vulnerable); excludes those with metallic implants; allows immediate withdrawal if claustrophobic or distressed; mental health risks addressed via exposure therapy protocols | No direct benefit to participant; potential long-term benefit: improved understanding of PTSD brain mechanisms leading to better treatments | No additional risks | 13.89 | 11062 | 84 | NA | Collects brain MRI/fMRI images, EEG data, PTSD symptom scores, demographic info, health records (diagnoses, meds, substance use), and virtual environment interaction data | Data stored in a **secure VA repository**; identifiers removed and kept separately; only study team can re-identify via code; data will **not be shared with external investigators** | NA | Yes | NA | Principal Investigator | NA |
| NCT04507360 | Yes (Trial name, related institution) | NA | Therapeutic support (Resources aim to “help people overcome negative thinking and support healthier thought processes”) | NA | May learn strategies to manage negative thinking; no direct benefit guaranteed | Content from resources might be upsetting | 12.77 | 5953 | 52 | NA | Collects name, email, phone number (stored separately); resource usage data and survey responses (de-identified); may be used for future research or shared with external investigators if de-identified | Identifiable info stored separately from study data; data kept confidential within UW and Mental Health America research groups; de-identified data may be shared externally | NA | Yes | Data will be use in the future study without additional consent | Principal Investigator | NA |
| NCT06637774 | Yes (Trial name, related institution) | NA | Diagnostic support(Aims to detect swallowing difficulties and aspiration without X-ray, by analyzing throat vibration/sound signals during swallowing) | Study involves hospitalized patients with suspected dysphagia (potentially vulnerable); consent is voluntary; skin irritation from surgical tape noted; participant can stop if fatigued/frustrated | Development of a noninvasive, accurate screening tool to identify aspiration risk and reduce pneumonia | NA | 9.8 | 12674 | 74 | Yes | Collects throat vibration/sound data during water swallows and VFSS X-ray; also collects age, sex, and de-identified X-ray results for comparison with sensor output | Data identified by code; name/code link kept separate; data stored securely; no specific details on server type, encryption, or retention period | NA | Yes | NA | Principal Investigator | Data already collected will continue to be used in the research. |
| NCT05554042 | Yes (Trial name, related institution, ethical approval) | NA | Diagnostic support (AI analyzes voice recordings to predict risk of depression and anxiety using voice biomarkers) | if clinician believes participant is at imminent risk to self or others, steps will be taken (e.g., call 911); provides list of mental health resources to all participants | Reduction in stress, learning more about mental health | Potential for breach of confidentiality despite encryption | 13.65 | 10715 | 97 | NA | Collects name, phone, email, voice/video recordings of SCID-5 interview, demographic info, emergency contact, medical history, and SCID-5 results; de-identified data may be used for future Kintsugi AI research | Identifiable data stored in secure, password-protected REDCap Cloud and Google Cloud Platform; kept for 7 years; de-identified data kept indefinitely | NA | Yes | Data will be use in the future study with additional consent | Member of the research team | Data will not be used and destroyed after withdrawal |
| NCT05042063 | Yes (Trial name, related institution, ethical approval) | NA | Diagnostic support (AI analyzes cough sounds to objectively monitor frequency/nature of coughs for respiratory disease assessment and treatment response) | NA | Indirect benefit: May help physicians better understand disease progression or treatment response; gives patients greater control over their condition | Potential privacy breach if sound snippets are linked with metadata (e.g., phone number) | 14.96 | 16895 | 118 | NA | Collects cough sound snippets (0.5s), geolocation, time stamp, daily cough intensity (VAS), quality-of-life questionnaire responses, and medical record data (labs, microbiology, etc.) | Data codified; stored on password-protected servers/computers accessible only to authorized researchers; MP3s not shared externally; data controller complies with Spanish data protection law | Explicitly mentions compliance with Organic Law 3/2018 (Spain) and other Spanish data protection regulations; notes data may be transferred outside EU with safeguards | Yes | NA | Principal Investigator | Participants may choose to allow their collected data to remain in the study or destoryed |
| NCT07183111 | Yes (Trial name, related institution, ethical approval) | NA | Therapeutic / Educational Support(AI provides post-braces care instructions and serves as a remote support system to improve patient education, engagement, and oral hygiene) | NA | NA | NA | 26.45 | 2953 | 28 | NA | Collects oral hygiene indices (Silness Loe plaque index, Modified gingival index), patient satisfaction/engagement questionnaire responses, and recall quiz scores at multiple timepoints (T0–T3) | States “all data will be secured and is covered by Data Protection act”; no technical details on encryption, server type, or retention period | Mentions compliance with “Data Protection act” (likely referencing Pakistan’s Personal Data Protection Bill or institutional policy) | Yes | NA | NA | NA |
| NCT05863494 | Yes (Trial name, related institution, ethical approval) | Deep learning | Diagnostic support / Risk prediction (AI model uses EEG and behavioral data to predict pain levels in older adults with cognitive impairment who cannot self-report) | Exclusion include MMSE <18; participant may stop at any time if distressed | No direct benefit to participant | A small chance of a breach of confidentiality. | 13.11 | 11450 | 99 | NA | Collects demographic info, pain medication use, FACES/VAS pain scores, Mini Mental State Exam (MMSE) results, and EEG data during pain-inducing tasks | Data stored securely by University of Arizona; no specific details on encryption, server type, or retention period; identifiers kept separate from research data | NA | Yes | NA | Principal Investigator | Data already collected will continue to be used in the research. |
| NCT04614376 | Yes (Trial name, related institution, ethical approval) | Machine Learning | Risk prediction / Diagnostic support(Study aims to use continuous glucose monitoring data for early detection of Mild Cognitive Impairment and Alzheimer’s disease) | Study involves adults with Mild Cognitive Impairment or Alzheimer’s(vulnerable group); requiresdual consentfrom participant and primary caregiver; caregiver may withdraw participant at any time | Potential benefit of early detection and improved disease management through glucose pattern analysis | NA | 15.39 | 4687 | 38 | NA | Collects continuous glucose monitoring (CGM) data from existing device use; no biospecimens or additional health records mentioned | All data will be stored on a secure and compliant database with restricted access | NA | Yes | NA | Principal Investigator | Participants may choose to allow their collected data to remain in the study or destoryed |
| NCT06065319 | Yes (Trial name, related institution, ethical approval) | NA | Risk prediction / Therapeutic support(Study uses sensor data—motion, heart rate, breathing—to estimate recovery during cardiac rehab in real time; implies algorithmic modeling, though not labeled as AI) | NA | No direct benefit to participant; potential future benefit: improved cardiac rehabilitation programs through better real-time recovery estimation | Risk of loss of confidentiality | 12.76 | 16129 | 152 | NA | Collects sensor data (motion, heart rate, breathing rate) during exercise and rest, 6-minute walk test results, and medically relevant findings (if any); data may be used for future research | Identifiable information will be deleted from research data; data retained for at least 3 years; stored securely (no technical details on encryption or server type) | MentionsCertificate of Confidentialityfrom NIH; notes data may be disclosed toFDAorU.S. Governmentfor audit/evaluation of federally funded projects | Yes | Data will be use in the future study without additional consent | Principal Investigator | Participants may choose to allow their collected data to remain in the study or destoryed |
| NCT05735288 | Yes (Trial name, related institution, ethical approval) | Machine learning | Risk prediction / Diagnostic support(Computer program estimates patient fluid status to help determine optimal dry weight during hemodialysis) | Study involves dialysis patients (chronically ill, potentially vulnerable); participation is voluntary; no specific additional safeguards beyond standard ethical procedures | May help improve accuracy of fluid assessment, leading to better dialysis prescriptions and fewer complications (e.g., cramps, dizziness, heart strain) | No additional risks | 11.89 | 12665 | 122 | NA | Collects bioimpedance data, blood pressure, fluid removal volumes, and other routine dialysis parameters; data used to evaluate the computer program and may be used in future research | Data is pseudonymized, encrypted, and stored securely incloud storage by patient Mpower Ltd.; identifiers kept separately; data not shared with third parties | NA | Yes | NA | Member of the research team | NA |
| NCT06303986 | Yes (Trial name, related institution, ethical approval) | NA | Risk prediction (To predict NAS symptoms and provide suggested treatment) | Study involves newborns (vulnerable); consent obtained from parent/guardian; continuous monitoring with human oversight (nurses input symptoms); device not FDA-approved but investigational | Possible benefits include faster diagnosis and treatment due to continuous monitoring; standardizing NAS diagnosis/treatment for future patients, potentially reducing hospital stay | Minimal risks: wristband discomfort/irritation (unlikely due to biocompatible material); rare device malfunction (with fail-safes); loss of confidentiality. | 11.23 | 9348 | 106 | NA | Data collected includes biometrics (via wristband), video (face blurred), nurse-input symptoms; used to develop predictive analytics and for FDA submission | Data stored on secure network, anonymized/encrypted; video and biometric data viewable on tablet portal but protected | Mentions FDA, U.S. law (ClinicalTrials.gov), Certificate of Confidentiality from DHHS | Yes | Data will be use in the future study without additional consent | NA | Data already collected will continue to be used in the research. |
| NCT06579768 | Yes (Trial name, related institution, ethical approval) | Machine learning | Diagnostic support (To preoperatively differentiate jaw cystic lesions using CT radiomics and assist clinicians in treatment planning) | NA | Indirect benefit: May improve future diagnosis and treatment of jaw cystic lesions by enhancing predictive model performance; no direct benefit to participant | Only notes general data leakage risk | 18.03 | 10020 | 56 | NA | Collects clinical info, CT imaging data, pathology results; used to develop/validate machine learning model for scientific research and publication | States data will be “desensitized,” kept confidential, and protected by law; name/identity not disclosed in reports; access granted to ethics committee, regulatory agencies, and researchers | NA | Yes | NA | NA | NA |
| NCT04577573 | Yes (Trial name, related institution, ethical approval) | NA | Therapeutic support (Uses VR and instrumented wearables for rehabilitation) | Study involves veterans with spinal cord injury (potentially vulnerable); excludes those unable to perform basic hand movements; provides accommodations (e.g., wheelchair access, rest breaks); skin integrity monitored; pregnancy test may be requested for females | Indirect benefit: May help future spinal cord injury patients through improved rehabilitation tools; no direct benefit guaranteed | General risks include skin irritation, falls, visual/neck fatigue, electrical safety, loss of privacy | 9.5 | 23642 | 168 | NA | Collects demographic/medical history, sensory/motor/neurological exam data, muscle activity (EMG), brain activity (EEG), motion trajectories, force production, VR performance, and subjective feedback; data stored in secure repository for current and future research | Data stored in locked file cabinet and on password-protected VA server; identity coded; code kept separately; only IRB-approved personnel access data; future repository adheres to same privacy standards | NA | Yes | Data will be use in the future study without additional consent | Principal Investigator | NA |
| NCT03458806 | Yes (Trial name, related institution, ethical approval) | NA | Diagnostic support (Test whether the data obtained from the Eko Duo and Eko Core Electronic Stethoscope Systems and their heart sound analysis can identify valvular abnormalities found by echocardiography) | NA | No direct benefit to participant; may help improve future diagnosis of heart conditions | No additional risks | 11.79 | 7652 | 80 | NA | Collects electronic heart sound recordings, echocardiogram data, and additional valvular test results; used for research and algorithm development; data linked via unique random identifier | Data de-identified; stored with unique identifier; personal info not included in research dataset; access granted to investigators, UC, sponsor (Eko), and FDA | State and federal privacy laws | Yes | Data will be use in the future study without additional consent | Principal Investigator | NA |
| NCT06915909 | Yes (Trial name, related institution, ethical approval) | NA | NA | NA | NA | NA | 14.55 | 1058 | 7 | NA | States that “relevant sections of my medical notes and data collected during the study may be looked at by individuals from the Sponsor, from regulatory authorities and from the NHS Trust” | NA | NA | Yes | NA | NA | NA |
| NCT05537792 | Yes (Trial name, related institution, ethical approval) | NA | Therapeutic Support (Aims to improve control of powered prosthetic legs using EMG and motion sensors) | Excludes pregnant participants due to fall risk; requires K3/K4 ambulatory level; uses safety harness, rails, and staff support; allows participant to skip any task; skin and residual limb monitored for irritation | No direct benefit to participant; indirect societal benefit: improved prosthetic control for future amputees | General risks include falls, muscle soreness, skin irritation, and metabolic mask discomfort | 13.6 | 18616 | 158 | Yes | Collects EMG signals, motion data, video recordings, metabolic data (energy expenditure), gait metrics; used to develop better control algorithms and for teaching/publication | Video and data stored securely; only PI and research staff have access; videos may be used in teaching/publicationsonly with explicit permission; face can be blocked upon request | NIH as sponsor with right to review records; references U.S. law requirement for ClinicalTrials.gov posting; includes NIH Certificate of Confidentiality | Yes | NA | Principal Investigator | NA |
| NCT04502563 | Yes (Trial name, related institution, ethical approval) | NA | Risk prediction (Predict next hospital admission by looking at vital signs captured on a device and wearable devices to avoid the need for a hospital admission due to worsening heart failure) | NA | Possible benefit: closer monitoring may help avoid hospital admission due to worsening heart failure | General risks include skin irritation and data confidentiality | 12.34 | 20919 | 189 | Yes | Collects continuous vital signs (heart rate, breathing rate, activity) via wearable patch, medical record data, and self-reported health events; used to predict heart failure worsening and trigger clinician alerts | Data encrypted and stored on secure, password-protected servers; medical records kept electronically in secure system overseen by PhysIQ and on paper in locked offices; identifiers removed after study completion | U.S. law requirement for ClinicalTrials.gov posting, and compliance with VA and federal regulations | Yes | Data will be use in the future study without additional consent | Member of the research team | NA |
| NCT06183138 | Yes (Trial name, related institution, ethical approval) | Machine learning | Risk prediction / Diagnostic support (Aims to construct prediction models for common genetic diseases using metabolome data to enable accurate diagnosis and expand tandem mass spectrometry applications) | NA | Indirect benefit: For screen-positive newborns, free Sanger sequencing verification, genetic counseling, and clinical advice provided; broader benefit is improved genetic disease screening and diagnosis | No additional risks | 18.14 | 5228 | 30 | NA | Collects genomic sequencing data (138 genes), tandem mass spectrometry metabolomics data (amino acids, acylcarnitines), and medical records for research, model development, and publication | States medical records are kept per hospital regulations; personal data is confidential; research results will not reveal identity; access granted to ethics committee, health authorities, and sponsor reps for oversight | NA | Yes | NA | Member of the research team | NA |
| NCT05918003 | Yes (Trial name, related institution, ethical approval) | NA | Risk prediction (Predicts daily physical activity / excessive inactivity in COPD patients) | NA | Objective data for care adaptation; validation of algorithm could simplify future assessments and improve quality of life | NA | 14.44 | 7487 | 65 | Yes | Collects clinical data (age, breathlessness, smoking, lung function, meds, comorbidities), lifestyle data (walking, activities), and actimetry sensor data (movement) | Data stored securely on a certified health data hosting server (HDS); anonymized for analysis; only doctor accesses identifiers; code-to-identity key held by Icadom project manager | Explicitly references compliance with the General Data Protection Regulation (GDPR); mentions right to complain to CNIL | Yes | NA | Member of the research team | NA |
| NCT04154904 | Yes (Trial name, related institution, ethical approval) | NA | Risk prediction (Predicts glucose levels based on context to support insulin delivery decisions) | Specific protections for women of childbearing potential: pregnancy testing, requirement for effective contraception; exclusion if pregnant or nursing | No direct benefit to participant; may help develop better diabetes management tools for future patients | General study risks include hypo/hyperglycemia, device-related skin reactions, data breach from lost study phone | 12.41 | 35852 | 308 | Yes | Collects glucose sensor values, fingerstick glucose, insulin pump data, food/rescue carb logs, exercise data, photos, and context data from wearable tags and home beacons | Data stored on a secure OHSU-approved cloud database repository; linked to 3-digit ID; key linking ID to identity is encrypted on restricted OHSU drive; identifiable data removed before external sharing | explicitly mentions compliance with HIPAA, Oregon state law, FDA regulations, Medicare reporting requirements, and references the Oregon Tort Claims Act | Yes | Data will be use in the future study without additional consent | Principal Investigator | Data will not be used and destroyed after withdrawal |
| NCT04184791 | Yes (Trial name, related institution, ethical approval) | NA | Therapeutic support (Further understanding and application of low frequency deep brain stimulation in Parkinson’s patients with gait disorder) | NA | This research may not benefit paticipants directly, will have the opportunity to have motor symptoms comprehensively assessed with wearable sensors. Furthermore, information about this disease or condition may help patients in the future. | Only clinical risks from medication withdrawal, levodopa side effects, and DBS reprogramming | 12.85 | 26189 | 232 | NA | Collects demographic data, medical/DBS history, medication info, sensor-based gait/movement data, MDS-UPDRS scores, brief cognitive assessment, and may access medical records | Data will be de-identified before sharing with collaborators (University of Tennessee, Knoxville); stored indefinitely for research; protected under a Certificate of Confidentiality | Explicitly mentions compliance with U.S. federal law (ClinicalTrials.gov posting requirement), oversight by FDA, DHHS, NIH, and IRB; references Certificate of Confidentiality from DHHS | Yes | Data will be use in the future study without additional consent | Principal Investigator | Data already collected will continue to be used in the research. |
| NCT05231954 | Yes (Trial name, related institution, ethical approval) | NA | Diagnostic support [Help older adults ages 65 and older, through early detection of Alzheimer’s Disease and Related Dementia (ADRD)] | NA | General benefit is early detection of dementia and referral for diagnostic services | General risks like discomfort from questions, blood draw, MRI, or loss of confidentiality are mentioned | 13.84 | 13128 | 110 | NA | Collects questionnaire/interview responses, medical record data, neurological exam findings; may collect blood (2 tbsp) and MRI data if needed for diagnosis | Data protected under a Certificate of Confidentiality from NIH; stored securely with identifiers removed before sharing; access limited to research team, IRB, NIH, and regulatory agencies | Explicitly mentions compliance with U.S. federal law (ClinicalTrials.gov posting), HIPAA, and oversight by NIH, OHRP, and IRB; references Certificate of Confidentiality from NIH | Yes | Data will be use in the future study without additional consent | Principal Investigator | NA |
| NCT05802771 | Yes (Trial name, related institution, ethical approval) | NA | Risk prediction (To develop AI-driven “prediction models” for precision oncology using clinical, radiological, pathological, and genomic data) | NA | No direct benefit | No additional risks | 19.73 | 6975 | 27 | NA | Collection of clinical and disease-related data data (demographic aspects, lifestyle habits,radiological features, pathological and genomic feature) | All the information that will be store in a private and secure "virtual data platform" | NA | Yes | Data will not be use in the future study | Member of the research team | NA |
| NCT05600101 | Yes (Trial name, related institution, ethical approval) | NA | Therapeutic support (Provides care during hospital stay through avatar) | NA | Based on prior research outcomes with similar avatars, this study may benefit participant by engaging them throughout their hospital stay to have a better and more pleasant experience at the hospital and learn more about the transplant process. | Possible emotional distress, disclosure of sensitive personal information may result in a lack of privacy | 13.61 | 19505 | 156 | yes | Data used to operate the avatar and for research on patient engagement includes first name and in-hospital location for safety; meta-data collected | Audiovisual streams are recorded for immediate language processing and understanding but will not be stored post-processing. | Mentions The Health Insurance Portability and Accountability Act (HIPAA) | Yes | NA | Member of the research team | Data already collected will continue to be used in the research. |
| NCT06443073 | Yes (Trial name, related institution, ethical approval) | NA | Therapeutic support (AI system delivers personalized speech exercises and provides real-time feedback to improve articulation and speech intelligibility) | NA | No direct benefit but help researchers to better understand how the different wording of patient reported outcomes (PROs)/questionnaires affect people with speech disorders | No additional risks | 13.95 | 10225 | 85 | NA | Collected personal data and used to train and refine the AI algorithm and evaluate therapy efficacy | In order to protect data, only codes (so-called pseudonyms) are used in the questionnaire. Furthermore, data will only be stored on the server of SoSci Survey (Germany) and the Medical University of Vienna (Austria). | All persons who have access to this data are subject to the applicable national data protection regulations and/or the EU General Data Protection Regulation (GDPR) when handling the data. | Yes | NA | Principal Investigator | Data already collected will continue to be used in the research. |
| NCT06365099 | Yes (Trial name, related institution, ethical approval) | NA | Therapeutic support (Study the effects of a non-invasive brain and nerve stimulation procedure on hand function of stroke patients) | NA | No direct benefit but helpful to develop new treatments | No additional risks | 12.79 | 15723 | 133 | NA | Collect information about participant's motor and mental function, recordings of brain activity using EEG, and recordings of their muscle activity using EMG. | Data will be stored separately from the data collected as part of the project | NA | Yes | Data will be use in the future study without additional consent | Principal Investigator | Data already collected will continue to be used in the research. |
| NCT05506358 | Yes (Trial name, related institution, ethical approval) | Machine learning | Diagnostic support (AI/ML used to automatically detect Sickle cell disease (SCD)/ sickle cell trait (SCT) from blood cell images to find how accurate the 5 low-cost tests are to detect SCD and SCT) | NA | No direct benefit but help people who suffering sickle cell disease in low-income countries in the future) | No additional risks | 12.84 | 16506 | 143 | NA | Data will be analyzed using the code as the main label or reference. Blood samples labels, images, test results, will all be associated only with the unique codes, and not contain identifiable information. | Blood sample will be stored at BC Children’s Hospital | NA | Yes | Data will be use in the future study with additional consent | Principal Investigator | Data will not be used and destroyed after withdrawal |
| NCT03930199 | Yes (Trial name, related institution, ethical approval) | NA | Therapeutic support (Use sensors that are built into a smartphone and sensors worn on the prosthesis, together with a new app that we have developed, to understand how people use their prostheses in everyday life, and what problems prevent or reduce prosthesis use) | NA | To help clinicians improve care for people with amputations and enable people to get the most benefit from their prostheses | Risk of data security, risk of emotional or mental discomfort, risk of soreness or mild discomfort | 13.78 | 26586 | 204 | NA | Collect sensor data and the other testing data to identify areas in which we can help the person do better with their prosthesis... De-identified data from this study may be shared with the research community at large to advance science and health. | De-identified data will be kept indefinitely on password protected servers or in locked cabinets accessible only by authorized researcher staff.Consent forms, personal health information, or any other identifying information will be kept confidential and separate from the data mentioned above in locked cabinets or on password protected servers. It will be destroyed in accordance to IRB policy guidelines unless participants agree to be contacted for future studies. | NA | Yes | Data will be use in the future study with additional consent | Principal Investigator | Data already collected will continue to be used in the research. |
| NCT07159711 | Yes (Trial name, related institution, ethical approval) | NA | NA | NA | NA | NA | 15.13 | 1983 | 12 | NA | May be used to improve the Seismofit algorithm in the future | Anonymised data will be transferred to the Commercial company Ventriject | NA | Yes | NA | NA | NA |
| NCT05789875 | Yes (Trial name, related institution, ethical approval) | NA | Therapeutic support (Try out a mobile health application which will be used to help young adults living with HIV improve adherence to antiretroviral therapy) | NA | No direct benefit | Loss of confidentiality, some of the survey questions may make uncomfortable or upset | 12.02 | 8853 | 83 | NA | NA | NA | Mention Certificate of Confidentiality | Yes | Data will be use in the future study without additional consent | Principal Investigator | Data already collected will continue to be used in the research. |
| NCT06796283 | Yes (Trial name, related institution, ethical approval) | Convolutional Neural Network Algorithms | Risk predicition (Constructing anischemic stroke recurrence risk prediction model within 1, 3, 6, and 12 months using the XGBoost combined with convolutional neural network algorithm) | NA | Through analyzing participant's medical records to individualized personal treatment | No additional risks | 18.06 | 5469 | 29 | NA | Collect various clinical data from subjects, including demographic information, past medical history, laboratory tests, maging studies, clinical medication, clinical diagnosis, antiplatelet drug resistance testing results, neurological function assessment, follow-up data | NA | NA | Yes | NA | Principal Investigator | Data will not be used and destroyed after withdrawal |
| NCT07083791 | Yes (Trial name, related institution, ethical approval) | NA | NA | NA | NA | NA | 16.04 | 1495 | 9 | NA | NA | NA | NA | Yes | NA | Member of the research team | NA |
| NCT04906135 | Yes (Trial name, related institution, ethical approval) | NA | Therapeutic support (Learn about what responses or reactions people have when hearing different sounds, to provide better care for people with hearing loss) | NA | For cochlear implant and hearing aid users, possible benefits to participant's child might be better understanding of factors attributing to his/her hearing performance | No additional risks | 12.72 | 36007 | 317 | NA | Collect basic demographic information (gender, age), medical history, hearing tests, and implant information | NA | Mentions HIPAA, Certificate of Confidentiality | Yes | Data will be use in the future study without additional consent | Member of the research team | NA |
| NCT06762613 | Yes (Trial name, related institution, ethical approval) | Deep learning | Procedural guidance / Interventional support（Constructing a lung puncture guidance system for high-quality CBCT images based on a generative deep learning approach to guide interventional procedures and reduce the number of punctures, radiation dose, and procedural complications.） | NA | Improve the quality of CBCT images generated during interventional procedures with a puncture navigation system to provide a puncture path, better guide the interventionalists to perform the procedure, improve the success rate of the procedure, and reduce the number of intraoperative punctures, surgical complications, and radiation dose to the patient during the procedure. | No additional risks | 16.11 | 4776 | 30 | NA | Collect and archive chest plain CT images beforeparticipants undergo the lung puncture and CBCT images during the surgery for easy viewing at future checkups or review. | At the end of the study,researcher will destroy the forms that contain participants’ information. participants’ images will remain in the radiology department's computer system and researchers will not be able to destroy them | NA | Yes | NA | Member of the research team | Data will not be used and destroyed after withdrawal |
| NCT02990377 | Yes (Trial name, related institution, ethical approval) | Reinforcement learning | Therapeutic support（Adaptive therapeutic support via reinforcement learning to personalize mHealth behavioral interventions for opioid use reduction） | NA | No direct benefit but help researchers develop a program that may help others use opioid medications safely. | Feelings of discomfort as a result of being asked personal questions or providing an optional saliva sample for drug screening and loss of confidentiality. | 13.83 | 26660 | 211 | NA | Once the testing is completed, the saliva sample will be disposed of appropriately. If completed remotely, a member of the study team will instruct participant on how to dispose of the sample. The test results will be documented in the participants’ research records. | Participant personal information will be kept in Ripple™, a secure web application designed for the storing and management of personally identifying information of research participants | NA | Yes | Data will be use in the future study without additional consent | Principal Investigator | Data already collected will continue to be used in the research. |
| NCT05797974 | Yes (Trial name, related institution, ethical approval) | NA | Therapeutic support (To judge the value of a virtual coach for persons with a hernia who are advised to lose weight before any surgery can be carried out) | NA | Possibly improve the preoperative care for hernia patients in the future | No additional risks | 12.84 | 13866 | 125 | NA | Demographic information , results of physical exams (weight), medical history and prior hernia surgeries, area Deprivation Index (a measure of neighborhood disadvantage) | Health information will be stored in locked filing cabinets or on computer servers with secure passwords, or encrypted electronic storage devices, as required by University policy. | NA | Yes | NA | Principal Investigator | Data already collected will continue to be used in the research. |
| NCT06194526 | Yes (Trial name, related institution, ethical approval) | NA | Diagnostic support（Develop a diagnostic method for the detection of cardiovascular disease by means of a blood test） | NA | Participants will be receive a written return (Report), the information obtained from this study will allow to improve the early detection of future diseases in the future. | No additional risks | 16.94 | 20145 | 121 | NA | Collected blood sample data to develop better ways to detect, diagnose, and treat cardiovascular disease/ train artificial intelligence algorithms | Data will be store to Amazon Web Services (AWS) with jurisdiction in the United States of America | Mentions the provisions of Law No. 25.326 | Yes | Data will be use in the future study with additional consent | Principal Investigator | Data will not be used and destroyed after withdrawal |
| NCT06915285 | Yes (Trial name, related institution, ethical approval) | Software algorithms (ASPIRE) | Diagnostic support (The AI analyzes digital retinal images to detect signs of malarial retinopathy and assist in confirming cerebral malaria diagnosis, especially in non-ophthalmic settings) | NA | May lead to a low-cost, smartphone-integrated AI system for accurate cerebral malaria diagnosis in resource-limited settings; reduces need for specialized training; helps prevent misdiagnosis and deaths | No additional risks | 17.07 | 12878 | 72 | NA | Collects digital retinal color images from both eyes using three cameras; data used to develop and validate the ASPIRE algorithm; no patient identifiers collected | Assigns Unique Identifying Number (UNID); master link between name and UNID kept securely by PIs; original forms stored in locked cabinets; data de-identified for analysis/publication; access granted to regulators (NAFDAC, NHREC, U.S. agencies) | NA | Yes | NA | Principal Investigator | Data already collected will continue to be used in the research. |
| NCT06570486 | Yes (Trial name, related institution, ethical approval) | NA | Risk prediction (AI model is used to predict prolonged ICU stay after head and neck cancer surgery) | NA | May help improve future diagnosis/treatment by identifying high-risk patients earlier; contributes to medical research on ICU resource optimization | No additional risks | 16.9 | 7056 | 37 | NA | Collects baseline clinical data: age, gender, BMI, medical history (e.g., hypertension, diabetes), cancer treatment history (radiotherapy/chemotherapy), surgical details, lab results, and imaging data | NA | NA | Yes | NA | Member of the research team | NA |
| NCT06324981 | Yes (Trial name, related institution) | Chatbot | Therapeutic / Behavioral support (AI chatbot delivers tailored health behavior messages (e.g., diet, exercise, smoking cessation) to help manage cardiovascular risk factors; includes pharmacist support arm) | NA | May help improve control of blood pressure, cholesterol, glucose, weight, sleep, diet, and smoking; supports self-management of chronic conditions; contributes to better heart health | NA | 12.83 | 9506 | 96 | NA | Collects responses to text messages, baseline and follow-up survey data (via SMS), and clinical data (e.g., blood pressure, lab values) from medical records; used to assess impact on Life’s Essential 8 scores | NA | NA | Yes | Data will be use in the future study without additional consent | Principal Investigator | Data already collected will continue to be used in the research. |
| NCT05447884 | Yes (Trial name, related institution, ethical approval) | NA | Therapeutic support (Demonstrate the effectiveness of a hip exoskeleton device to improve stability and reduce exertion in participants ) | NA | No direct benefits to participation in the research, the indirect benefit is to help members of the stroke survivor community to improve their balance and reduce exertion during multiple locomotion tasks | Two main risks are that the motor applies a force to the hip causing discomfort and injury, and the possibility of electric shock, also skin irritation due to the use of self-adhesive surface electrodes and risk of fall | 13.66 | 48034 | 375 | NA | Collects demographic info, muscle activity (EMG), movement data (IMUs, motion capture), oxygen consumption (metabolic mask), and video/images of lower limbs during tasks | Data stored with coded ID; linkage file (name ↔ ID) kept password-protected in locked cabinet; digital data encrypted, password-protected, and accessed via VPN; identifiable data shredded when no longer needed | NA | Yes | Data will be use in the future study without additional consent | Principal Investigator | Data will not be used and destroyed after withdrawal |
| NCT06264479 | Yes (Trial name, related institution) | NA | Risk prediction (AI system analyzes imaging of lab-grown microtumours treated with different drugs to predict which therapy may be most effective for the individual patient) | NA | No benefits to take part in this study | No additional risks | 11.67 | 13139 | 124 | NA | Collects clinical data (cancer history, age, gender), tumour biopsy, 40mL blood sample, imaging (CT/PET), and treatment response data; used to validate AI prediction model | Data anonymized with unique code; identifiable info (name, NHS number, DOB) kept securely at local site; de-identified data may be sent outside UK under data protection rules; samples stored for up to 25 years | NA | Yes | NA | Principal Investigator | Data already collected will continue to be used in the research. |
| NCT07169461 | Yes (Trial name, related institution, ethical approval) | NA | NA | NA | NA | NA | 15.84 | 2985 | 20 | NA | NA | NA | NA | Yes | Data will be use in the future study without additional consent | NA | Data will not be used and destroyed after withdrawal |
| NCT06256978 | Yes (Trial name, related institution, ethical approval) | NA | Risk prediction (AI system aims to detect thermal patterns and predict clinical events (e.g., fever, instability) in ICU patients using continuous thermographic imaging) | NA | May improve future temperature monitoring and early detection of clinical deterioration in critically ill patients | No additional risks | 19.32 | 6847 | 36 | Yes | Collects continuous thermographic images, axillary temperature readings, and clinical data from medical records for concordance and pattern analysis | Uses pseudonymization: two databases—one with personal identifiers linked to a code (accessible only to PI/team), and one de-identified for analysis; complies with GDPR and Spanish data protection law | Explicitly cites compliance withEU Regulation 2016/679 (GDPR),Organic Law 3/2018(Spain), andEU Regulation 536/2014; approved by national ethics committee | Yes | NA | Principal Investigator | NA |
| NCT03683472 | Yes (Trial name, related institution, ethical approval) | NA | Therapeutic support (The app delivers mindfulness-based training modules to help users manage anxiety through behavior change techniques informed by AI/adaptive learning) | NA | Free lifetime access to the Unwinding Anxiety app; potential reduction in anxiety; learning about one’s anxiety patterns | A small risk that participant's personal information could be lost or exposed | 12.31 | 10750 | 101 | NA | Collects GAD-7 anxiety scores, app usage data (modules completed), survey responses (including childhood trauma section), and basic contact info for communication | Data stored in secure research database with participant ID; name/contact info stored separately in a password-protected “Key”; signed consent kept under lock and key; data retained ≥3 years | This research is covered by a license called a “Certificate of Confidentiality” from the National Institutes of Health (NIH). | Yes | NA | Principal Investigator | NA |
| NCT05612217 | Yes (Trial name, related institution, ethical approval) | AIVARIX AI-based application | Diagnostic support(The AI app analyzes photographs of lower extremities to detect early signs (CEAP C1–C2) of chronic venous disease) | NA | May increase patient awareness of early venous disease signs and encourage timely consultation with a specialist; no direct clinical benefit to participant | No additional risks | 14.01 | 7104 | 54 | NA | Collects demographic data (age, gender), clinical complaints, physician examination findings, optional ultrasound results, and one photograph of skin area with/without venous changes | Data de-identified using unique participant number; name linked to ID kept securely at research site by physician; sponsor (Servier) may access identifiable data only for quality control; no technical details on encryption or server location | NA | Yes | Data will be use in the future study without additional consent | Principal Investigator | Data already collected will continue to be used in the research. |
| NCT06988189 | Yes (Trial name, related institution, ethical approval) | NA | NA | NA | NA | NA | 16.28 | 1694 | 10 | NA | NA | NA | NA | Yes | Data will be use in the future study with additional consent | Principal Investigator | NA |
| NCT05390684 | Yes (Trial name, related institution, ethical approval) | NA | Risk prediction(The AI/ML-based predictive model aims to identify patients at high risk of postoperative anastomotic leak to enable earlier intervention) | NA | No direct benefit | No additional risks | 13.53 | 8656 | 73 | NA | Collects daily clinical data (vital signs, clinical status) and serial blood samples for inflammatory/infection markers during postoperative hospitalization | Data de-identified using a study code; only the research team can link code to identity; states data stored per hospital protocols butlacks technical details(e.g., encryption, server location, retention period) | Explicitly references compliance withOrganic Law 15/1999 on Personal Data Protection(Spain) | Yes | NA | Principal Investigator | Data will not be used and destroyed after withdrawal |
| NCT06848036 | Yes (Trial name, related institution, ethical approval) | NA | Therapeutic support (AI and digital technologies are used to enable home-based chronic disease monitoring and management for elderly participants) | NA | Participants may receive home nursing and care services; improved chronic disease management; potential health improvement; contribution to future smart care models | No additional risks | 13.53 | 8659 | 73 | NA | Collects demographic data (birthdate, gender, ethnicity) and health information through the smart platform during baseline and follow-up assessments at 3, 6, and 12 months | Data is encoded (de-identified); stored in locked file cabinets accessible only to research team; biological samples (if any) labeled with study code; government/ethics committees may access data per regulations | NA | Yes | NA | Member of the research team | Data will not be used and destroyed after withdrawal |
| NCT06421402 | Yes (Trial name, related institution, ethical approval) | NA | Risk prediction (The study will be built on the basis of an extensive monitoring campaign of chemical and biological pollutants in indoor air, with the purpose of offering affordable and easy-to-implement measures to monitor and improve indoor air quality) | NA | Promoting the early detection of episodes of exacerbation of the disease, as well as an improvement in the management of their disease, increasing empowerment and potentially reducing the use of health resources. | No additional risks | 17.17 | 13783 | 79 | NA | Collects home air quality data (CO₂, VOCs, particles, etc.), lung function (spirometry), vital signs, physical activity, heart rate, sleep quality, health questionnaires, and access to medical records and Catalan health registry | Data pseudonymized with study code; stored securely by Hospital Clínic; encrypted data may be transferred internationally under GDPR safeguards; retention for ≥5 years post-study | Explicitly references compliance withEU GDPR (2016/679),Spanish Organic Law 3/2018, andBiomedical Research Act 14/2007; approved by local CEIm ethics committee | Yes | NA | Principal Investigator | Data will not be used and destroyed after withdrawal |
| NCT06337526 | Yes (Trial name, related institution, ethical approval) | NA | Risk prediction (AI system aims to identify medical, environmental, dietary, or physiologic triggers of pain flares in pediatric CRPS patients using multimodal sensor and self-reported data) | NA | May help identify pain flare triggers to inform future treatment strategies for CRPS; no direct personal benefit guaranteed | No additional risks | 17.31 | 24751 | 130 | NA | Collects questionnaire data, wrist-worn sensor data (heart rate, respiration, activity, sleep), voice recordings, meal photos, limb videos, and optional access to EMR (medical/surgical history, medications, exams) | Data handled via Medeloop platform compliant with NIST 800-53, 800-171, and 800-121 standards; identifiers removed for future research; de-identified data may be shared without re-consent | NA | Yes | Data will be use in the future study without additional consent | Principal Investigator | Data will not be used and destroyed after withdrawal |
| NCT05454514 | Yes (Trial name, related institution, ethical approval) | NA | Therapeutic support (AI system uses facial recognition and video to verify that the participant (not someone else) takes their antiretroviral medication as scheduled) | NA | May help reduce missed/late ART doses, potentially lowering viral load; contributes to future development of smart adherence tools | Risk of private information may be seen by someone other than the researcher. | 13.39 | 9937 | 79 | NA | Collects video recordings of medication ingestion, facial recognition data, medication logs, smartphone alerts, and interview responses about user experience | De-identified data stored on encrypted, password-protected computers or secure cloud storage; video recordings (not de-identified) kept separately and not used for future research; identifiable info stored in locked cabinets | NA | Yes | Data will be use in the future study without additional consent | Principal Investigator | NA |
| NCT04447794 | Yes (Trial name, related institution, ethical approval) | NA | Therapeutic support (The chatbot delivers a behavioral intervention to help users reduce or stop alcohol consumption through interactive messaging) | NA | Free access to evidence-informed digital intervention; may learn helpful strategies to reduce drinking; contributes to future development of alcohol support tools | The questions may be stressful to answer. Participant may experience troubling feelings during the study such as guilt or sadness | 13.54 | 8903 | 68 | NA | Collects self-reported alcohol use, motivation, consequences, treatment history, and optional interview feedback; chatbot interaction logs via Facebook Messenger | De-identified data stored securely; direct identifiers kept separately under controlled conditions; mentionsNIH Certificate of Confidentialitybut lacks technical details (e.g., encryption, server location) | NA | Yes | Data will be use in the future study with additional consent | Member of the research team | NA |

**Supplementary Material 5.** Expert evaluation results

**Evaluation of AI risk scoring framework**

| Dimension | Pengye Xia | Qilin Li | AA | BB |
| --- | --- | --- | --- | --- |
| Model Autonomy | 5 | 5 | 5 | 5 |
| Departure from Standards | 5 | 4 | 5 | 5 |
| Patient-facing Interaction | 5 | 5 | 5 | 4 |
| Clinical Risk | 5 | 5 | 5 | 5 |

Note: Scoring is based on 5-point Likert scale: 1 = Totally Disagree, 2 = Disagree, 3 = Neutral, 4 = Agree, 5 = Totally Agree

**Evaluation of AI risk scoring framework**

| Domain | Requirement | Pengye Xia | Qilin Li | Yuyao Mo | Junmin Cai | Average |
| --- | --- | --- | --- | --- | --- | --- |
| AI-Specific Disclosure | Specify AI type, role, and level of autonomy | 3 | 5 | 4 | 4 | 4 |
|  | Describe departure from standard care, if applicable | 5 | 5 | 4 | 5 | 4.75 |
|  | Explain patient-facing interaction, if relevant | 3 | 5 | 3 | 5 | 4 |
| Risk-Benefit Communication | Disclose clinical, privacy, and algorithmic risks with equal prominence to benefits | 5 | 5 | 5 | 5 | 5 |
|  | Contextualize benefits to avoid therapeutic misconception | 4 | 5 | 5 | 4 | 4.5 |
|  | Acknowledge uncertainties and monitoring plans | 5 | 5 | 5 | 5 | 5 |
| Readability & Accessibility | Limit document length to ≤15,000 characters (~10 min reading time) | 3 | 4 | 3 | 3 | 3.25 |
|  | Achieve SMOG readability score ≤13 (≤10 for high-risk trials) | 4 | 4 | 3 | 4 | 3.75 |
|  | Include at least one comprehension-tested visual aid | 4 | 5 | 3 | 3 | 3.75 |
|  | Offer layered or interactive digital consent options | 4 | 4 | 3 | 5 | 4 |
| Data Governance | Specify data types, storage, access, and retention | 5 | 5 | 5 | 5 | 5 |
|  | Clarify post-withdrawal data handling and offer choice | 5 | 5 | 5 | 5 | 5 |
|  | Disclose future data use and consent mechanisms | 5 | 5 | 5 | 5 | 5 |
|  | Cite applicable protections in plain language | 5 | 5 | 4 | 5 | 4.75 |
| Dynamic Consent & Support | Commit to updates for evolving AI systems | 3 | 4 | 4 | 3 | 3.5 |
|  | Provide multiple, accessible channels for questions | 3 | 4 | 4 | 4 | 3.75 |
|  | Train research staff on explaining AI concepts | 5 | 4 | 4 | 5 | 4.5 |
